# Fatty Acid Synthase induced S6Kinase facilitates USP11-eIF4B complex formation for sustained oncogenic translation in DLBCL

**DOI:** 10.1038/s41467-018-03028-y

**Published:** 2018-02-26

**Authors:** Bandish Kapadia, Nahid M. Nanaji, Kavita Bhalla, Binny Bhandary, Rena Lapidus, Afshin Beheshti, Andrew M. Evens, Ronald B. Gartenhaus

**Affiliations:** 1 Department of Medicine, Marlene and Stewart Greenebaum Comprehensive Cancer Center, University of Maryland, Baltimore, MD 21201 USA; 2 Department of Veteran Affairs, Maryland Healthcare System, Baltimore, MD 21201 USA; 3 University of Maryland Medical Center, Baltimore, MD 21201 USA; 4 Division of Hematology/Oncology, Molecular Oncology Research Institute, Tufts Medical Center, Boston, MA 02111 USA; 5 Veterans Administration Medical Center, Baltimore, MD 21201 USA

## Abstract

Altered lipid metabolism and aberrant protein translation are strongly associated with cancerous outgrowth; however, the inter-regulation of these key processes is still underexplored in diffuse large B-cell lymphoma (DLBCL). Although fatty acid synthase (FASN) activity is reported to positively correlate with PI3K-Akt-mTOR pathway that can modulate protein synthesis, the precise impact of FASN inhibition on this process is still unknown. Herein, we demonstrate that attenuating FASN expression or its activity significantly reduces eIF4B (eukaryotic initiation factor 4B) levels and consequently overall protein translation. Through biochemical studies, we identified eIF4B as a bonafide substrate of USP11, which stabilizes and enhances eIF4B activity. Employing both pharmacological and genetic approaches, we establish that FASN-induced PI3K-S6Kinase signaling phosphorylates USP11 enhancing its interaction with eIF4B and thereby promoting oncogenic translation.

## Abstract (web summary)

Aberrant protein translation and uncontrolled lipid metabolism are hallmarks of cancer growth. Here, in diffuse large B-cell lymphoma, the authors show that fatty acid synthase increases USP11 interaction with and stability of eIF4B via PI3K-S6Kinase signaling, promoting oncogenic protein translation.

## Introduction

With an enhanced understanding of cancer cellular environment and uncontrolled proliferation, it has become evident that tumors rewire their metabolism for sustained growth^1^. This metabolic reprogramming has opened up new opportunities for anti-cancer therapies. Work over the past decade has established altered lipid metabolism as an important metabolic phenotype in cancer cells^2,3^. Diffuse large B-cell lymphoma (DLBCL) cells are consistently noted to be highly addictive to lipids for cellular proliferation, independent of its cell of origin (COO). Hence, expression of fatty acid synthase (FASN), a key enzyme for de novo lipogenesis, is noted to be enhanced in DLBCL^4,5^. Furthermore, inhibiting FASN activity alone or in combination with PI3K inhibitors demonstrated a robust decrease in tumor growth^6,7^. However current FASN inhibitors have limited clinical applications due to certain pharmacological limitations^2^. Given the dynamic nature of FASN regulation, and the complexity in deciphering its downstream mediators, targeting this enzyme with respect to cancer metabolism remains challenging and an area ripe for further investigation.

Over the past decade, regulation of protein translation initiation has emerged as a common downstream node in integrating numerous signaling cascades that are influenced by myriad exogenous/endogenous factors, including nutrients and metabolites^8^. Due to this convergence, controlling the deregulated mRNA translational machinery holds promise for overcoming a major barrier of intra-tumor heterogeneity and multidrug resistance^9^. In fact, targeting eIF4E, a key translational initiation complex (TIC) protein, using chemical inhibitors like Ribavarin has shown potential to reduced tumorigenic growth in xenograft mouse models as well as early clinical trials in AML (acute myeloid leukemia)^10,11^. Joyce et al. studying the translation regulation in melanoma cell lines reported that eIF4A controls 50% of transcripts compared to eIF4E1, which regulated almost 30% of overall transcripts^12^. Importantly, eIF4A inhibitors re-sensitizes lymphomas to DNA-damaging agents in tumors overexpressing eIF4E demonstrating that targeting TIC can overcome chemo-resistance^13^. Willis and colleagues, while studying the altered oncogenic protein translation in DLBCL patients, observed that enhanced activity of eIF4B alone was sufficient for tumor cell survival^14^. Since eIF4B is an indispensable component for cancer cells, its activity is extensively regulated by post-translational modification by the major upstream oncogenic signals, RSK and Akt signaling cascade^15^. Interestingly, the protein levels of eIF4B were elevated in numerous malignancies including DLBCL^14,16,17^; however, no significant alteration of mRNA levels was noted (oncomine database).

The ubiquitin-proteasome system (UPS) plays an important role in the regulation of most cellular pathways, and its deregulation has been implicated in a wide range of human pathologies including cancer^18^. De-ubiquitinating enzymes (DUBs) can reverse the modifications catalyzed by ubiquitin ligases and are noted to be important modulators of numerous cellular processes. For instance, UCH-L1 decreases PHLPP expression leading to prolonged Akt-signaling in lymphomagenesis^19^. Similarly, USP2a stabilizes FASN levels in prostrate cancer^20^. eIF4E is ubiquitinated at Lys^159^ hampering its interaction with eIF4G^21^. Interestingly, eIF4A was reported to be associated with Dpp degradation in drosophila^22^. However, the DUBs associated with TIC are still elusive. In this study, we identified that FASN activity in DLBCL stabilizes eIF4B protein in an USP11-dependent manner. Further, FASN activity induced PI3K-mTORC-S6Kinase signaling phosphorylates USP11. This augmented recruitment of eIF4B-USP11 on the TIC underlies, in part, the sustained oncogenic-translation in DLBCL.

## Results

### Inhibition of FASN activity depletes de novo protein synthesis

**Fig. 1:**
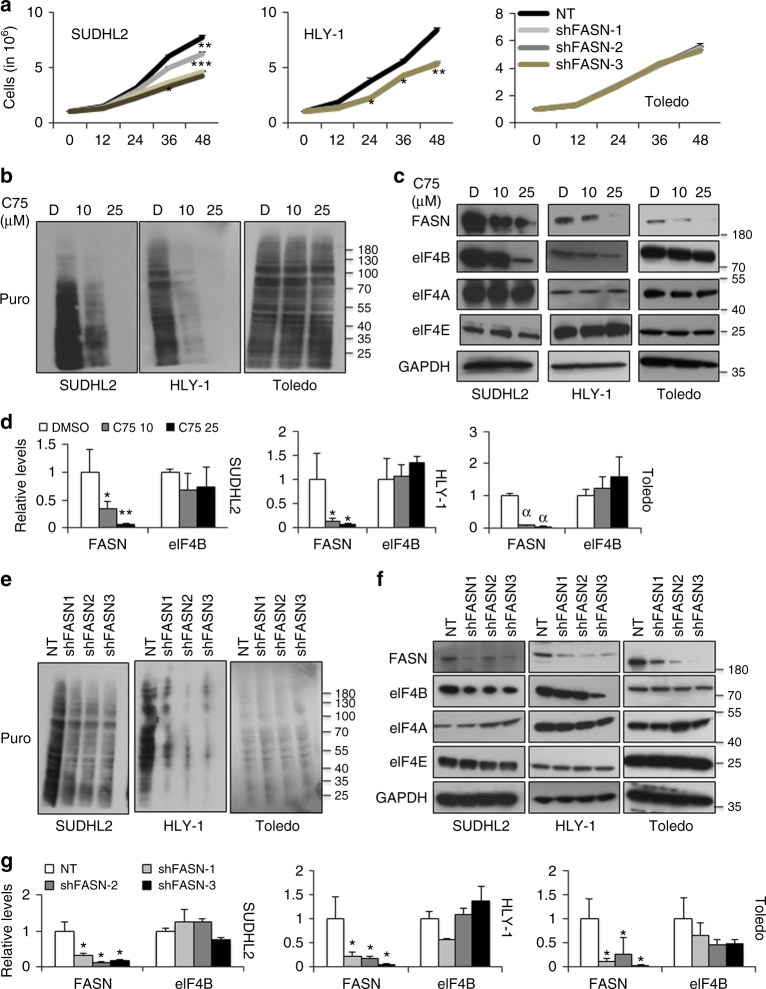
FASN inhibition impedes eIF4B-dependent mRNA translation. **a** Indicated cells were infected with shRNA against FASN or NT (scrambled) and selected on puromycin. Post selection, 1 million were seeded in 6-well plates and counted using trypan blue staining every 12 h for indicated time points. Values are shown as mean ± SD for *n* = 3. Statistical analysis was performed using Student’s *t*-test (unpaired two-tailed), *p < 0.05, **p,0.01, ***p < 0.005 vs. NT infected corresponding cells. **b**, **c** Indicated cells were treated with defined concentration of C75 for 14 h followed by 30 min incubation with puromycin treatment (1 µg/mL) and lysed. Cell lysates were subjected to immunoblotting with the anti-Puro antibody (SUnSET assay) (**b**) or indicated antibodies (**c**). GAPDH was used as loading control. **d** qRT-PCR analysis of FASN and eIF4B expression in indicated cells treated with defined concentration of C75. Results were normalized with DMSO-treated corresponding cells and expressed as mean ± SD (*n* = 3). Statistical analysis was performed using Student’s *t*-test (unpaired two-tailed), *p < 0.05, **p < 0.01, ^α^p < 0.001 vs. DMSO treated corresponding cells. **e**, **f** Indicated cells were infected by shRNA against FASN and cultured in puromycin (0.5–1 µg/mL) for stable cells generation. Post selection, cells were exposed for 30 min with puromycin treatment (3 µg/mL) and lysed. Cell lysates were subjected to immunoblotting with the anti-Puro antibody (SUnSET assay) (**e**) or indicated antibodies (**f**). GAPDH was used as loading control. **g** qRT-PCR analysis of FASN and eIF4B expression in indicated stable cells infected with shRNA against FASN or NT (scrambled). Results were normalized with NT infected corresponding control cells and expressed as mean ± SD (*n* = 3). Statistical analysis was performed using Student’s *t*-test (unpaired two-tailed) *p < 0.05 vs. NT infected corresponding cells

FASN is a known oncotarget, whose expression is enhanced in numerous cancers including DLBCL^2,4,5,23^. We sought to interrogate its impact in regulating another emerging therapeutic target, the protein translational machinery. To address this, we first assessed the impact of inhibiting FASN activity on overall protein translation in DLBCL. We exposed cells to C75, a well-characterized FASN-specific inhibitor and examined its effect on multiple DLBCLs. Consistent with the literature, we noted that inhibiting FASN activity showed significant dose-dependent cell death in ABC-DLBCLs (SUDHL2, TMD8, HLY1) but modest inhibition at higher concentrations in GC-DLBCLs (SUDHL4, SUDHL6, Toledo) (Supplementary Figure 1)^5,9^. Furthermore, knockdown of FASN expression using three different shRNA showed robust reduction in cellular proliferation in ABC-DLBCLs but not in GC-DLBCLs (Fig. 1a, Supplementary Fig. 2A).

To specifically assess the effect of FASN activity in regulating protein translation, we treated DLCBLs (both ABC and GC) with two different C75 concentrations followed by short burst of puromycin employing the SUnSET assay^24^. Impeding FASN activity in ABC-DLBCLs showed significant dose-dependent decrease in de novo protein biosynthesis; however, minimal effects were observed in GC-DLBCLs (Fig.1b, Supplementary Figures 2B, 3A, B). The expression, both at the mRNA and protein level, of FASN was noted to be reduced in C75 dose-dependent manner (Fig. 1c, d, Supplementary Figures 2C, D, 3A, B). Similarly, assessing the impact of mRNA translation in FASN-depleted DLBCLs, we observed that decreasing its expression significantly reduces overall protein translation in ABC- but not in GC-DLBCLs (Fig. 1e, Supplementary Figures 2E, 4A, B). Knockdown of FASN was confirmed by both western blotting and qPCR (Fig. 1f, g, Supplementary Figures 2F, G, 4A, B). These results suggest that suppression of FASN activity has a significant impact on regulating overall mRNA translational machinery in ABC-, but GC-DLBCL were relatively resistant to FASN inhibition.

### Inhibition of FASN activity depletes eIF4B

Having observed a robust reduction in overall protein biosynthesis in ABC-DLBCLs, we next assessed the impact of FASN inhibition on the key protein translational machinery components. Since protein translation is predominantly regulated at the initiation step, we examined the expression of pivotal proteins in TIC. We did not notice any significant changes in the key regulatory enzymes, eIF4A and eIF4E (Fig. 1c, f, Supplementary Figures 2C, F, 3A, B) upon either C75 treatment or FASN depletion in either ABC- or GC-DLBCLs. Interestingly, expression of eIF4B, an auxiliary protein enhancing RNA helicase activity of eIF4A, was decreased in a dose-dependent manner in C75-treated ABC-DLBCLs but no significant changes were noted in GC-DLBCLs (Fig. 1b, Supplementary Figures 2C, 3A, B). However, mRNA levels of eIF4B remained constant (Fig. 1d, Supplementary Figure 2D). A similar trend was also noted upon FASN depleted ABC- but not in GC-DLBCLs (Fig. 1f, g, Supplementary Figures 2F, G, 4A, B).

**Fig. 2:**
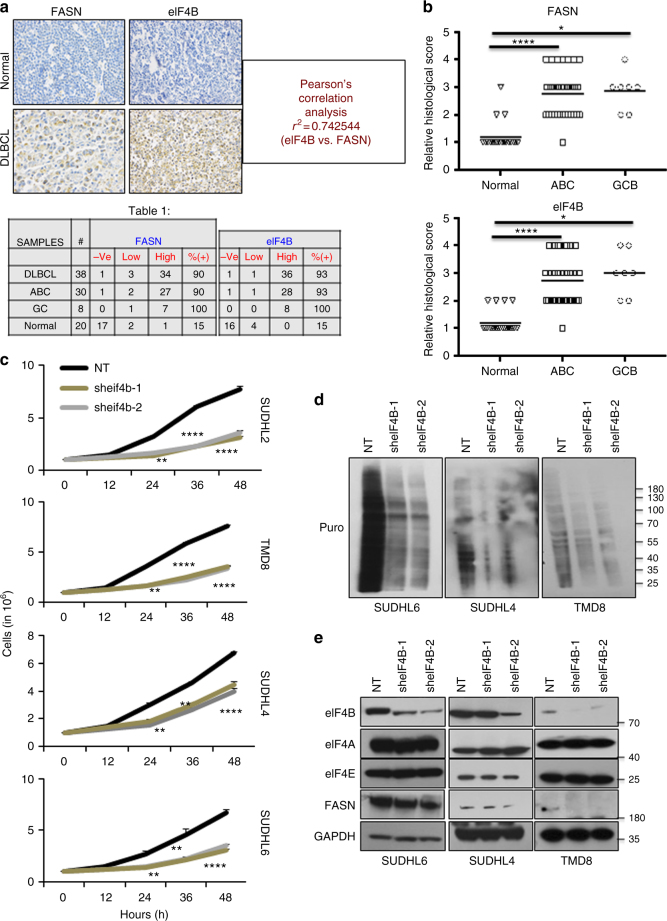
eIF4B is expressed in DLBCL. **a** Representative immunohistochemistry image of TMA slides stained with eIF4B and FASN antibodies. Summary of the eIF4B and FASN stained slides for DLBCL samples. **b** Representative scatter plots showing the stained signals of FASN and eIF4B in normal ABC and GC-DLBCL samples. Statistical analysis was performed using Wilcoxon signed-rank test (unpaired two-tailed), *p < 0.05, ****p < 0.001 vs. normal samples. **c** Indicated eIF4B depleted stable cells were seeded 1 million per well in 6-well plates. Post 12 h, the cells were collected and counted using trypan blue. Values are expressed as mean ± SD (*n* = 3), **p,0.01, ****p < 0.001 vs. NT infected corresponding cells. **d**, **e** Indicated cells were infected by shRNA against eIF4B and cultured in puromycin (0.5–1 µg/mL) for stable cells generation. Post selection, cells were exposed for 30 min with puromycin treatment (3 µg/mL) and lysed. Cell lysates were subjected to immunoblotting with the anti-Puro antibody (SUnSET assay) (**d**) or indicated antibodies (**e**). GAPDH was used as loading control

To determine the possible clinical relevance between eIF4B and FASN in DLBCL, we measured the protein expression in two types of DLBCL (ABC and GC) by immunohistochemistry (IHC) (Fig. 2a). As summarized in Fig. 2b (scattered plot), expressions of eIF4B and FASN were noted to be elevated in greater than 90% as compared with 15% in normal lymph nodes, respectively. Our correlation analysis indicated that eIF4B and FASN expression shows strong positive Pearson’s correlation in primary DLBCL samples. Next, we aimed to study the phenotypic role of eIF4B in DLBCL. Firstly, we depleted the expression of eIF4B using shRNA, which resulted in a strong reduction of cellular proliferation in both ABC- and GC-DLBCLs, this was consistent with the FASN-inhibited ABC-DLCBLs (Fig. 2c). Importantly, eIF4B knockdown in highly proliferative HLY1 (ABC-) cells was noted to be lethal. Next, we studied the impact of eIF4B in clonogenic assays. Consistent with literature and proliferative data, decreasing eIF4B resulted in significant reduction in colony formation in both SUDHL6 and SUDHL2 (Supplementary Figure 5A)^25,26^. As anticipated, inhibiting eIF4B expression significantly reduced global protein biosynthesis (Fig. 2d, Supplementary Figures 5B, 7). Notably, depleting eIF4B expression demonstrated a decrease in FASN expression at both protein and mRNA level in all DLBCLs (*n* = 5) (Fig. 2e, Supplementary Figures 5C-E, 7).

### eIF4B regulates protein abundance of oncogene

**Fig. 3:**
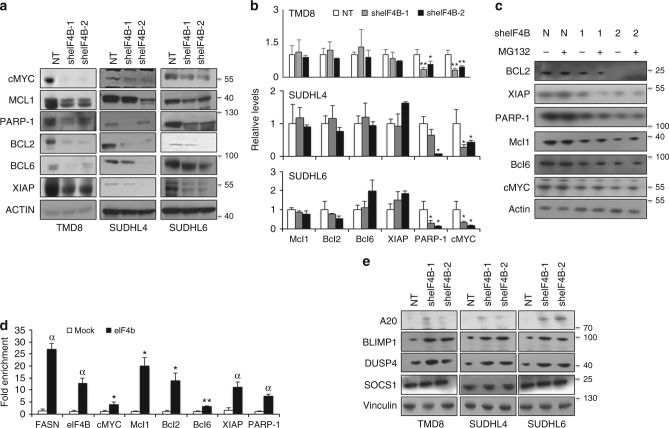
eIF4B regulates translation of oncogenes. **a** Indicated eIF4B depleted stables were lysed and lysates were probed with the defined antibodies. Actin was used as loading control. **b** qRT-PCR analysis for expression of defined genes in indicated stable cells infected with shRNA against eIF4B or NT (scrambled). Results were normalized with NT infected corresponding cells and expressed as mean ± SD (*n* = 3). Statistical analysis was performed using Student’s *t*-test (unpaired two-tailed), *p < 0.05, **p < 0.01 vs. NT infected corresponding cells. **c** Exponentially growing eIF4B depleted stable SUDHL2 cells were cultured with MG132 (10 µM) for 2 h and cell lysates were probed with the defined antibodies. Actin was used as loading control. N: scrambled; 1: sheIF4B-1; 2: sheIF4B-2. **d** Total cell lysates from SUDHL6 were subjected to immunoprecipitation with eIF4B antibodies, followed by RNA isolation and qRT-PCR to detect the enrichment of the defined genes. Results were normalized total RNA from cell lysates and expressed relative percentage to mock sample enriched RNA. Values are expressed as mean ± SD and statistical analysis was performed using Student’s *t*-test, *p < 0.05, **p < 0.01, ^α^*p* < 0.001 vs. corresponding mock samples. **e** Indicated eIF4B depleted stable cells were lysed and lysates were probed with the defined antibodies. Vinculin was used as loading control

The significant reduction in cellular proliferation coupled with decreased nascent peptide biosynthesis upon eIF4B depletion led us to interrogate the mechanistic role of eIF4B expression on oncogenic translation. Initially, we identified a number of target oncogenes, cMYC, MCL1, XIAP, BCL2, BCL6, and PARP-1 whose protein levels were reduced upon eIF4B knockdown (Fig. 3a, Supplementary Figures 6A, 7). Furthermore, mRNA levels of MCL1, XIAP, BCl2, and BCL6 were observed to have minimal changes while both cMYC and PARP-1 messages were decreased in eIF4B-depleted cells (Fig. 3b, Supplementary Figure 6B). To further confirm that expression of certain oncogenes was reduced due to attenuated translation and not protein degradation, we exposed eIF4B-depleted cells to MG132, a potent proteosomal degradation complex inhibitor. Treatment with MG132 showed minimal effect on overall protein levels of the selected oncogenes (Fig. 3c, Supplementary Figure 6C). Furthermore, to examine the mechanistic basis of eIF4B in regulating translation of these oncogenic mRNAs, we performed RNA-IP and assessed its direct interaction by qPCR. Interestingly, RNA enriched upon precipitation of eIF4B showed the presence of all these target oncogenes indicating that eIF4B plays a direct role in their translation (Fig. 3d, Supplementary Figure 6D). Unexpectedly, we observed eIF4B interacting with its own mRNA and thereby potentially auto-regulating its own translation.

Having observed that eIF4B directly regulates the expression of key oncogenes critical for DLBCL cell growth/survival, we next examined their dependency on FASN. Consistent with our previous observations, exposing ABC-DLBCLs to C75 displayed a dose-dependent decrease in eIF4B-dependent oncogenes (Supplementary Figures 8A, 9). A similar trend was also noted in FASN depleted ABC-DLBCLs (Supplementary Figures 8B, 10). However, neither C75 exposure nor FASN depletion in GC-DLBCL impacted eIF4B-dependent oncogene expression (Supplementary Figures 8–10). Together, these data support FASN-eIF4B signaling in regulating biosynthesis of key oncogenes in ABC-DLBCL.

Having observed a robust decrease in potent oncogenes expression, we next aimed to assess the expression pattern of tumor suppressor genes. Depletion of eIF4B resulted in enhanced expression of tumor suppressor genes such as BLIMP1/PRMD1^27,28^, DUSP4/MKP2^29^, and A20/TNFAIP3^30,31^, while the expression of SOCS1^32,33^ was observed to be independent of eIF4B expression (Fig. 2e, Supplemental Fig, 11A, B). Furthermore, mRNA expression of three tumor suppressor genes was also noted to be upregulated in ABC as well as in GC (BLIMP1 mRNA expression was minimally modified in GC-DLBCL, Supplementary Figure 11C). Overall, these data suggest that modifying eIF4B expression/activity alters the expression levels of tumor suppressors and may provide an anti-tumor effect in DLBCL. Consistent with eIF4B depleted DLBCLs, ABC cells exposed to C75 or transduced with shRNA against FASN showed a significant increase in tumor suppressor genes (Supplemental Figures 12A, B, 13A, B). A similar trend (with eIF4B depleted cells) was also noted in the mRNA expression of these genes in those cells (Supplementary Figures 12C, 13C); while no significant changes were noted in GC-DLBCLs upon C75 treatment or depleting FASN expression (Supplementary Figures 12, 13).

### USP11 interacts and deubiquitinates eIF4B

**Fig. 4:**
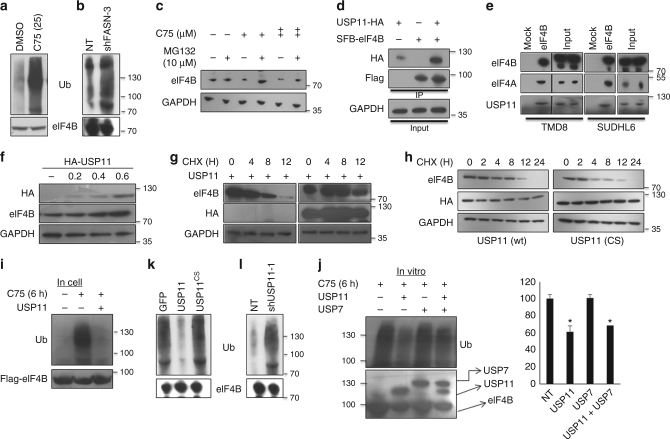
USP11 deubiquitinates and stabilizes eIF4B. **a** SUDHL2 was treated with C75 (25 µM) for 6 h followed by lysis and immunoprecipitation with eIF4B. Lysates were probed with the indicated antibodies. **b** HLY1 cells were transiently infected with shRNA against FASN (sh-FASN3) and lysed after 48 h of infection followed by immunoprecipitation with eIF4B. Lysates were probed for the indicated antibodies. **c** SUDHL2 was treated with C75 (indicated concentration, +: 10 µM, ++: 25 µM) for 14 h followed by treatment with MG132 (10 µM) for 4 h. Lysates were probed with the indicated antibodies. **d** 293T cells were transfected with SFB-eIF4B and/or USP11-HA. eIF4B was enriched by pulling down using streptavidin beads and probed with anti-HA antibodies for detection of USP11. eIF4B enrichment was confirmed by probing with anti-flag antibody. **e** Lysates from TMD8 and SUDHL4 were immunoprecipitated with eIF4B antibody and probed for USP11. **f** 293T cells were transfected with increasing concentration of USP11 and levels of endogenous IF4B was confirmed by immunoblotting. **g**, **h** 293T cells were transfected with either GFP or USP11 (**f**) and USP11 or USP11^cs^ (**g**) and treated with CHX in increasing time point and probed for indicated antibodies. **i** 293T cells were transfected with SFB-eIF4B alone or in combination with USP11 and treated with C75 (25 µM) for 6 h. Post lysis, eIF4B was enriched using streptavidin beads and levels of ubiquitin were detected using specific antibody. **j** TMD8 cells stably expressing the indicated genes were treated with C75 for 12 h followed by lysis and immunoprecipitation with eIF4B. Lysates were probed with the indicated antibodies. **k** SUDHL6 transiently infected with shRNA against USP11 (sh-USP11-1) and lysed after 48 h of infection followed by immunoprecipitation with eIF4B. Lysates were probed for the indicated antibodies. **l** In vitro deubiquitination assay of eIF4B. Polyubiquitinated eIF4B, flag-tagged USP11, and USP7 were enriched from 293T (see the experimental procedure for details) and were incubated with buffered conditions for deubiquitination. Post reaction lysates were probed with the indicated antibodies. Bar diagram represents the densitometric analysis of the ubiquitin signals. Values were normalized with corresponding flag signals and were represented as mean ± SD (*n* = 3). Statistical analysis was performed using Students *t*-test, *p < 0.05, vs. NT (control) samples

We next investigated the molecular mechanisms underlying the decrease in eIF4B protein levels. The decline in eIF4B protein was independent of transcriptional regulation (Fig. 1d, e, Supplementary Figures 2D, E, 3, 4). It was also interesting to observe that eIF4B protein in primary DLBCL samples was noted to be enhanced but minimal changes at the RNA level (Fig. 2a, oncomine database). Therefore, we explored whether eIF4B protein stability is affected by ubiquitination, a post-translational covalent modification associated with proteosomal degradation. Indeed, eIF4B was noted to be ubiquitinated in the presence of C75 but minimally compared with DMSO (dimethyl sulfoxide; 0.1%) (Fig. 4a). Further, depletion of FASN expression in HLY1 cells also enhanced the ubiquitin levels of eIF4B (Fig. 4b). Subsequently, we inhibited the proteasome degrading complex and found that the total eIF4B protein level was partially restored in C75 exposed ABC-DLBCLs (Fig. 4c, Supplementary Figure 14A, B). Taken together, our data demonstrates that depletion of FASN and/or inhibiting its activity degrades eIF4B resulting in decreased oncogenic translation (Supplementary Figure 14C).

To better understand the relationship between FASN activity and increased protein stability of eIF4B, we conducted immunoprecipitation assay to analyze if there was any complex formation between the two proteins. We were not able to identify any complex formation between eIF4B and FASN (Supplementary Figure 14D). In order to decipher potential regulatory molecules stabilizing eIF4B protein levels in DLBCL, we screened the publicly available databases for the protein–protein interactions. Multiple DUBs including USP4, USP7, USP11, and USP45 were found to form complexes with eIF4B (Supplementary Figure 14E). Several putative candidates, USP7, USP45, and USP4, were identified but only USP11 was found to interact and form a complex with eIF4B in two independent studies with high confidence in mass-spec analysis^34,35^.

As USP11 emerged as the strongest candidate DUB that may interact with eIF4B, we experimentally set out to determine its role in enhancing eIF4B stability. To this end, we transfected HEK293T with constructs expressing SFB (S protein binding, Flag and Biotin binding)-eIF4B either alone or with HA-USP11 (Fig. 4d). Immunoblot analysis revealed that HA-USP11 was readily detected in SFB-eIF4B enriched samples. Further, to elucidate this complex formation in more physiological settings, cell extracts from Pfeiffer (ABC) and SUDHL4 (GC) were immunoprecipitated with eIF4B-specific antibody (Fig. 4e). Endogenous USP11 was readily detected in eIF4B-precipitated samples. eIF4A was used as an internal control. To understand the functional consequences of USP11-eIF4B interaction, we first examined whether expression of USP11 modulates eIF4B protein levels. We transfected HEK293T cells with varying doses of USP11 and found that the protein level of eIF4B was enhanced in a dose-dependent manner (Fig. 4f, Supplementary Figure 15A). Furthermore, to buttress our findings, we overexpressed USP11 in a panel of DLBCLs and observed that ectopic expression of USP11 indeed increased basal protein levels of eIF4B. Significantly, enhanced expression of catalytically inactive USP11 (USP11^cs^) showed minimal effect on eIF4B protein levels compared to GFP in DLBCLs (Supplementary Figure 16A, 17). Examining protein turnover using classical cyclohexamide treatment, we noted that overexpression of USP11 (wt) but not USP11^CS^ or GFP expression vector (Figs. 4g, h Supplementary Figure 15B, C) stabilizes the protein. Next, we investigated whether USP11 affects eIF4B ubiquitination. We overexpressed SFB-eIF4B in HEK293T cells in presence/absence of USP11 followed by treatment of C75 for 6 h (Fig. 4i). Post-treatment, eIF4B was affinity purified using streptavidin beads and its ubiquitinylation was markedly reduced in USP11 overexpressing cells. Furthermore, the ubiquitin levels of eIF4B were noted to be minimally enhanced in TMD8 cells overexpressing USP11 and treated with C75 compared to either GFP infected or USP11^CS^ infected corresponding treated cells (Fig. 4k). To further buttress over observations, we depleted USP11 expression in SUDHL6 and observed a significant increase in the ubiquitin levels of eIF4B (Fig. 4l). Next, to demonstrate whether USP11 can directly deubiquitinate eIF4B, we performed in vitro deubiquitinase assays. Polyubiquitinylated eIF4B was purified using streptavidin beads from C75 treated SFB-eIF4B transfected HEK293T lysates, while flag-USP11 and USP7 were purified using flag beads. Performing in vitro deubiquitinase assays, USP11, but not USP7, was noted to deubiquitinate eIF4B (Fig. 4j). Together, these results demonstrate that eIF4B is a bona fide USP11 substrate.

### USP11 stimulates translation

**Fig. 5:**
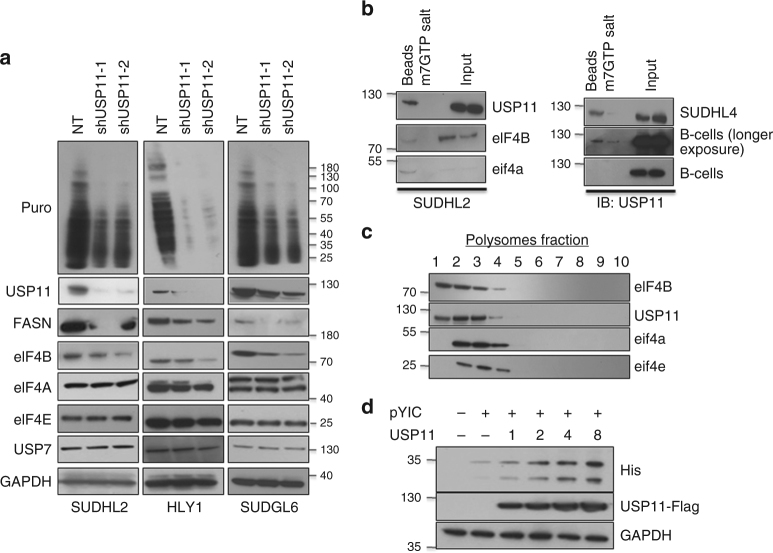
USP11 directly regulates translation. **a** Indicated USP11 depleted stable cells were cultured in presence of puromycin (3 µg/mL) for 30 min and lysates were probed for defined antibodies. GAPDH was used as loading control. **b** Indicated cell lysates were incubated with m^7^GTP sepharose beads in the presence or absence of m^7^GTP salt. Co-eluted proteins were resolved and probed for indicated antibodies. **c** HLY-1 extracts were subjected to sucrose gradient density centrifugation. The gradient was fractioned; fractions were analyzed for indicated antibodies. **d** 293T cells were transfected with pYIC and indicated the amount of USP11 and or GFP. Post-transfection, cell lysates were resolved and probed with indicated antibodies. GAPDH was used as loading control

The interaction of USP11 with eIF4B, a protein that interacts and promotes the activity of eIF4A, implicated its role in regulating de novo peptide synthesis. Also, Li et al. noted that depleting USP11 significantly reduces IRES-mediated translation in HCV-infected cells^36^. To test the efficacy of USP11 on overall mRNA translation, we pulse-labeled USP11-depleted ABC- and GC-DLBCLs with puromycin followed by probing with anti-puromycin antibody^24^. In both subtypes of DLBCL, we observed that USP11 knockdown cells had a significant reduction in overall protein biosynthesis compared to scr-infected corresponding DLBCLs. In order to confidently rule out non-specific effects of shRNA, we employed two additional shRNA controls (one targeting cDNA and another targeting 3′UTR) for the current study (Fig. 5a, Supplementary Figure 18A, 19). Parallel experiments revealed that protein levels of both eIF4B and FASN were significantly reduced in USP11-depleted cells, while there were minimal changes observed in eIF4B mRNA levels. Unexpectedly, the mRNA levels of FASN were greatly reduced (Fig. 5a, Supplementary Figure 18B). In a reciprocal experiment, we overexpressed USP11 in DLBCL and assessed overall protein biosynthesis. Consistent with our observation, overexpression of USP11 but not USP11^CS^ resulted in enhanced protein synthesis along with eIF4B protein levels (Supplementary Figures 16A, 17). However, USP7 knockdown showed minimal effect on eIF4B protein levels (Supplementary Figure 16B).

To test whether USP11 can directly interact with the TIC, we next performed cap pulldown assays using cap-binding m^7^GTP immobilized beads. USP11 along with eIF4B and eIF4A were readily detected in m^7^GTP pull down of SUDHL2 lysates, but the addition of competing m^7^GTP nucleotide abrogated the signals (Fig. 5b). Furthermore, the presence of USP11 in TIC was also observed in SUDHL4 and primary B-cells (Fig. 5b). We next performed ribosomal profiling of HLY1 lysates and found that USP11 co-fractioned with the TIC, which was consistent with our cap binding assays (Fig. 5c). Having observed that USP11 is recruited to cap-binding pre-initiation complex, as well as Li et al. reported USP11 impact on IRES-dependent translation^36^; we next assessed the USP11 on different translation functions: cap-dependent and cap-independent translation using bicistronic translational reporter construct (pYIC): CMV driven cap-dependent His-YFP in cis with an IRES-dependent His-CFP^37,38^. Overexpression of either eIF4B or USP11 significantly enhanced both cap-dependent as well as IRES-mediated translation in a dose-dependent manner (Fig. 5d, Supplementary Figure 16C). It is also important to note that overexpression of eIF4B enhanced endogenous protein level in a dose-dependent manner as well. These data strongly suggest that USP11-eIF4B can activate both cap-dependent as well as IRES-driven translation. Having noticed that USP11 stabilizes eIF4B protein levels and eIF4B regulates the loading of proto-oncogene mRNA like cMYC and Bcl2, it was tempting to assess whether USP11 mRNA loading on the ribosomal complex is regulated by eIF4B. For this, we analyzed the USP11 mRNA interaction with eIF4B. Interestingly, mRNA enrichment upon eIF4B precipitation showed minimal increase (non-significant) of USP11 and USP7, however the protein of USP11 was noted to be marginal enhanced in eIF4B depleted cells, indicating the USP11 and USP7 expression were independent of eIF4B expression (Supplemental Fig. 16D, 16E).

### USP11 depletion decreases DLBCL cell proliferation

**Fig. 6:**
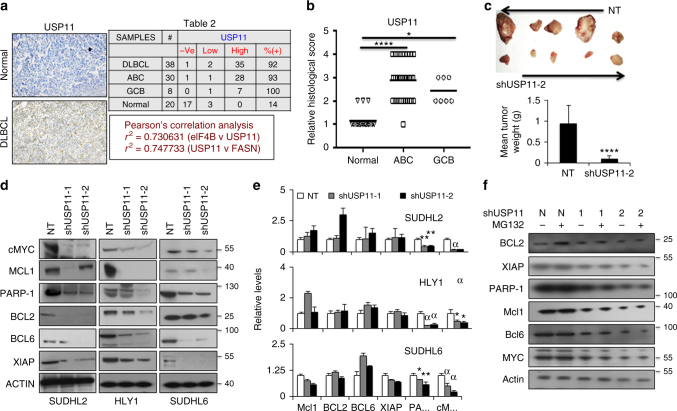
USP11 promotes DLCBL tumorigenesis. **a** Representative immunohistochemistry image of TMA slides stained with USP11 antibodies. Summary of the USP11 stained slides for DLBCL and normal reactive lymph node samples. **b** Representative scatter plots showing the stained signals of USP11 in normal ABC and GC-DLBCL samples. Statistical analysis was performed using Wilcoxon signed-rank test (unpaired two-tailed), *p < 0.05, ****p < 0.001 vs. normal samples. **c** Images of HLY-1 derived (NT and shUSP11-2 infected cells) tumors and quantified tumor weights. Bar diagram represents mean weight of the tumors. Values were expressed as mean ± SD and statistical analysis was performed using Student’s *t*-test, ****p < 0.001. **d** Indicated USP11 knockdown cell lysates were probed with indicated antibodies. Actin was used as loading control. **e** qRT-PCR analysis for checking mRNA expression of indicated genes in USP11 knockdown cells. Results were normalized with NT infected corresponding cells and expressed as mean ± SD (*n* = 3). Statistical analysis was performed using Student’s *t*-test (unpaired two-tailed), **p < 0.01, ^α^*p* < 0.001 vs. NT infected corresponding cells. **f** USP11 depleted SUDHL2 cells were treated with MG132 (10 µM) for 2 h and lysates were probed with the indicated antibodies. Actin was used as loading control

To clarify the clinical relevance of USP11 in lymphomagenesis, we examined the expression of USP11 in primary DLBCL specimens. IHC analysis of USP11 expression in 38 DLBCL as well as 20 reactive lymph nodes markedly displays >90% expression in tumor (Fig. 6a, b). Replicate IHC slides used for USP11 staining were also examined for eIF4B and FASN expression. Performing Pearson’s correlation analysis, we found that there is a strong positive correlation in the expression pattern of all three proteins. USP11 along with eIF4B and FASN appear to be elevated in the vast majority of DLBCL independent of its COO.

Since USP11 protein levels were elevated in DLBCL and were found to be critical for eIF4B stability, a key regulatory molecule in cellular translation/transformation, we examined its effect on the cellular phenotype. We compared the cellular proliferation in USP11-depleted DLBCL with scr-shRNA. There was a significant decrease in the cellular division in both ABC- and GC-DLBCLs upon USP11 knockdown (Supplementary Figure 20A). Similar results were also obtained in depleting either eIF4B or FASN (in ABC-DLBCLs). Furthermore, USP11-depleted cells (HLY1) transplanted into SCID mice showed significant growth impairment compared to scr-infected control cells (Fig. 6c). Moreover, a similar trend was noted when carrying out in vitro colony formation assays (SUDHL6) (Supplementary Figure 20B). Together, these results support a role for USP11 in the survival and tumorigenicity of DLBCL.

Having noticed a major reduction in cellular proliferation coupled with overall protein translation, prompted us to assess the expression of eIF4B-dependent oncogenes in USP11 silenced DLBCLs. As shown in Fig. 6c (and Supplementary Figures 21A, 22), knockdown of USP11 resulted in significant decrease in expression of most eIF4B-dependent oncogenes. Consistent with eIF4B-depleted cells, mRNA levels of BCL2, BCL6 XIAP, and MCL1 were noted to have minimal changes while that of cMYC and PARP-1 were significantly reduced (Fig. 6d, Supplementary Figure 21B). Further, culturing USP11-knockdown DLBCLs with MG132 showed minimal changes in eIF4B-sensitive oncogenes protein levels indicating that USP11-eIF4B deficient hampered translation resulted in decrease oncogene protein biosynthesis (Fig. 6e, Supplementary Figure 21C). Reciprocally, upon overexpression of USP11, but not USP11^CS^, we demonstrated an overall increase in protein levels of eIF4B-dependent genes (Supplementary Figures 23, 17). Next, we examined the expression of various relevant tumor suppressor genes. Consistent with the previous observation (Fig. 2e, Supplemental 11), USP11 knockdown cells also displayed enhanced expression of tumor suppressor genes (BLIMP1, A20, and DUSP4) (Supplementary Figure 24A, B). Similarly, the RNA levels of these genes were also enhanced (BLIMP1 mRNA was minimal modified in GC-DLBCL) (Supplementary Figure 24C). Reciprocally, upon overexpression of USP11, but not USP11^CS^, we demonstrated an overall decrease in protein levels of eIF4B-dependent tumor suppressor genes (Supplementary Figure 25). Together, these data strongly suggest that USP11 promotes DLBCL proliferation, in part by enhancing protein translation of eIF4B-dependent oncogenes and diminishing tumor-suppressor gene expression.

### DLBCL cells are susceptible to USP11 inhibition

**Fig. 7:**
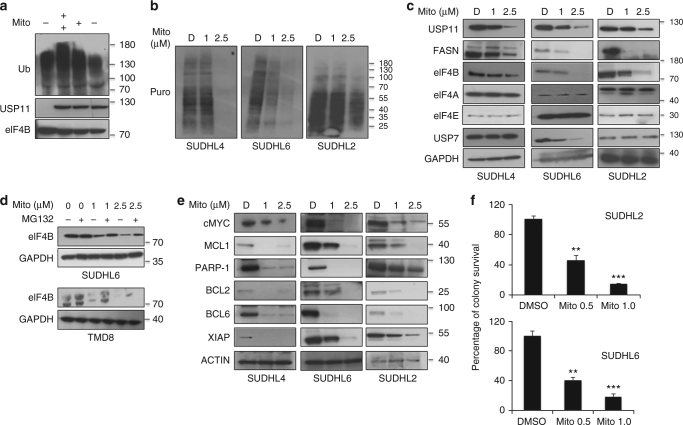
Pharmacologically blocking USP11-mediated deubiqtuination in DLBCL. **a** In vitro deubiquitinase of eIF4B assay. Incubation of polyubiquitinated eIF4B with enriched USP1-HA in presence of increasing concentration of Mitoxantrone (Mito). Lysates were resolved and probed for indicated antibodies. **b**, **c** Indicated cells were cultured with Mito (indicated concentration) for 14 h. Post treatment, cells were incubated with puromycin (1 μg/mL) and lysates were probed with anti-puromycin for SUnSET assay (**b**). Same lysates were also probed for indicated antibodies (**c**). GAPDH was used as internal loading control. **d** SUDHL6 and TMD8 were treated with Mito (indicated concentration) for 14 h followed by treatment with MG132 (10 μM) for 2 h. Lysates were probed for the indicated antibodies. GAPDH was used as loading control. **e** Lysates (From 7B) were resolved and probed for indicated antibodies. Actin was used as loading control. **f** Percentage colony survival of SUDHL6 and SUDHL2 treated with Mito (indicated concentration), normalized to vehicle control DMSO (0.1%), **p < 0.01, ***p < 0.005

Brody and colleagues using in vitro fluorescence-based assay reported Mitoxantrone as a USP11 inhibitor with an IC^50^ of 3.15 µM^39^. In order to corroborate our USP11-knockdown findings, we tested whether USP11 inhibitor, mitoxantrone, also suppresses USP11-stabilized eIF4B. We first determined whether mitoxantrone inhibits USP11 deubiquitinase activity. The ability of USP11 to remove ubiquitin moieties from eIF4B was blocked in a dose-dependent manner (Fig. 7a). Next, we treated DLBCLs with mitoxantrone at varying concentrations and noted that nascent peptide synthesis coupled with levels of eIF4B were decreased with minimal change in eIF4A or eIF4E levels (Fig. 7b, c Supplementary Figures 26A, B, 27). Moreover, mRNA levels of FASN and USP11, but not eIF4B, were found to be reduced in a dose-dependent manner (Supplementary Figures 26C, 28). Furthermore, levels of eIF4B were partially restored by treating with MG132, indicating that eIF4B undergoes proteosomal degradation (Fig. 7d Supplementary Figure 26D). Importantly, the expression of eIF4B-dependent anti-apoptotic proteins was also reduced (Fig. 7e Supplementary Figures 26E, 27). Consistent with our USP11-depleted cells, mitoxantrone treatment displayed minimal effect on total mRNA levels of the target oncogenes suggesting a post-transcriptional/translational mechanism underlying the effect of interrupting the USP11-eIF4B axis (Supplementary Figures 26F, 28). Likewise, the protein and mRNA levels of tumor suppressor genes were both found to be increased which is consistent with USP11 depleted cells. These findings illustrate the broad regulation of cellular proliferation due to the above manipulations. Finally, mitoxantrone suppressed colony formation in both ABC- (SUDHL2) and GC-DLBCL (SUDHL6), in line with our earlier observations (Fig. 7f). Furthermore, expression pattern (protein and mRNA) of the tumor suppressor genes was noted to be enhanced in a dose-dependent manner in mito-treated DLBCLs (Supplemental Fig 29), which was consistent with USP11 depleted cells. Together, these data demonstrate that USP11 inhibition effectively suppresses DLBCL growth through the destabilization of eIF4B.

### p70-S6Kinase phosphorylated USP11 regulates eIF4B stability

FASN inhibition was shown to degrade eIF4B in ABC- but not in GC-DLBCLs. USP11 activity was also found critical for eIF4B protein stability. This prompted us to investigate the molecular basis for this distinct subset difference of FASN activity directed USP11 stabilizes eIF4B in DLBCL. Therefore, we assessed the expression of USP11 in C75 treated as well as FASN knockdown DLCBL. The expression of USP11, but not USP7, was observed to decrease in C75 treated cells as well FASN knockdown ABC-DLBCLs (Supplementary Figures 30, 31). A potential mechanism of enhanced FASN activity with sustained tumor growth is by direct activation of cellular survival signaling pathways like Wnt/ß-catenin or oncogenic signaling pathway like PI3K/Akt. To test the impact of Wnt signaling in FASN regulated USP11/eIF4B axis, we exposed cells to Wnt antagonist and examined the levels of USP11 and eIF4B. Interestingly, blocking Wnt signaling had substantial impact on overall protein translation but no significant effect on expression of eIF4B or USP11 in DLCBL cells (Supplementary Figure 32A).

**Fig. 8:**
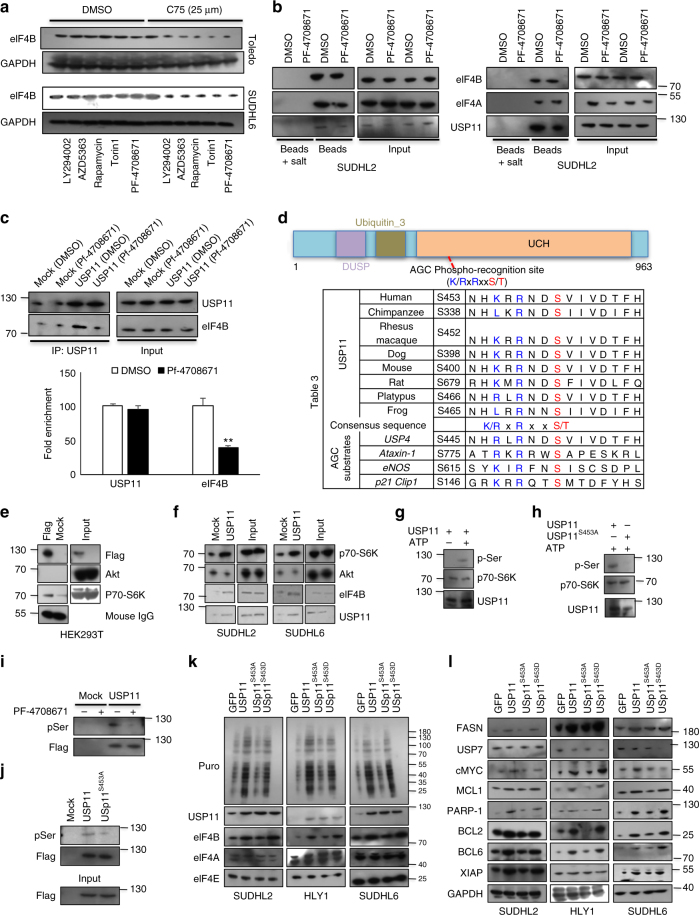
S6Kinase-mediated USP11 phosphorylation regulates its interaction with eIF4B. **a** Toledo and SUDHL6 were treated with indicated PI3K signaling inhibitors (LY294002: PI3K inhibitor (1 µM), AZD5363: Akt inhibitor (500 nM), Rapamycin: mTOR inhibitor (50 nM), Torin 1: mTOR inhibitor (250 nM), PF-4708671: S6Kinase inhibitor (1 µM)) in presence or absence of C75. Post treatment, cell lysates were probed for eIF4B. GAPDH was used as loading control. **b** Indicated cells were treated with PF-4708671 (S6Kinase inhibitor: 1 µM) for 4 h. Post-treatment cell lysates were incubated with m^7^GTP-sepharose beads in presence or absence of m^7^GTP salt. Co-eluted proteins were resolved and probed for indicating antibodies. **c** PF-4708671 (S6Kinase inhibitor: 1 µM) treated SUDHL4 cell lysate was subjected to immunoprecipitation with USP11 antibody and probing eIF4B assessed its interaction. **d** Schematic presentation of protein architecture of USP11. Sequence alignment of AGC substrate consensus sequences within USP11 across different species along with known substrates like USP4, Ataxin-1, eNOS, and p21 Clip1. **e** 293T cells were transfected with flag-USP11. Cell lysates were incubated with flag beads and co-eluted proteins were detected by immunoblotting. **f** SUDHL2 and SUDHL6 lysates were subjected to immunoprecipitation with USP11 antibody and co-eluted protein complex was probed with indicated antibodies. **g**, **h** Purified USP11 or HA enriched USP11 and its mutants were incubated with active S6Kinase protein in presence and absence of ATP as indicated and phosphor-signals of modified USP11 were captured using pSer antibody. **i**, **j** 293T cell transfected with HA-USP11 (**i**) and/or its mutant (**j**) and cultured in 20% FBS. Post-transfection, USP11 was enriched with HA beads and phosphor-signals were captured using pSer antibody. **k**, **l** DLBCL cells infected with USP11 (wt and indicated mutants) were cultured in presence of puromycin (3 µg/mL) for 30 min and lysed. GFP infected cells were used as corresponding controls. Total cell lysates were resolved and probed with indicated antibodies. GAPDH was used as loading control

Several groups previously demonstrated that inhibiting PI3K activity considerably improves the efficacy of FASN inhibition in numerous malignancies, including DLBCL^40^. Moreover, GC-DLBCL (FASN-resistant) cells are addictive to PI3K signaling due to suppression and/or deletion of PTEN expression. Not surprisingly, co-treatment of LY294002 (PI3K inhibitor) along with C75 reduced both eIF4B as well as USP11 in GC-DLBCL (Supplementary Figure 32B). A sequential co-inhibition of PI3K signaling (PI3K, Akt, mTOR, and p70-RSK) along with C75 indicated that constitutively active PI3K signaling regulates USP11 mediated eIF4B protein stability (Fig. 8a, Supplementary Figure 32C). Particularly, inhibition of PI3K or p70-RSK in GC and/or ABC-DLBCLs reduced USP11 recruitment to TIC along with its eIF4B interaction (Fig. 8b, c, Supplementary Figure 32D). Moreover, phosphorylation of p70-RSK was noted to be reduced in ABC-, but not in GC-DLBCLs, upon either C75 treatment or FASN depletion, indicating that active p70-RSK may be driving the USP11-eIF4B interaction for sustained oncogene expression (Supplementary Figure 33). To decipher this putative novel signaling, we performed USP11 protein sequence analysis and observed that the protein encompasses a consensus AGC-substrate motif (RxRxx**S/T**) at Ser^453^, which was conserved across species (Fig. 8d). Interestingly, we also noted that USP11 interacts with S6Kinase but not with Akt in 293T as well as in DLBCLs (Fig. 8e, f). Performing in vitro kinase assays, we observed that S6Kinase was able to modify USP11, which was completely lost on phospho-deficient mutation at Ser^453^ (USP11^S453A^) (Fig. 8g, h). To examine whether USP11 is indeed S6Kinase substrate in tissue culture, we overexpressed USP11 and assessed the phosphorylation of USP11 using phospho-specific antibody that recognizes the optimal pSer substrate. Consistent with our in vitro data, inhibiting S6Kinase or overexpressing phospho-deficient mutant of USP11 showed a much-attenuated phospho-signal compared to the corresponding control (Fig. 8i, j). This indicates that S6Kinase phosphorylates USP11 at Ser^453^.

To study the effect of S6Kinase mediated phosphorylation on USP11 activity, we also overexpressed USP11 (WT and its mutants) and assayed for eIF4B protein. Ectopic expression of USP11 (W) or USP11^S453D^, but not USP11^S453A^, enhanced the overall mRNA translation in DLBCLs (Fig. 8k, Supplementary Figures 34A, 35). Consistently, the protein levels of eIF4B, FASN, and eIF4B-dependent oncogenes were also noted to be enhanced in USP11 (wt and Asp mutant) but not in USP11^S453A^ overexpressing cells (Fig. 8l, Supplementary Figures 34B, 35). Furthermore, the protein levels of tumor suppressor genes (A20, BLIMP1, and DUSP4) were also reduced upon ectopic expression of USP11 (wt and USP11^S453D^) but not with USP11^S453A^ overexpression (Supplementary Figure 36). Next, we examined the effect of S6Kinase mediated phosphorylation on USP11 deubiquitinase activity. Performing in vitro assays, we observed no significant change in ubiquitin signals of eIF4B in presence either wt or both mutants of USP11 (Ser^453A/D^) (Supplementary Figure 37A). Remarkably, assessing deubiquitination levels of eIF4B in cell-based assays we found that USP11 (wt and Asp mutant) was able to deubiquitinate eIF4B compared with GFP-transfected cells, which did not occur for USP11^S453A^ (Supplementary Figure 37B). In light of this apparently conflicting observation based on in vitro and cell-based deubiquitination assays, we next examined whether the interaction between USP11 and eIF4B was S6Kinase-dependent. As seen earlier, USP11, as well as its phospho-mimetic mutant, was readily detected in eIF4B-enriched samples, while the interaction of USP11^S453A^ was moderately reduced (Supplementary Figure 37B). These results imply that S6Kinase mediates USP11 phospho-modification at Ser^453^, in so doing enhancing its interaction with eIF4B, thereby promoting its stability and stimulating eIF4B-dependent oncogenic translation.

## Discussion

**Fig. 9:**
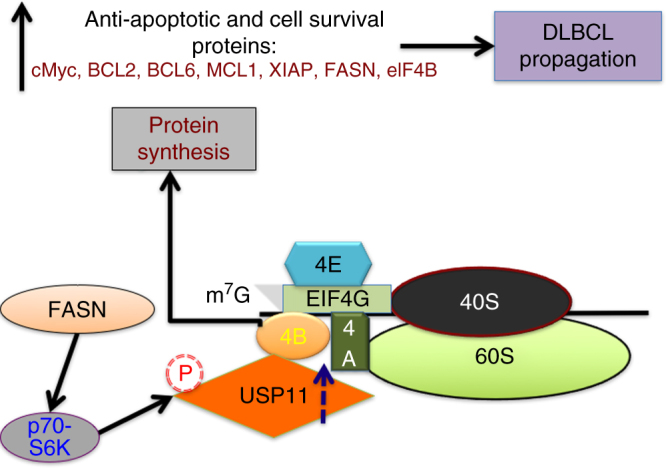
Oncogenic translation in DLBCL is coordinately regulated by FASN/eIF4B/USP11 axis. Enhanced FASN activity stimulates S6Kinase to phosphorylate USP11 at Ser^453^. This phospho-modified USP11 is recruited to translation initiation complex where it interacts, deubiquitinates, and stabilizes eIF4B protein levels. Enhanced eIF4B protein increases mRNA translation of oncoprotein like cMYC, BCL2, BCL6, and others, which are the major drivers for lymphomagenesis

Over the past few years, there has been a growing interest in studying the interplay between metabolism and translation with regards to the transformed phenotype. Important oncogenic drivers such as cMYC and the PI3K-mTOR pathway are known to regulate both translation as well as metabolism^41^; however, the direct impact of metabolism on protein translation or vice versa is still poorly understood. In this study, we demonstrated that FASN, a key metabolic enzyme, plays a positive role in augmenting eIF4B-dependent oncogenic protein translation, which contributes to lymphomagenesis. We provide compelling evidence that the level of eIF4B protein is enhanced in a FASN/S6Kinase linked manner in DLBCL. We further demonstrated that USP11 activity stabilizes eIF4B protein levels and stimulates oncogenic translation. Finally, the interaction between eIF4B and USP11 is directly modulated by FASN-mediated S6Kinase activity (Fig. 9).

In our initial observations, we noted that regulating FASN activity by the small-molecule inhibitor, C75, or RNAi-mediated depletion resulted in a dramatic decrease in overall protein translation of ABC-DLBCLs. It is well documented that curtailing FASN activity limits the PI3K/Akt signaling cascade, a master regulator of translational machinery^2,5^. However, there is scant data in the literature, to the best of our knowledge, that FASN activity has any direct effect on protein translational machinery. Assessing the translational machinery components, we observed a robust decrease in eIF4B protein levels in FASN inhibited ABC cells. Similarly, inhibition of either EGFR or receptor tyrosine kinase (both the receptors are known to be stabilized by FASN activity) resulted in decreased eIF4B protein levels supporting our hypothesis that of FASN downstream induced signals enhances eIF4B protein levels^25,42,43^. Consistently, Uddin et al. noted that attenuating FASN activity leads to significant decrease of c-MET tyrosine receptor kinase expression, whose translation is also regulated by eIF4B^5,44^. Similarly, others and we noted that the expression of cMYC, a master regulator of metabolic genes expression including FASN^45^, is regulated by eIF4B activity^46^. Recently it was reported that PARP-1, a key protein in DNA-damage repair pathway, is transcriptionally regulated by FASN/NF-ĸB/Sp1 axis^47^. Further, our results also suggest/indicate that PARP-1 expression is also controlled at the translational level by eIF4B/FASN axis, which in part provide mechanistic insights in explaining the correlation between higher FASN expression and resistance to genotoxic drugs in chemotherapy^2,48,49^. It is also important to note that FASN depletion/inhibition had minimal effect on eIF4B protein and overall protein translation in GC-DLBCLs.

Aberrant protein translational machinery has been implicated in cancer development and progression for a number of years^9^. Consistent with observations by Horvilleur et al., we also found that eIF4B appears to be essential for DLBCLs (ABC and GC) proliferation that was independent of its COO^14^. Importantly, positive correlation between FASN and eIF4B at the protein level in DLBCL samples, indicates the complex interplay between rewired metabolic signals and translational machinery for driving lymphomagenesis. In fact, eIF4B seems to have a direct impact of translational regulation of oncogenes like BCL2, BCL6, XIAP, and MCL1, which is consistent with previous literature^14,17^, while the expression of FASN, cMYC, and PARP-1 appears to be regulated at both transcriptional and translational levels. It is also interesting to note that protein levels of eIF4A were not altered in DLBCL^14^, but its activity is augmented through BCR-signaling coupled with increased eIF4B levels^50^. Reciprocally, eiF4B depletion also enhanced the expression of tumor suppressor genes like A20, BLIMP1, and DUSP4 in DLBCLs. It should be also noted that BLIMP1 protein levels were upregulated, but not mRNA in GC-DLBCLs upon eIF4B indicating a potent translational control of BLIMP1 expression in DLBCLs for sustained proliferation. Interestingly, the expression of SOCS1 appears to be regulated independent of eIF4B/USP11/FASN axis in DLBCL. Thus, eIF4B appears to have robust control in enhancing aberrant oncogenic-protein translation in DLBCL providing a sound rationale for developing targeted chemical inhibitors to translational regulatory molecules.

In our interrogation of the molecular basis for sustained protein levels of eIF4B in DLBCL, we identified USP11 as a strong potentiator of eIF4B-promoting translation and showed that it deubiquitinates and stabilize eIF4B; however, the de novo protein synthesis of USP11 was independent of eIF4B activity. USP11 has been implicated in tumorigenesis with its function clarified in the past few years; including NF-ĸB activation and double-stranded DNA-damage repair pathway^51–53^ (which are also strongly/positively correlated with FASN activity^47^). One study suggested a potential role of USP11 in regulating IRES-mediated translation^36^. In fact, our detailed analysis indicates that USP11 is recruited to TIC and regulates both cap-dependent as well as IRES-mediated translation. USP11 has been reported to stabilize oncogenic proteins BRCA2 as well as XIAP^51,54^, which are also regulated at the translational level by eIF4B^14^. Likewise, silencing USP11 has been shown to sensitize cells to PARP inhibitors^55^. Indeed, we noted modulating USP11 levels has a direct impact on PARP-1 expression. Also, certain DUBs including USP7 and USP11 are known to form complexes with each other^56,57^. Based on RNAi and in vitro deubiquitination data, we found that USP11 but not USP7 was able to enhance protein levels of eIF4B, confirming eIF4B as a bona fide substrate of USP11. Our observation that depletion of USP11 or inhibition using a non-specific inhibitor such as Mitoxantrone significantly reduces DLBCL proliferation further supports the need for the development of USP11-specific inhibitors for targeting DLBCL. Our findings are in contrast with observations from two independent studies. Chen and colleagues reported that USP11 acts as a tumor suppressor by regulating PML protein stability in Notch-induced brain tumor^58^. Studying the regulation of tumor suppressor Hippo cascade, Zhang et al. reported that USP11 stabilizes VGLL4 and may modulate VGLL4/YAP-TEADs regulatory loop^59^. These discrepancies may be due to the difference in cancer types warrants further investigations.

Herein, we demonstrated that FASN inhibition restrains ABC-DLBCL proliferation and that depleting eIF4B or USP11 inhibits DLBCL growth that is independent of COO. This raises an important question, what are the molecular mechanism(s) insulating GC-DLBCL from the deleterious effects of FASN inhibition and not depleting USP11/eIF4B? Multiple signaling cascade including PI3K-Akt as well as ß-catenin is dampened by FASN inhibition^23^. Our data shows that inhibiting Wnt signaling has minimal impact on eIF4B/USP11 axis in our system. Interestingly, we find that the PI3K-S6Kinase pathway seems to have a direct impact on USP11 recruitment on the TIC as well as its interaction with eIF4B. Studying the evolutionary development of USP11, Vlasschaert et al. predicted USP11 as a potential substrate for AGC-kinase family^60^. Indeed, USP11 was noted to be phosphorylated by S6Kinase that increases its interaction with eIF4B and subsequently enhanced cancer-promoting gene translation. Multiple pre-clinical and clinical studies have provided strong support for targeting PI3K signaling in controlling B-cell malignancies^61,62^. Furthermore, Paul et al. proposed using dual PI3K inhibitors in controlling ABC-DLBCL proliferation^63^. Interestingly, expression of PTEN, a major negative regulator of PI3K signaling, was lost in majority of GC-DLBCL^64^. Moreover, aberrant activity of mTOR pathway has emerged as a major marker for aggressive disease and poor prognosis in NHL^65,66^. In line with these clinical observations, S6kinase regulating USP11/eIF4B oncogenic-translational capabilities provide additional important mechanistic insights into this heterogeneous disease. In summary, we demonstrated that enhanced FASN activity induced PI3K-S6Kinase signaling promotes USP11 recruitment to the TIC. This stabilizes eIF4B inducing oncogenic translation. Our findings support the development of targeting strategies that include novel combinatorial approaches with PI3K interruption and potential USP11 and/or eIF4B inhibitors that may have broad utility in controlling DLBCL and related malignancies.

## Methods

### Cell lines, culture, and generation of stable cell lines

All cells were obtained from ATCC and maintained at 37 °C with 5% CO_2_, except HLY-1 cells which was a gracious gift from Dr. Ari Melnick (Weill Cornell, NY). HEK293T/17 were cultured in DMEM supplemented with 10% fetal bovine serum (FBS, Atlanta Biologicals). All other cells were grown in RPMI-1640, 10% FBS. Exponentially growing cells were treated with selected inhibitors and maintained at 37 °C, harvested at indicated time points for further analysis. Stable cells were generated by lentivirus infection, selected and maintained with puromycin (1 mg/mL). Post selection, cells were cultured in RPMI containing 10% FBS in the absence of puromycin. All the chemical inhibitors were purchased from Sigma.

### Plasmids, lentiviral production, and infection

eIF4B cDNA was amplified from human B cell cDNA and cloned into S-protein/FLAG/streptavidinn binding protein (SFB)-triple tagged destination vector (generous gift from Dr. MS Reddy (CDFD, TG, India) using Gateway cloning system (Invitrogen). USP11 (wt and catalytically inactive (CS)) and USP7 expression constructs were a generous gift from Goedele Maertens & Gordon Peters (Addgene plasmid # 46749). USP11^S453A/D^ mutants were generated by using site directed mutagenesis kit (Agilent technologies, Palo Alto, CA, USA) according to manufacturer’s recommendations. Expression constructs of USP11 (wt and all mutants) were amplified and cloned into lentiviral-vector system CD513B (System Biosciences, CA). Lentiviral constructs for knockdown of eIF4B, USP11, USP7, and FASN were purchased from Sigma. For lentiviral packaging, HEK293T/17 cells were seeded at 50% confluence and transfected using packing plasmids psPAX2 and pMD2.G (genereous gift from Dr. Didier Trono; Addgene # 12260 and $12259). Lentiviral particles were concentrated using Amicon Ultra-15 100 kDA centrifugal filters (EMD Millipore) per manufacturer’s protocol. DLBCLs were treated with polybrene (4 mg/mL, American Bioanalytical) and centrifuged (35 min, 300×*g*, 25 °C). Cell pellets were resuspended in virus-containing medium for 48 h, then selected and maintained with puromycin (1 mg/mL).

### B-cell sorting

Human B-cells from otherwise healthy motor vehicle collision patients were provided by the R. Adams Cowley Shock Trauma Center and UMGCC Pathology Biorepository and Research Core in accordance with the guidelines of the University of Maryland Medical School Institutional Review Board and conform to the Declaration of Helsinki. Primary human tissue was minced on ice and mononuclear cells isolated using density centrifugation (lymphocyte separation medium). B-cells were isolated by magnetic bead separation (B-CLL cell isolation kit, human, Miltenyi Biotec, as per manufacturer protocol) before further use.

### Polysomal fractionation

Polysomal fractionation was performed as reported earlier^67^. Briefly 10 million cells were lysed in polysomal lysis buffer and fractionated using a linear sucrose gradient (10–50%). Fractions were separated on 4–12% SDS page and probed for the indicated antibodies.

### Puromycin incorporation assay (SUnSET assay)

SUnSET assay was performed as per manufacturer’s recommendations (Kerafast). Cells were incubated with puromycin (normal cells (non-infected): 1 µg/mL, infected cells: 3 µg/mL) for 30 min. Post incubation, cells were washed with ice cold PBS and lysed using RIPA lysis buffer. Equal quantity of protein lysates was separated on SDS-PAGE and probed with anti-puromycin antibody. Signals were normalized with probing GAPDH (loading control).

### Western blotting

Cells were lysed in RIPA buffer (50 mM TRIS pH 7.5, 150 mM NaCl, 0.1% SDS, 0.5% sodium deoxycholate, 1% triton X-100, 1 mM mEDTA, and 1 mM EGTA, 1 mM sodium orthovanadate, 1 mM sodium fluoride, 1× protease inhibitor (Sigma-Aldrich), phosphatase inhibitor cocktails #2 and #3 (Sigma-Aldrich), and 1 mM PMSF). Cells lysate were quantified using Bradford reagents and equal amount of protein was seperated on a Nupage 4–12% Bis-Tris gradient gel (Life Technologies) and probed with the following antibodies: eIF4B (SCBT, sc-376062, 1:1000), USP11 (Abcam, ab109232, 1:300 or SCBT, sc-365528, 1: 1000), FASN (Abcam, ab22759, 1:1000), USP7 (Abcam, ab4080 or SCBT, sc-133204, 1:1000), eIF4A (sc-377315 or sc-14211, 1:1000), eIF4E (SCBT, sc-9976, 1:1000), cMYC (SCBT, sc-373712 or SC-764, 1:1000), Bcl2 (CST # 2870 or SCBT, sc-509, 1:1000), Bcl6 (CST, #5650, 1:1000), PARP-1 (SCBT, sc-7150, 1:1000), XIAP (CST # 2042 or #2045, 1:1000), p-70S6kinase (T421, S424) (CST, #9204, 1:1000), Total 70S6kinase (CST, #9202, 1:1000), Actin (SCBT, sc-1615, 1:1000), GAPDH (Abcam, ab8245, 1:10,000), Flag (Sigma, F3165, 1:10,000), HA (BioLegend, MMS-101P, 1:10,000), Ubiquitin (SCBT, sc-8017, 1:2000), His (SCBT, sc-803, 1:5000), SOCS1 (CST #3950, 1:1000), A20 (CST #4625, 1:1000), BLIMP1 (SCBT, sc-130917, 1:1000), DUSP4 (SCBT, sc-135991, 1:1000), Vinculin (Sigma, V9131, 1:10,000). Densitometry analyses were performed using Image Studio (Licor Biosciences) and presented as ratio of target band signal intensity to GAPDH/Actin/Vinculin band signal intensity. Full immunoblots of the main figure are presented in Supplementary Figure 38.

### Immunoprecipitation and m^7^GTP pulldown

For immunoprecipitation, 500 µg of protein lysates were pre-cleared with protein A sepharose beads (50 µL/500 µL of sample) for 1 h. Pre-cleared lysates were incubated with either primary antibody–beads complex (2 µg eIF4B or USP11 (SCBT)) or beads (50 µL Streptavidin beads or flag beads) for 2 h. Lysate beads mixture was washed four times with lysis buffer and diluted in 2× loading gel dye. Samples were boiled and separated on 4–12% SDS page and probed for the indicated antibodies. For analyzing the translation initiation complex, m^7^GTP analog beads (50 µL) were incubated with/without m^7^GTP salt (competitive salt) with pre-cleared lysates for 2 h. Post incubation, beads–protien complex was washed three times with lysis buffer and boiled with 2× loading dye followed by separation on 4–12% SDS PAGE.

### In vitro S6kinase assay

Purified USP11 or Flag tagged USP11 (wt or Mutant) were incubated with purified catalytically active S6kinase in presence of 1× kinase buffer (25 mM HEPES, pH 7.4, 5 mM β-glycerolphosphate, 3.75 mM EGTA, 30 mM MgCl2, 0.15 mM Na3VO4, 1.5 mM DTT, 20 µM ATP) at 30 °C for 30 min. The assay was terminated by adding SDS loading dye and boiled at >90 °C for 5 min. Samples were separated on 4–12% SDS page and probed with pSer antibody (Abcam, ab9332, 1:1000). The blots were stripped and probed for S6kinase and USP11, respectively.

### Cellular phosphorylation assay

293T cells were transfected with USP11 (wt) or with mutant and cultured in presence of 20% FBS to increase basal signaling. Cells transfected with USP11 alone (Fig. 8j) were treated for 3 h with S6kinase inhibitor (1 µM). Cells were subsequently washed, lysed, and USP11 was precipitated using specific antibody or beads (flag, see protocol of IP). Phospho signal of USP11 was quantified by pSer antibodies.

### USP11 deubiquitinase assay

Flag tagged USP11 was enriched from 293T lysates using flag beads. Polyubiquitinated eIF4B was purified: SFB-eIF4B transfected 293T cells were treated with C75 for 6 h. Post treatment, cells were lysed and eIF4B (polyubiqutinated) was enriched by streptavidin beads. eIF4B enriched samples were incubated with USP11 beads in 1 × kinase buffer (50 mM Tris pH7.6, 50 mM NaCl, 1 mM EDTA, 5 mM DTT) for 1 h. Post incubation, beads were washed with ice-cold PBS and ubiquitin levels of eIF4B were assessed by probing with ubiquitin antibody. For cellular deubiquitination assay, 293T cells were transfected with SFB-eIF4B in presence or absence of USP11. Twenty-four hours post transfection, cells were treated with C75 for 6 h and eIF4B was enriched using streptavidin beads. Ubiquitin levels were detected using specific antibodies.

### Immunohistochemistry (IHC)

IHC was performed as discussed earlier^68^. Briefly TMA slides (Biomax, # LY800a) were dewaxed, washed once in absolute ethanol, and incubated in 3.3% hydrogen peroxide in methyl alcohol for 40 min in the dark at room temperature in order to inactivate endogenous peroxidase. The sections were hydrated in graded alcohol followed by three washes in PBS for 5 min. Sections were incubated in blocking solution containing 5% BSA and 2% horse serum for 2 h, followed by overnight incubation at 4 °C with eIF4B, FASN, and USP11 antibody. Sections were then washed three times with PBS, incubated in a biotinylated goat anti-rabbit IgG (diluted 1:200) for 1 h, washed again in PBS, and incubated in Vectastain ABC kit (Vector Labs Inc., Burlingame, CA, USA) for 1 h. After three washes in PBS, immunolabeling was revealed with 0.05% diaminobenzidine (DAB). Sections were then dehydrated in ethanol, cleared in xylene, and cover slipped with mounting medium. Based on the staining intensity, samples were scaled between 0–4 as discussed earlier.

### Xenograft assay

The Institutional Animal Care and Use Committee of the University of Maryland approved all procedures involving animals. Female SCID Beige mice were housed in a pathogen-free environment under controlled conditions of light and humidity and received food and water ad libitum. Stable HLY-1 cells (1 million) were resuspended in 1× PBS and mixed with equal volume of matrigel. The complex was injected in subcutaneously in left and right dorsal flank in 5–7-weeks-old mice. When the tumor reached 200 mm^3^, animals were sacrificed and the tumor was preserved for further analysis.

### Assessment of viable cells

Exponentially growing cells were seeded in equal density (1 million) (either for treatment or stable cells). Post 12 h of seeding, cells were collected till the indicated time points and stained with trypan blue. The number of non-viable cells and viable cells were counted depending upon intake of trypan blue in hemocytometer.

### Clonogenic methylcellulose assays

DLBCL cells (25–50 × 10^3^) were plated in methylcellulose (RnD). Colonies were visualized and counted after 15 days.

### Statistics

Values were expressed as mean ± S.D. For comparison between two groups, the unpaired Student’s *t*-test was used. One-way ANOVA followed by Bonferroeni’s post-hoc analysis was used to compare more than two groups. Wilcoxon signed-rank test was used to compare the data sets between normal and DLBCL samples, *p* < 0.05 was considered as significant. Results represent means ± standard deviation (SD) of minimum three independent experiments.

### Data availability

All the supporting data for this study are available from the corresponding author on reasonable request.

## Electronic supplementary material

Supplementary Information
