# Supplementary material for: Fatty Acid Synthase induced S6Kinase facilitates USP11-eIF4B complex formation for sustained oncogenic translation in DLBCL
Source: Nat Commun. 2018 Feb 26;9:829. doi: 10.1038/s41467-018-03028-y (PMC5827760; doi:10.1038/s41467-018-03028-y)
Supplement: Supplementary file 1 — Supplementary Information [file 41467_2018_3028_MOESM1_ESM.pdf]

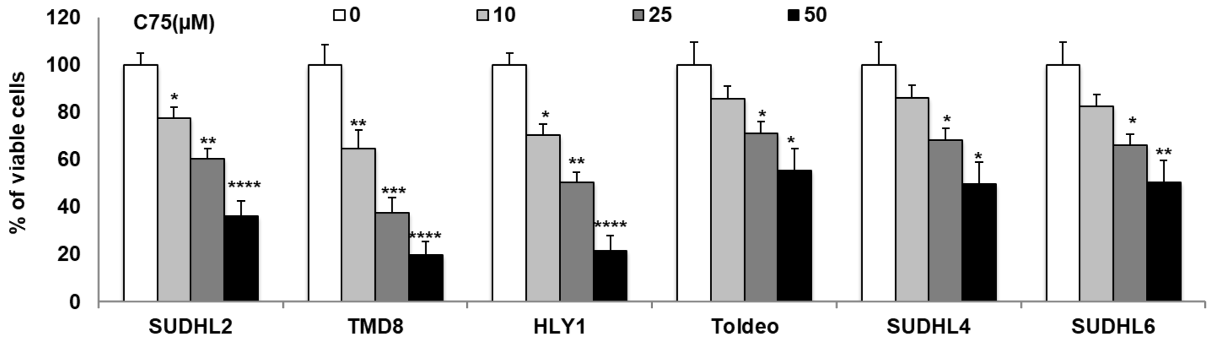

Supplementary figure 1: FASN inhibition depletes ABC-DLBCL growth

Indicated cells were treated with increasing concentration of C75 for 14 h. Post treatment cells were stained with trypan blue and counted in hemocytometer. Values were normalized with vehicle control, DMSO (0.1%) and expressed as mean±SD (n=5). Statistical analysis was performed using Student's *t*-test (Unpaired two tailed) *$p<0.05$, **p, 0.01, ***$p<0.005$, ****$p<0.001$ vs. DMSO treated corresponding cells.

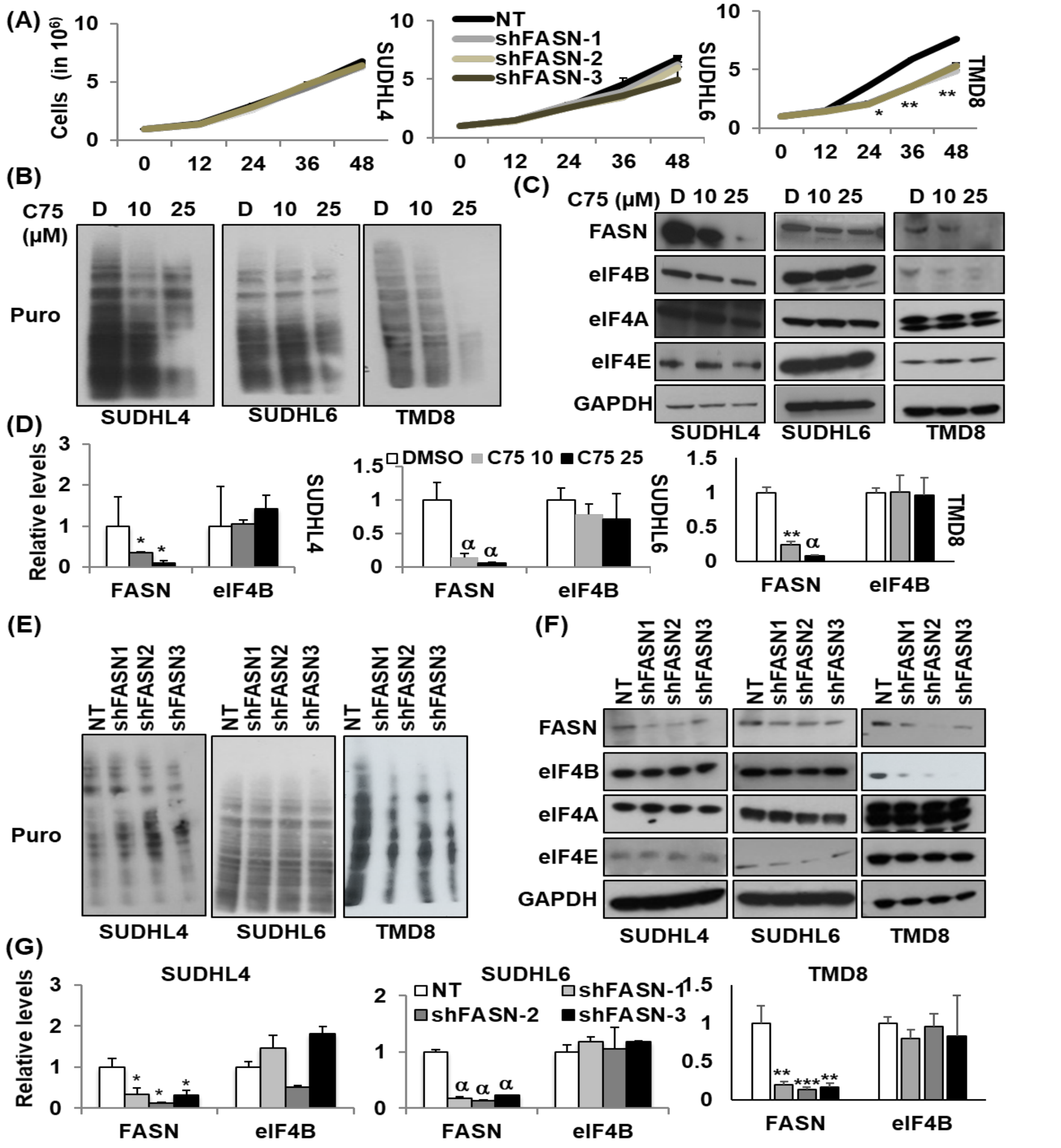

Supplementary Figure 2: FASN inhibition has no effect on mRNA translation in GC DLBCL

Indicated cells were infected with shRNA against FASN or NT (scrambled) and selected on puromycin. Post selection 1 million were seeded in 6 well plates and counted using trypan blue staining every 12h for indicated time points. Values are shown as mean±SD for n=3. Statistical analysis was performed using Student's *t*-test (Unpaired two tailed) **p*<0.05, ***p*,0.01, ****p*<0.005 vs NT infected corresponding cells. (B & C) Indicated cells were treated with defined concentration of C75 for 14 h followed by 30 min with Puromycin treatment (1µg/mL) and lysed. Cell lysates were subjected to immunoblotting with anti-Puro antibody (SUnSET assay) (B) or

indicated antibodies (C). GAPDH was used as loading control. (D) qRT-PCR analysis of FASN and eIF4B expression in indicated cells treated with defined concentration of C75. Results were normalized with DMSO treated corresponding cells and expressed as mean±SD (n=3). Statistical analysis was performed using Student's *t*-test (Unpaired two tailed) *$p<0.05$, $^{\alpha}p<0.001$vs DMSO treated corresponding cells. (E-F) Indicated cells were infected by shRNA against FASN and cultured in puromycin (0.5-1μg/mL) for stable cells generation. Post selection, cells were exposed for 30 min with Puromycin treatment (3μg/mL) and lysed. Cell lysates were subjected to immunoblotting with anti-Puro antibody (SUnSET assay) (E) or indicated antibodies (F). GAPDH was used as loading control. (G) qRT-PCR analysis of FASN and eIF4B expression in indicated stable cells infected with shRNA against FASN or NT (scrambled). Results were normalized with NT infected corresponding cells and expressed as mean±SD (n=3). Statistical analysis was performed using Student's *t*-test (Unpaired two tailed) *$p<0.05$, $^{\alpha}p<0.001$ vs NT infected corresponding cells.

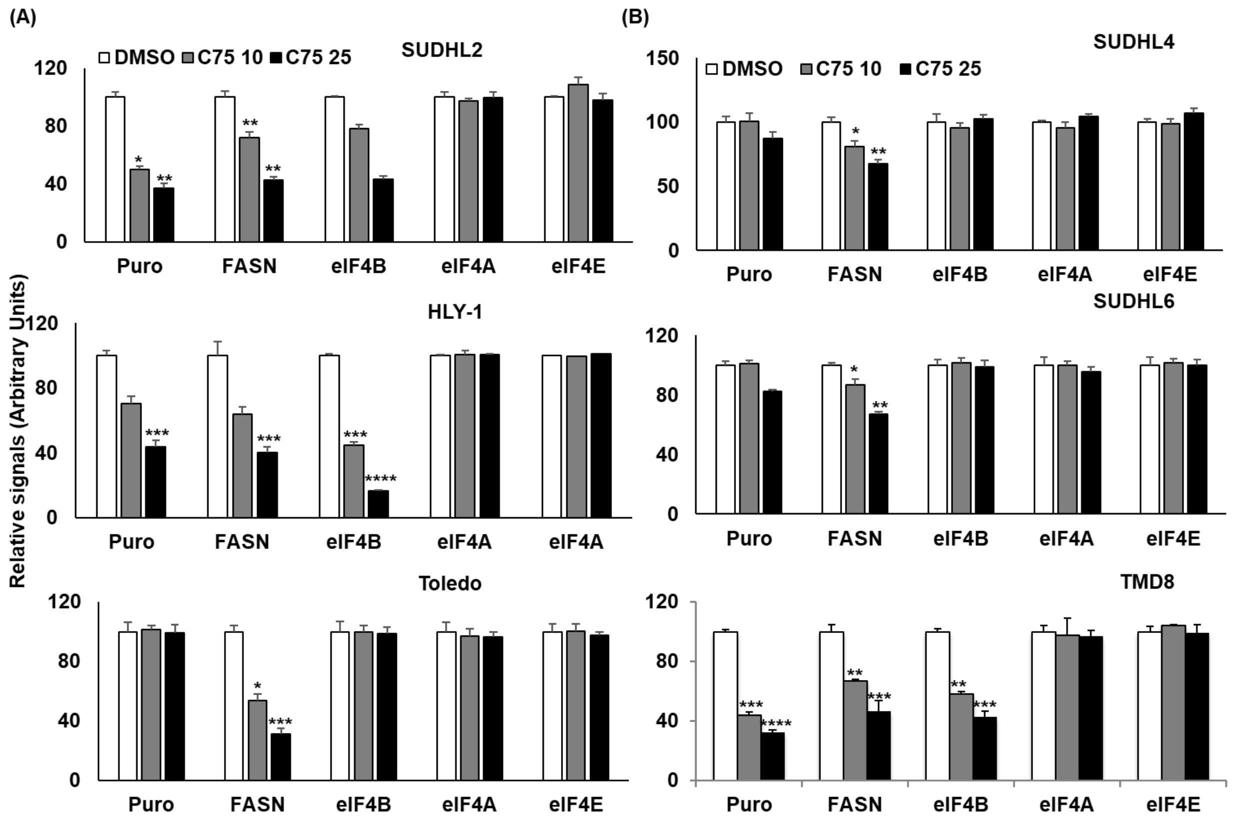

Supplementary Figure 3:

(A) Densitometric quantification of the immunoblots in Figure 1B and 1C. Values were normalized with vehicle control, DMSO (0.1%) and expressed as mean±SD (n=5). Statistical analysis was performed using Student's *t*-test (Unpaired two tailed) *p<0.05, **p,0.01, ***p<0.005, ****p<0.001 vs DMSO treated corresponding cells. (B) Densitometric quantification of the immunoblots in Figure Supplementary 2B and 2C. Values were normalized with vehicle control, DMSO (0.1%) and expressed as mean±SD (n=5). Statistical analysis was performed using Student's *t*-test (Unpaired two tailed) *p<0.05, **p,0.01, ***p<0.005 vs DMSO treated corresponding cells.

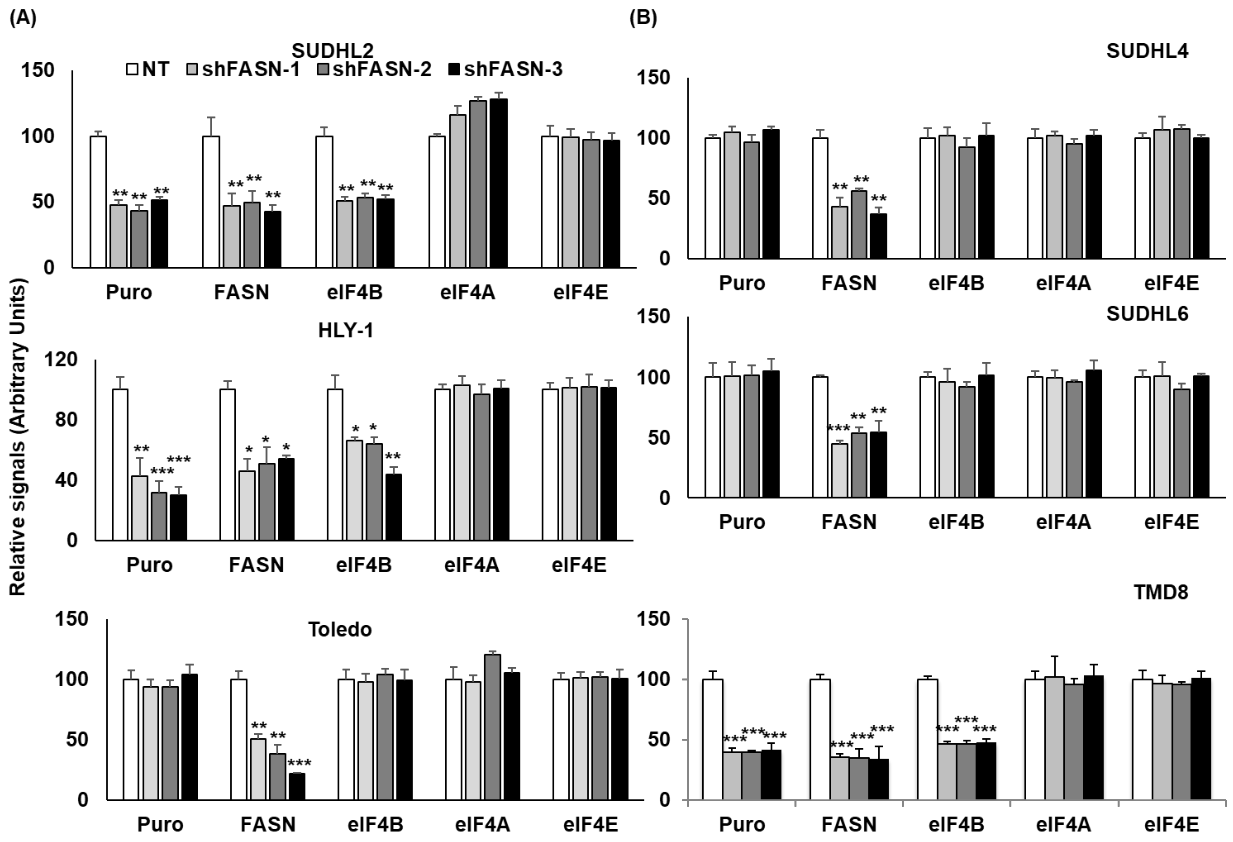

Supplementary Figure 4:

(A & B) Densitometric quantification of the immunoblots in Figure 1E & 1F and Supplementary Figure 2E and 2F (B). Values were normalized with corresponding GAPDH and neutralized with DMSO treated corresponding cells, which was set to 100. Values were represented as mean±SD for n=3. Statistical analysis was performed using Student's *t*-test (Unpaired two tailed) *p<0.05, **p, 0.01, ***p<0.005 vs NT infected corresponding cells.

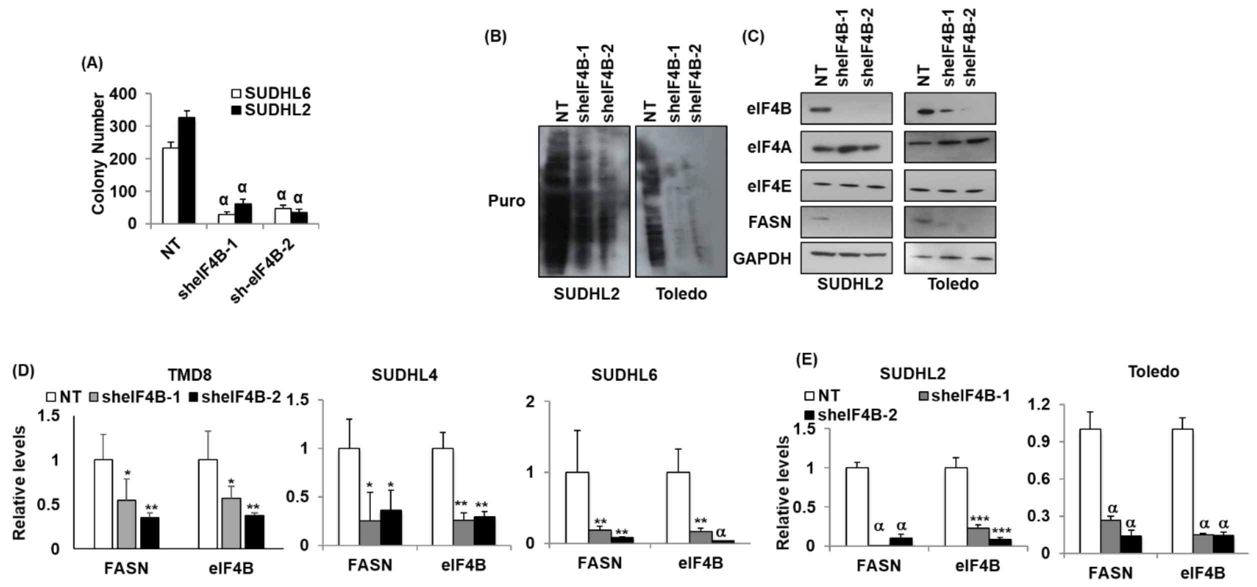

Supplementary Figure 5: eIF4B is required for DLBCL proliferation

(A) Total number of colonies grown in eIF4B depleted SUDHL2 and SUDHL6 in methylcellulose culture. Colony counts were performed on the 15th day of methylcellulose culture. $^{\alpha}p$<0.001 vs corresponding NT infected control cells. (B and C) Indicated cells were infected by shRNA against eIF4B and cultured in puromycin (0.5-1µg/mL) for stable cells generation. Post selection, cells were exposed for 30 min with Puromycin treatment (3µg/mL) and lysed. Cell lysates were subjected to immunoblotting with anti-Puro antibody (SUnSET assay) (B) or indicated antibodies (C). (D & E) qRT-PCR analysis of FASN and eIF4B expression in indicated stable cells infected with shRNA against eIF4B or NT (scrambled). Results were normalized with NT infected corresponding controls and expressed as mean±SD (n=3). **p*<0.05, ***p*,0.01, ****p*<0.005, $^{\alpha}p$<0.001 vs NT infected corresponding cells. GAPDH was used as reference gene.

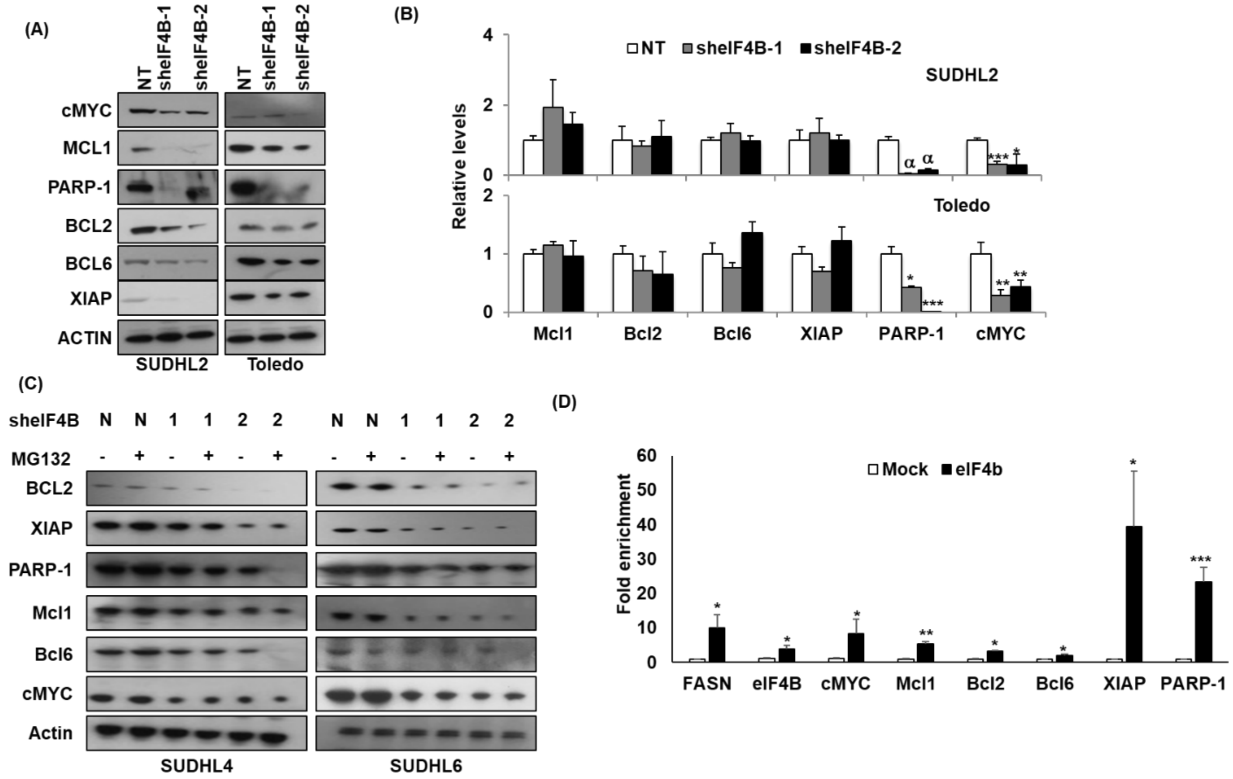

Supplementary Figure 6: eIF4B regulates translation of oncogenes

(A) Indicated eIF4B depleted stable cells were lysed and lysates were probed for the defined antibodies. (B) qRT-PCR analysis for expression of defined genes in indicated stable cells infected with shRNA against eIF4B or NT (scrambled). Results are expressed as mean±SD (n=3). Statistical analysis was performed using Student's *t*-test (Unpaired two tailed) *$p<0.05$, **$p<0.01$, ***$p<0.005$ $^{\alpha}p<0.001$ vs NT infected corresponding cells. (C) Exponentially growing eIF4B depleted stable SUDHL4 and SUDHL6 cells were cultured with MG132 (10μM) for 2 h and cell lysates were probed for the defined antibodies. N: scrambled; 1: sheIF4B-1; 2: sheIF4B-2 (D) Total cell lysates from SUDHL2 were subjected to immunoprecipitation with eIF4B antibodies, followed by RNA isolation and qRT-PCR to detect the enrichment of the defined genes. Results were normalized total RNA from cell lysates and expressed relative percentage to mock sample enriched RNA. Values are expressed as mean±SD and statistical analysis was performed using Students t-test, *$p<0.005$, **$p<0.01$, $^{***}p<0.005$ vs corresponding mock samples.

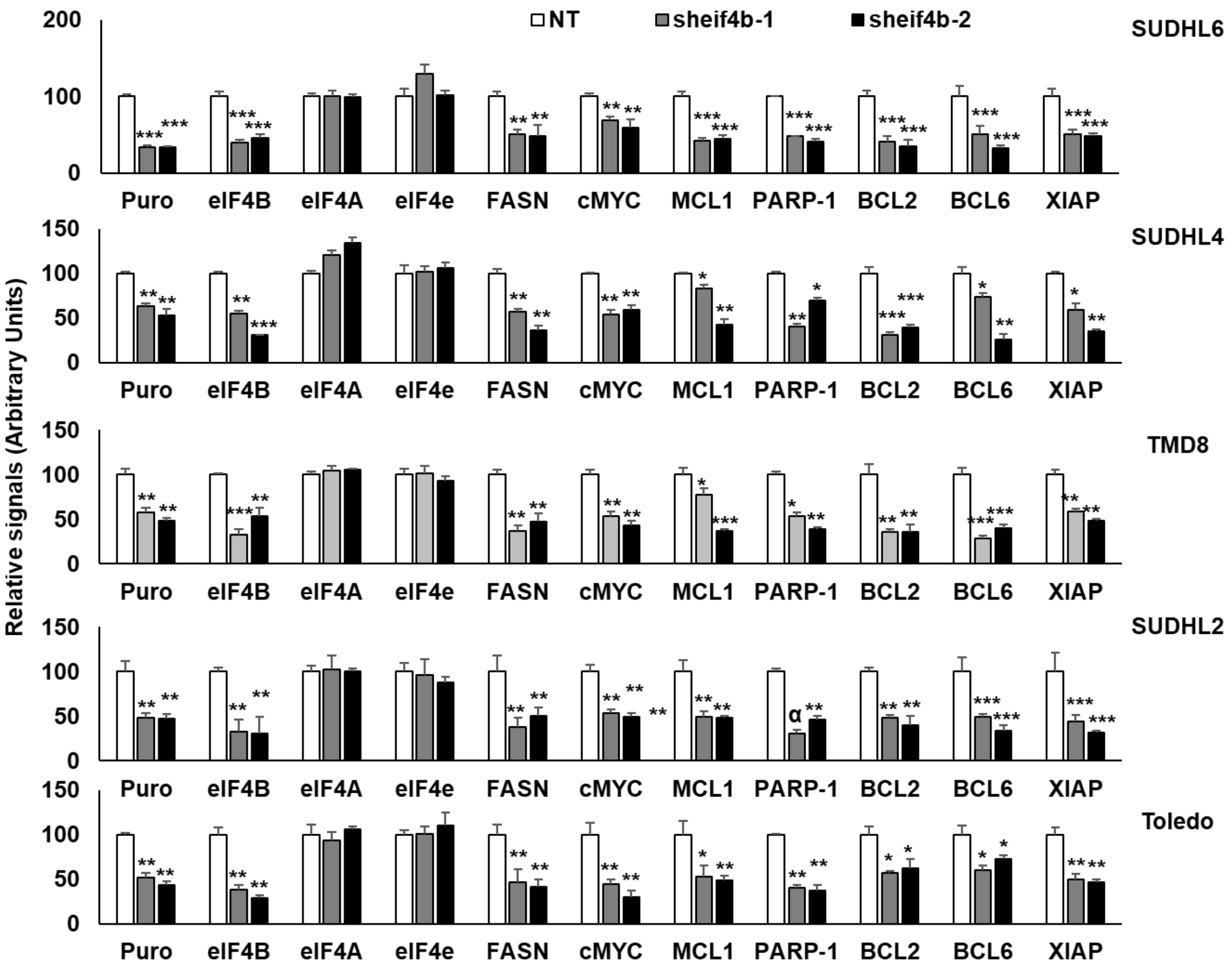

Supplementary Figure 7:

Densitometric quantification of the immunoblots in Figure 2B, 2C & 3A and Supplementary figure 5B, 5C & 6A. Values were normalized with corresponding NT infected corresponding cells and were represented as mean±SD for n=3. Statistical analysis was performed using Student's *t*-test (Unpaired two tailed) **p*<0.05, ***p*,0.01, ****p*<0.005, ◦*p*<0.001 vs NT infected corresponding cells.

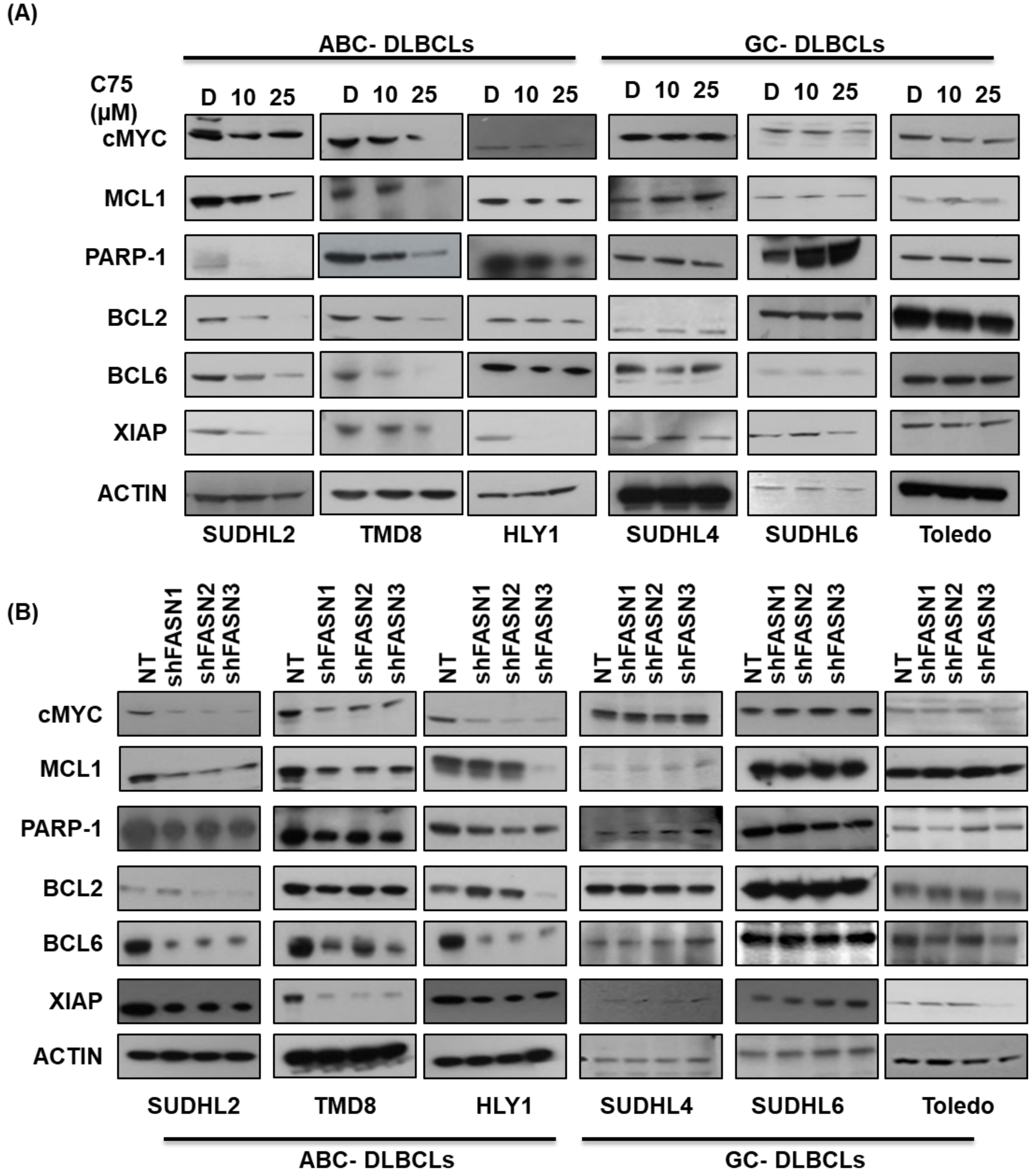

Supplementary Figure 8: FASN inhibition decreases eIF4B-sensitive oncogenes.

(A & B) Indicated cells were either treated with increasing concentration of C75 (A) or transduced different shRNA against FASN (B) and probed for indicated antibodies. Actin was used as loading control.

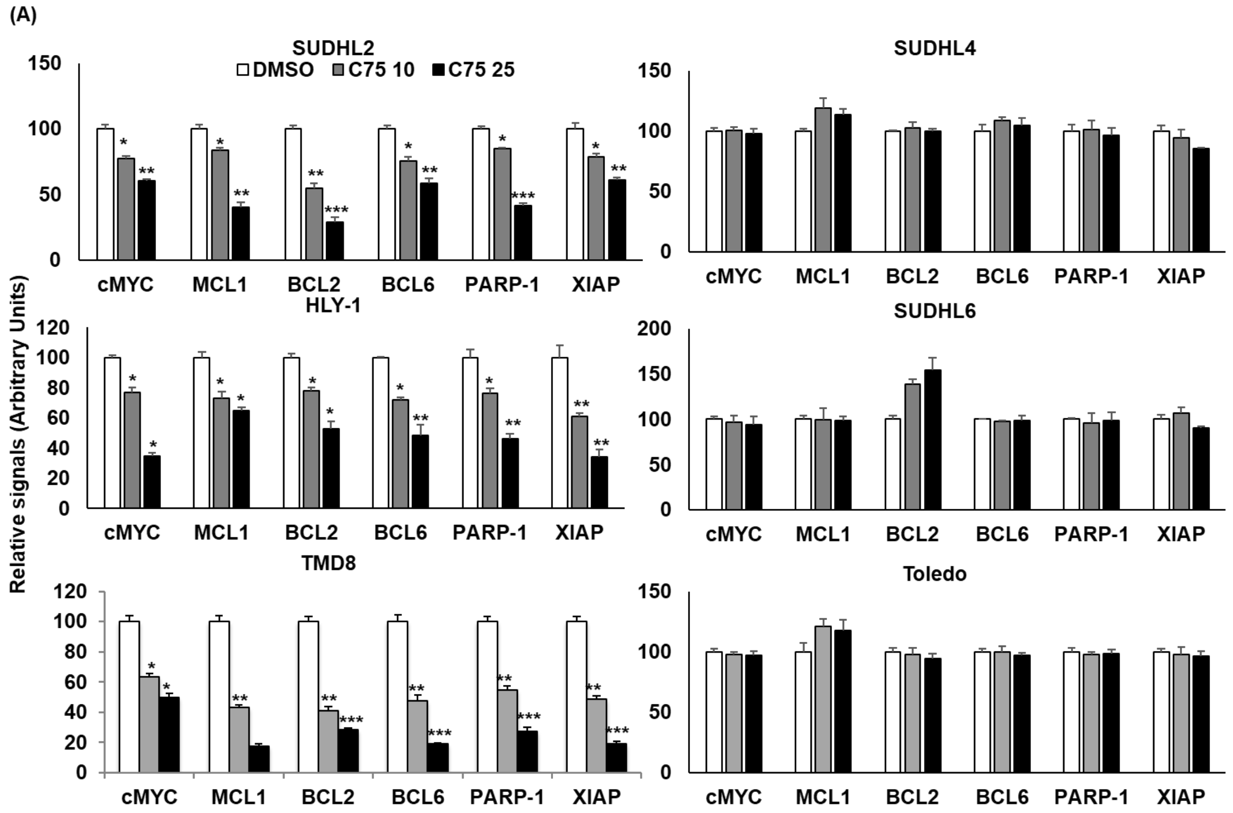

Supplementary Figure 9:

Densitometric quantification of the immunoblots in Supplementary Figure 8A. Values were normalized with vehicle control, DMSO (0.1%) and expressed as mean±SD (n=5). Statistical analysis was performed using Students *t*-test (Unpaired two tailed) *$p<0.05$, **p,0.01, ***$p<0.005$ vs DMSO treated corresponding cells.

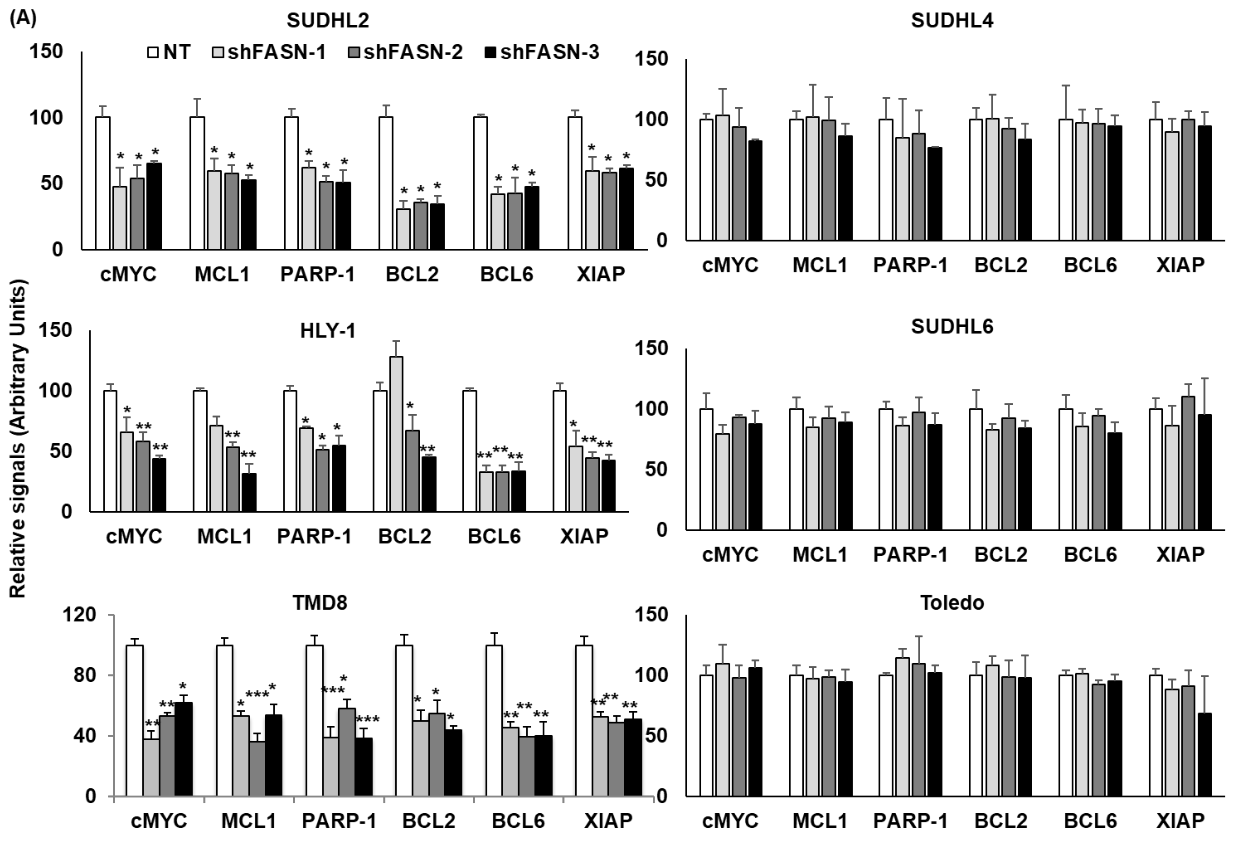

Supplementary Figure 10:

Densitometric quantification of the immunoblots in Supplementary Figure 9B. Values were normalized with corresponding NT infected corresponding cells and were represented as mean±SD for n=3. Statistical analysis was performed using Student's *t*-test (Unpaired two tailed) **p*<0.05, ***p*,0.01, ****p*<0.005 vs NT infected corresponding cells.

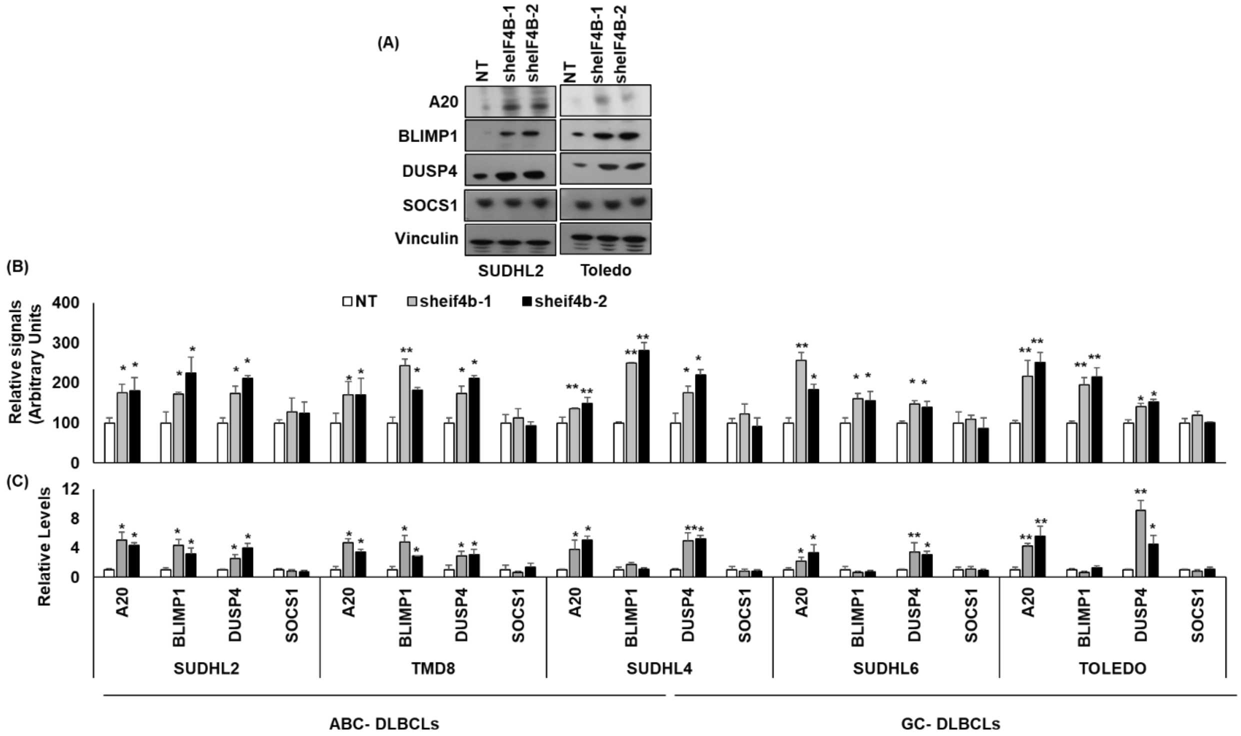

Supplementary Figure 11: eIF4B depletion enhances tumor suppressor genes expression

(A) Indicated eIF4B depleted stable cells were lysed and lysates were probed for the defined antibodies. (B) Densitometric quantification of the immunoblots in Supplementary Figure 11A. Values were normalized with corresponding NT infected corresponding cells and were represented as mean±SD for n=3. Statistical analysis was performed using Student's *t*-test (Unpaired two tailed) *$p<0.05$, **p,0.01, vs NT infected corresponding cells. (C) qRT-PCR analysis for expression of defined genes in indicated stable cells infected with shRNA against eIF4B or NT (scrambled). Results are expressed as mean±SD (n=3). Statistical analysis was performed using Student's *t*-test (Unpaired two tailed) *$p<0.05$, **p,0.01 vs NT infected corresponding cells.

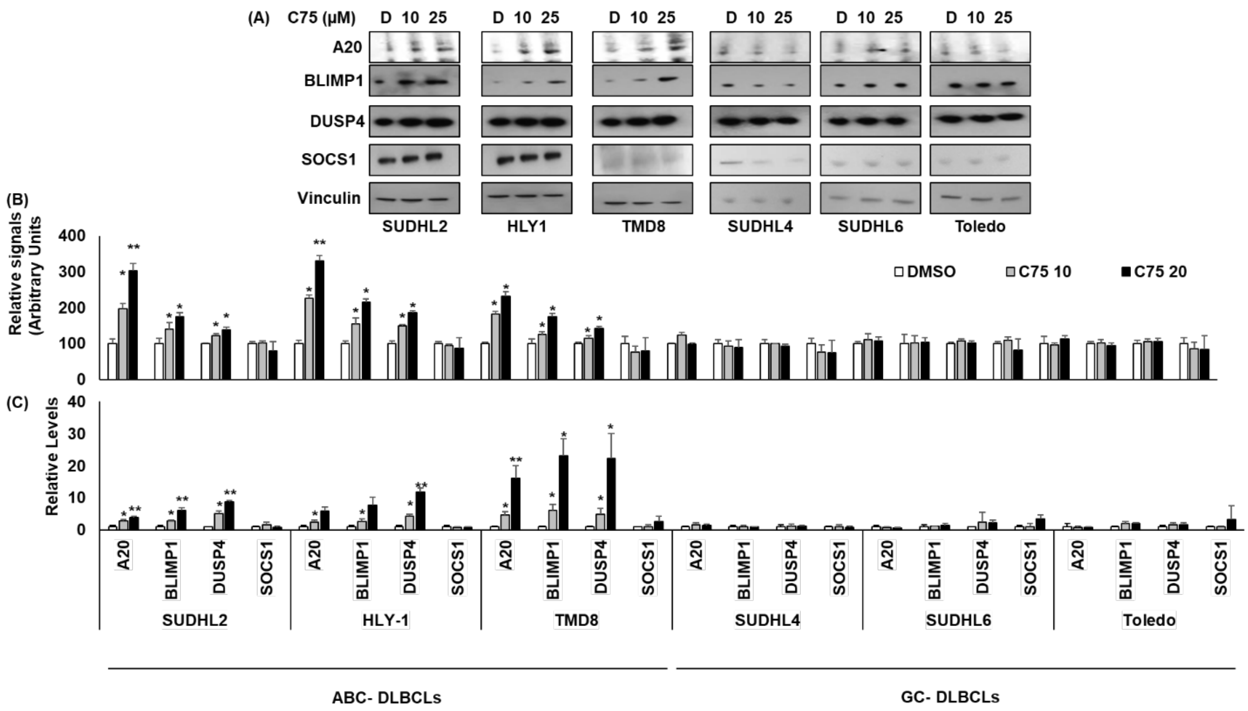

Supplementary Figure 12: FASN inhibition enhances tumor suppressor genes expression.

(A) Indicated cells were treated with C75 in increasing concentrations for 16h followed by lysis and lysates were probed for the defined antibodies. (B) Densitometric quantification of the immunoblots in Supplementary Figure 12A. Values were normalized with corresponding DMSO treated cells and were represented as mean±SD for n=3. Statistical analysis was performed using Student's *t*-test (Unpaired two tailed) **p*<0.05, ***p*,0.01, vs DMSO treated corresponding cells. (C) qRT-PCR analysis for expression of defined genes in indicated cells treated with C75. Results are expressed as mean±SD (n=3). Statistical analysis was performed using Student's *t*-test (Unpaired two tailed) **p*<0.05, ***p*,0.01 vs DMSO treated corresponding cells.

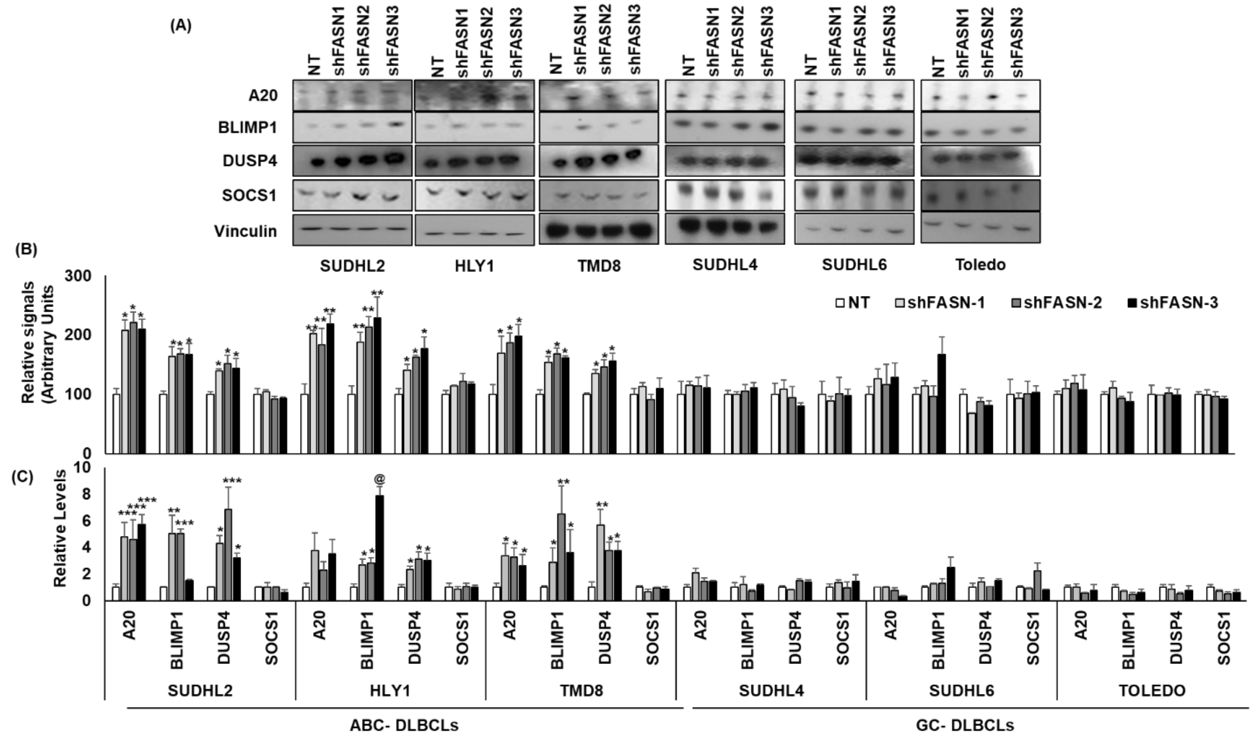

Supplementary Figure 13: FASN depletion enhances tumor suppressor genes expression

(A) Indicated FASN depleted stable cells were lysed and lysates were probed for the defined antibodies. (B) Densitometric quantification of the immunoblots in Supplementary Figure 13A. Values were normalized with corresponding NT infected corresponding cells and were represented as mean±SD for n=3. Statistical analysis was performed using Student's *t*-test (Unpaired two tailed) **p*<0.05, ***p*,0.01, vs NT infected corresponding cells. (C) qRT-PCR analysis for expression of defined genes in indicated stable cells infected with shRNA against FASN or NT (scrambled). Results are expressed as mean±SD (n=3). Statistical analysis was performed using Student's *t*-test (Unpaired two tailed) **p*<0.05, ***p*,0.01, ****p*<0.005, $^{\alpha}p<0.001$ vs NT infected corresponding cells.

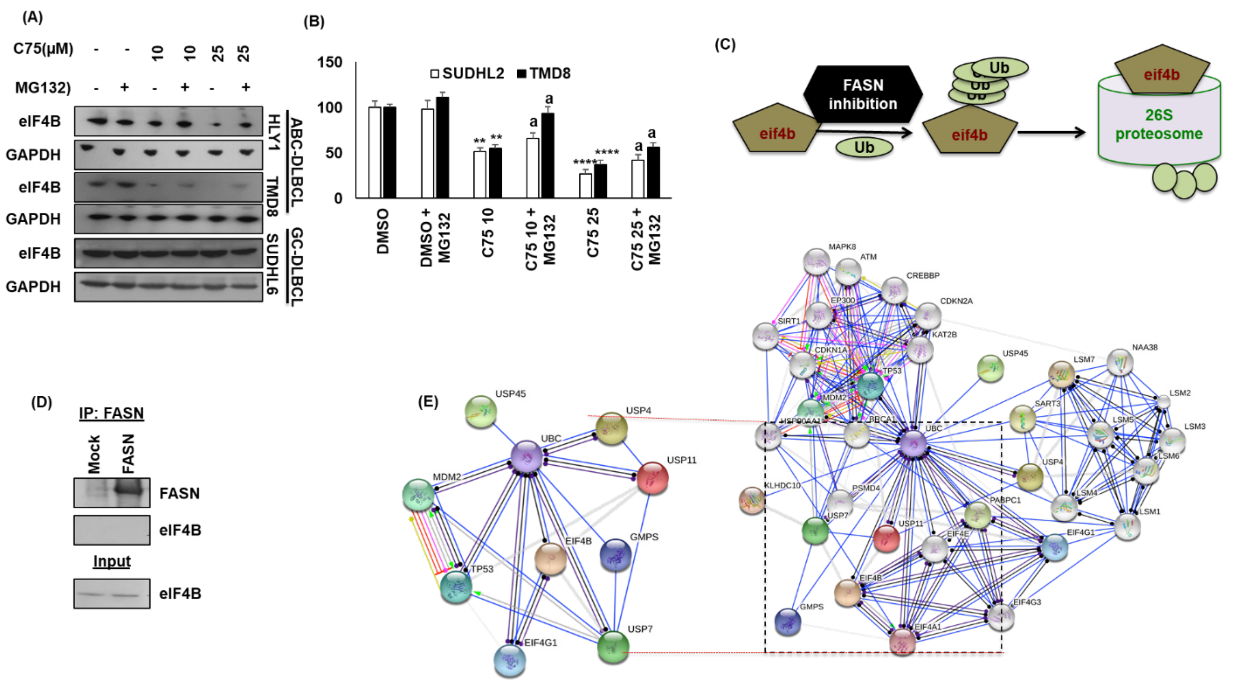

Supplementary Figure 14: FASN inhibition leads to ubiquitin mediated proteosomal degradation of eIF4B.

(A) Indicated cells were treated with C75 (indicated concentration) for 14 h followed by treatment with MG132 (10μM) for 4 hours. Lysates were probed for the indicated antibodies. (B) Densitometric quantification of the immunoblots in Figure 4C and Supplementary Figure 14A for the indicated cells. Values were normalized with corresponding NT infected corresponding cells and were represented as mean±SD for n=6. Statistical analysis was performed using one-way ANOVA followed by Bonferroeni's post hoc test. ***p*<0.01, *****p*<0.001 vs DMSO treated corresponding cells, ^{a}p<0.05, vs C75 treated corresponding cells (w) infected corresponding cells. (C) Cartoon showing FASN inhibition leads to eIF4B proteosomal degradation. (D) Lysates from SUDHL2 was immunoprecipitated with FASN antibody and probed for eIF4B. (E) String analysis of eIF4B interacting partners. Enlarged box represents eIF4B interaction with reported DUBs (string.db.org).

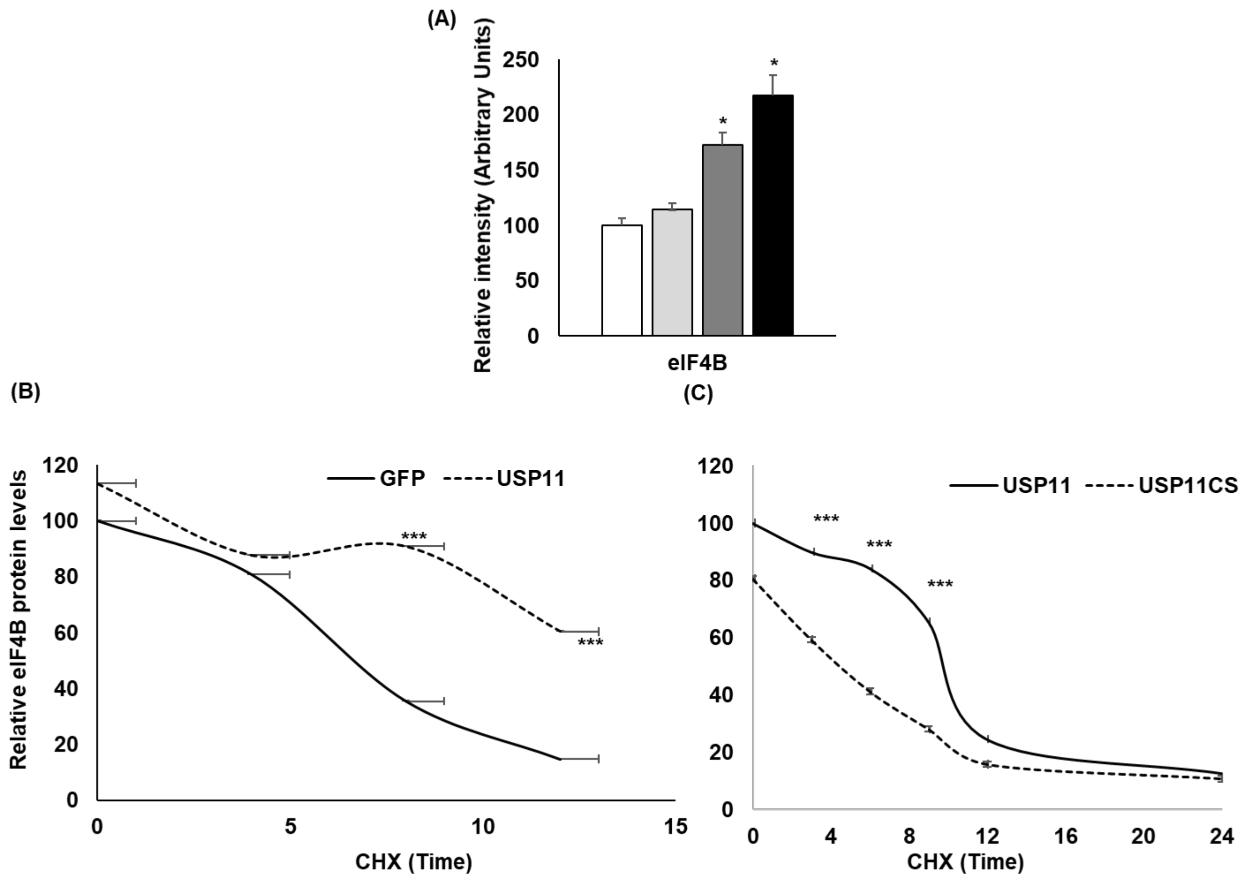

Supplementary Figure 15:

(A) Densitometric quantification of the immunoblots in Figure 4F. Values were normalized with GFP transfected cells and were represented as mean±SD for n=3. Statistical analysis was performed using Student's *t*-test (Unpaired two tailed) **p*<0.05, vs GFP transfected cells. (B & C) Densitometric quantification of Figure 4G and Figure 4H. Values were normalized with GFP (B) or USP11 (C) transfected untreated cells and were represented as mean±SD for n=3. Statistical analysis was performed using Student's *t*-test (Unpaired two tailed) ****p*<0.005, vs NT infected corresponding cells.

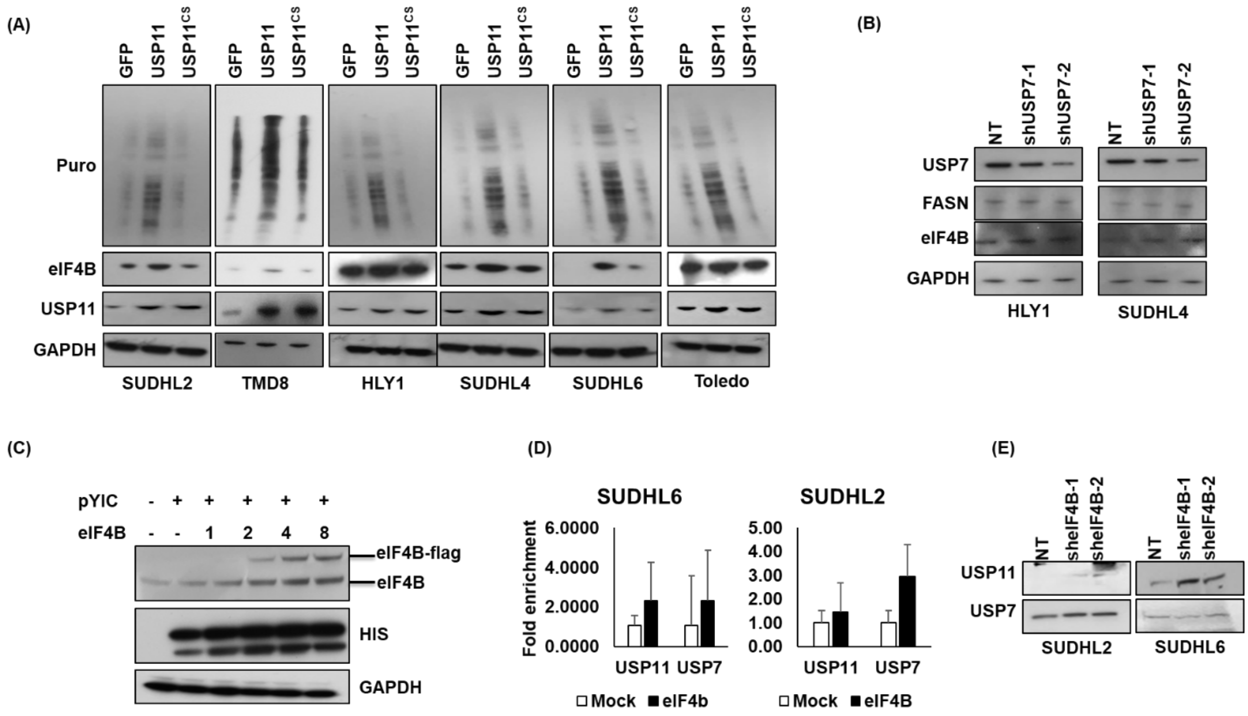

Supplementary Figure 16: USP11 expression promotes translation.

(A) Indicated cells were infected USP11 or its catalytically inactive mutant (USP11^{CS}) and selected on puromycin. Post selection cells were cultured in puromycin (3µg/ml) for 30 min and lysates were probed for indicating antibodies. GFP transduced cells were used as corresponding controls. GAPDH was used as loading control. (B) Indicated USP7 depleted stable cells lysates were probed for indicating antibodies. GAPDH was used as loading control. (C) 293T cells were transfected with pYIC and indicated amount of eIF4B and or GFP. Post transfected, lysates were resolved and probed for indicated antibodies. GAPDH was used loading control. (D) Total cell lysates from indicated cells were subjected to immunoprecipitation with eIF4B antibodies, followed by RNA isolation and qRT-PCR to detect the enrichment of the defined genes. Results were normalized total RNA from cell lysates and expressed relative percentage to mock sample enriched RNA. Values are expressed as mean±SD. (E) Indicated eIF4B depleted stable cells were lysed and lysates were probed for the defined antibodies.

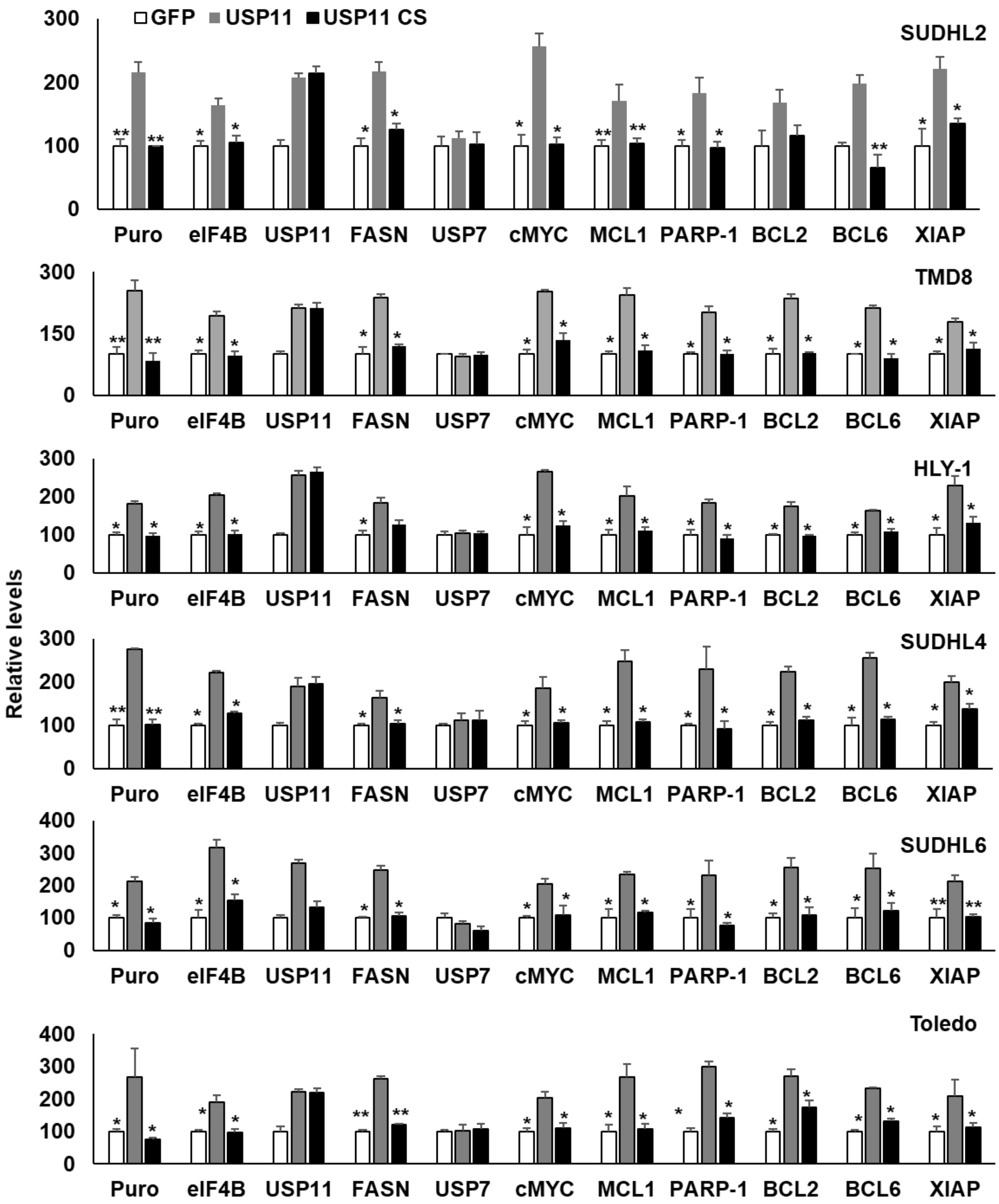

Supplementary Figure 17:

Densitometric quantification of the immunoblots in Supplementary Figure 16A & 23. Values were normalized with corresponding GFP infected corresponding cells and were represented as mean±SD for n=3. Statistical analysis was performed using Student's *t*-test (Unpaired two tailed) **p*<0.05, ***p*,0.01 vs USP11 infected corresponding cells.

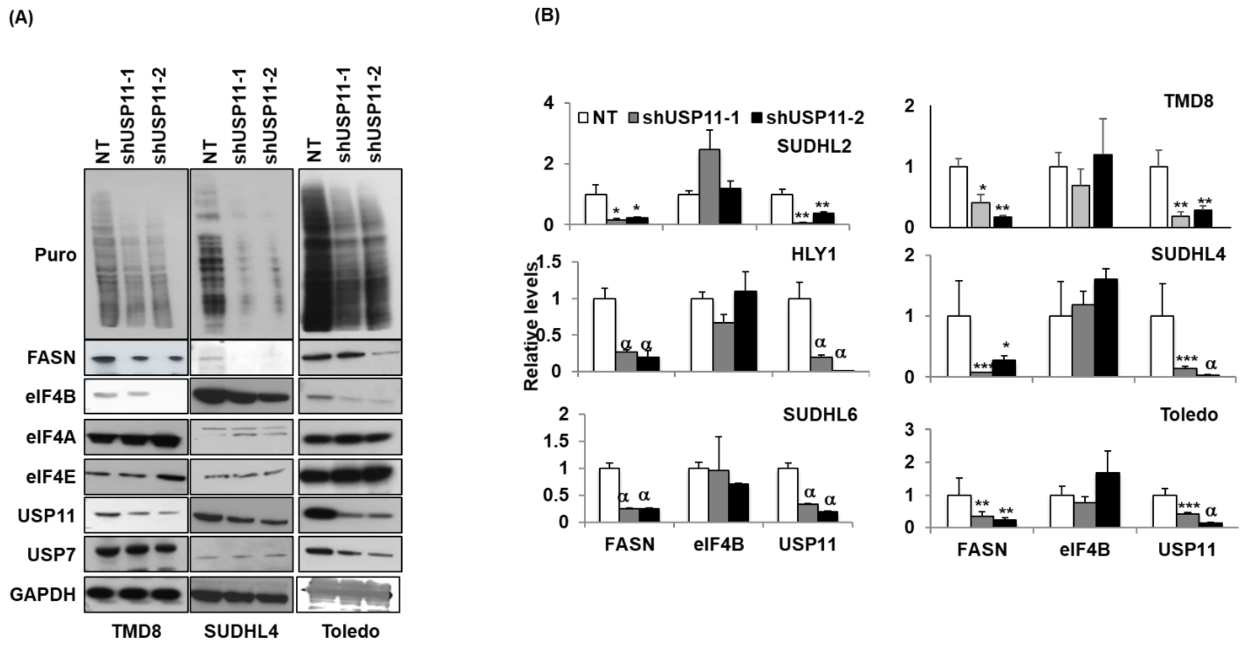

Supplementary Figure 18: USP11 directly regulates translation

(A) Indicated USP11 depleted stable cells were cultured in presence of puromycin (3μg/ml) for 30 min and lysates were probed for indicating antibodies. GAPDH was used as loading control. (B) qRT-PCR analysis for expression of defined genes in indicated stable cells infected with shRNA against USP11 or NT (scrambled). Results were normalized with NT infected corresponding controls and expressed as mean±SD (n=3). **p*<0.05, ***p*,0.01, ****p*<0.005, $^{\alpha}$*p*<0.001 vs NT infected corresponding cells.

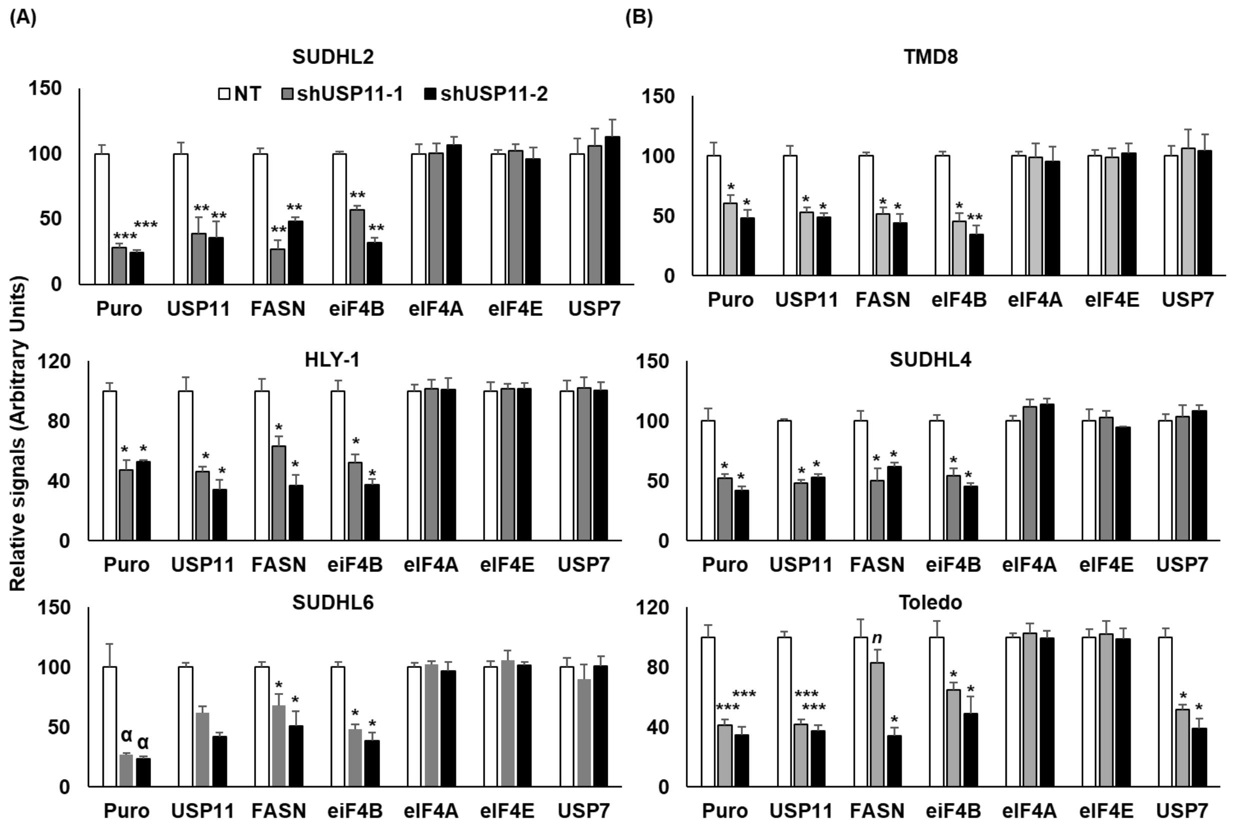

Supplementary Figure 19:

(A & B) Densitometric quantification of the immunoblots in Figure 5A (A) and Supplementary Figure 18A (B). Values were normalized with corresponding NT infected corresponding cells and were represented as mean±SD for n=3. Statistical analysis was performed using Student's *t*-test (Unpaired two tailed) ^{n}non-significant, *p<0.05, **p,0.01, ***p<0.005, $^{\alpha}p$<0.001 vs NT infected corresponding cells.

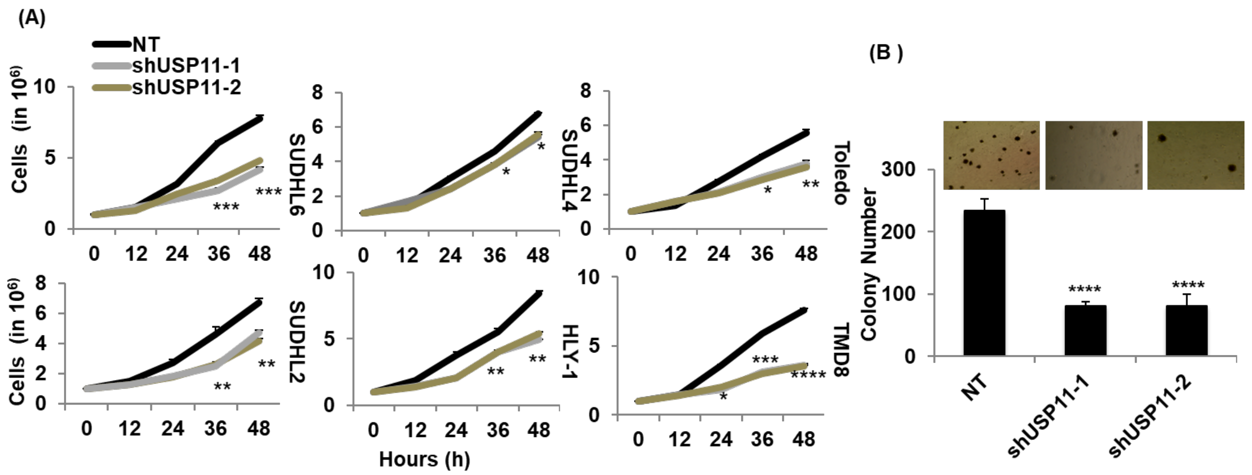

Supplementary Figure 20: USP11 is required for DLBCL proliferation

(A) Indicated USP11 depleted stable cells were seed 1 million per well in 6 well plate. Post 12h cells were collected and counted using trypan blue. Values are expressed as mean±SD (n=3). *p<0.05, **p,0.01, ***p<0.005, ****p<0.001 vs. NT infected corresponding cells. (B) Total number of colonies grown in USP11 depleted SUDHL6 cells in methylcellulose culture. Colony counts were performed on the 15th day of methylcellulose culture. ****p<0.001 vs NT infected cells.

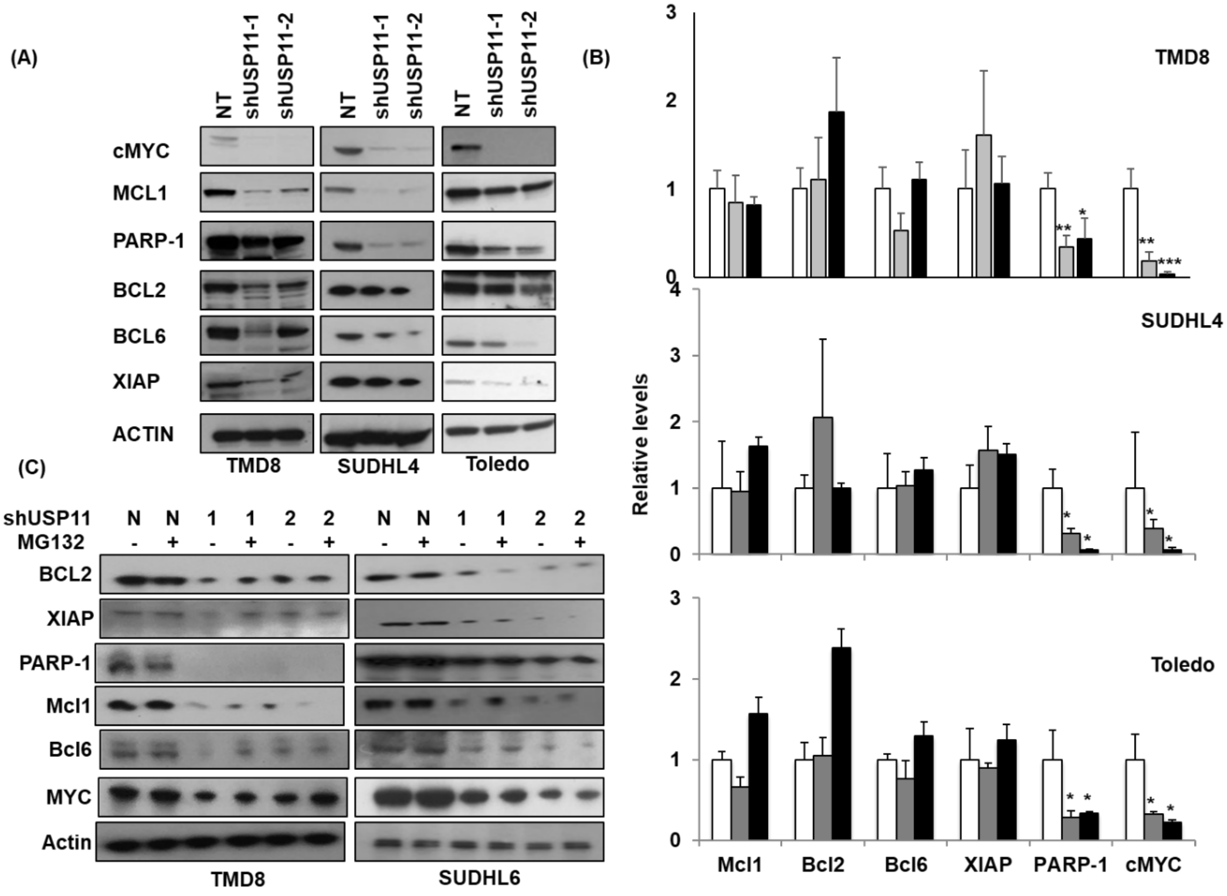

Supplementary Figure 21: USP11 expression is required for eIF4B-sensitive gene translation

(A) Indicated USP11 knockdown cell lysates were probed for indicated antibodies. Actin was used as loading control. (B) qRT-PCR analysis for checking mRNA expression of indicated genes in USP11 knockdown cells. Results were normalized with NT infected corresponding controls and expressed as mean±SD (n=3). *p<0.05, **p,0.01, ***p<0.005 vs NT infected corresponding cells. (C) Exponentially growing USP11 depleted TMD8 & SUDHL6 cells were treated with MG132 (10μM) for 2 hours and lysates were probed for the indicated antibodies. Actin was used as loading control.

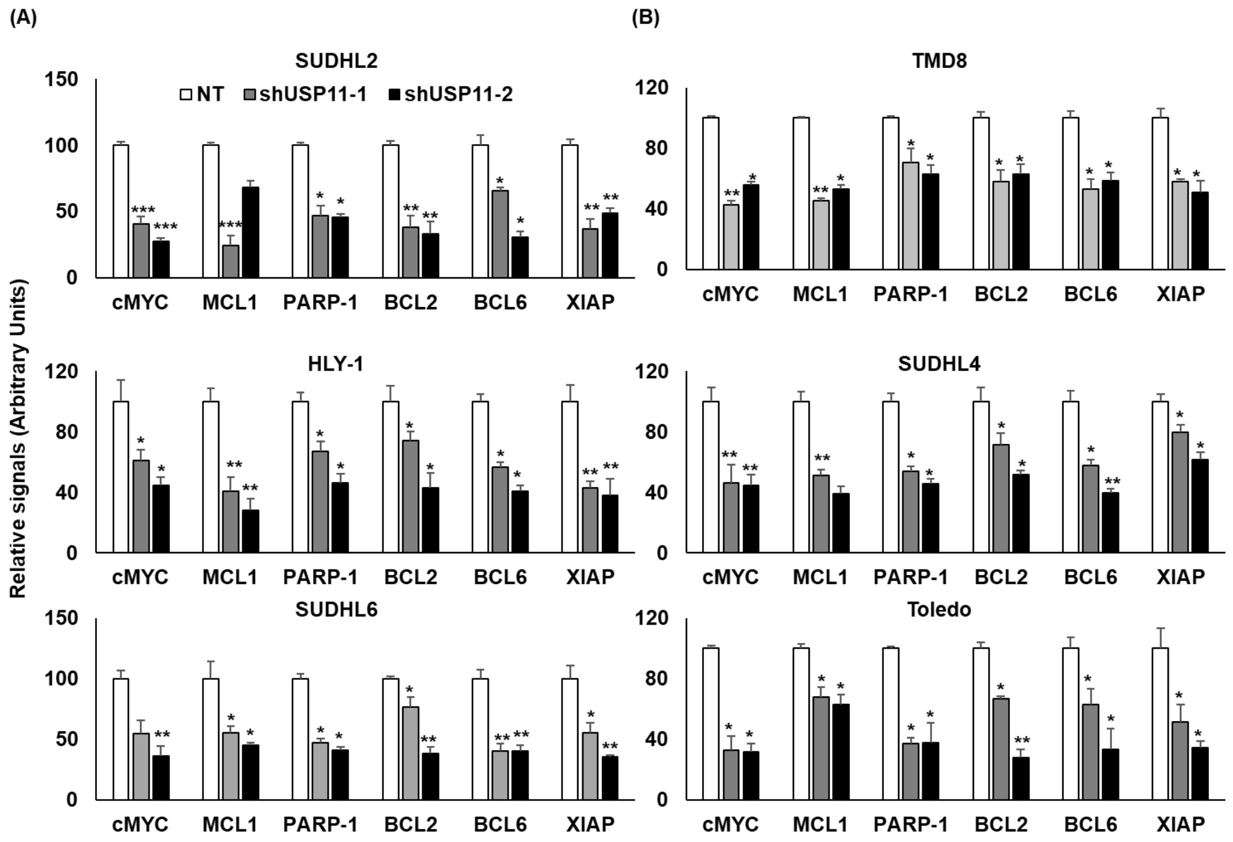

Supplementary Figure 22:

(A & B) Densitometric quantification of the immunoblots in Figure 6D and Supplementary Figure 21A (B). Values were normalized with corresponding NT infected corresponding cells and were represented as mean±SD for n=3. Statistical analysis was performed using Student's *t*-test (Unpaired two tailed) *p<0.05, **p,0.01, ***p<0.005 vs NT infected corresponding cells.

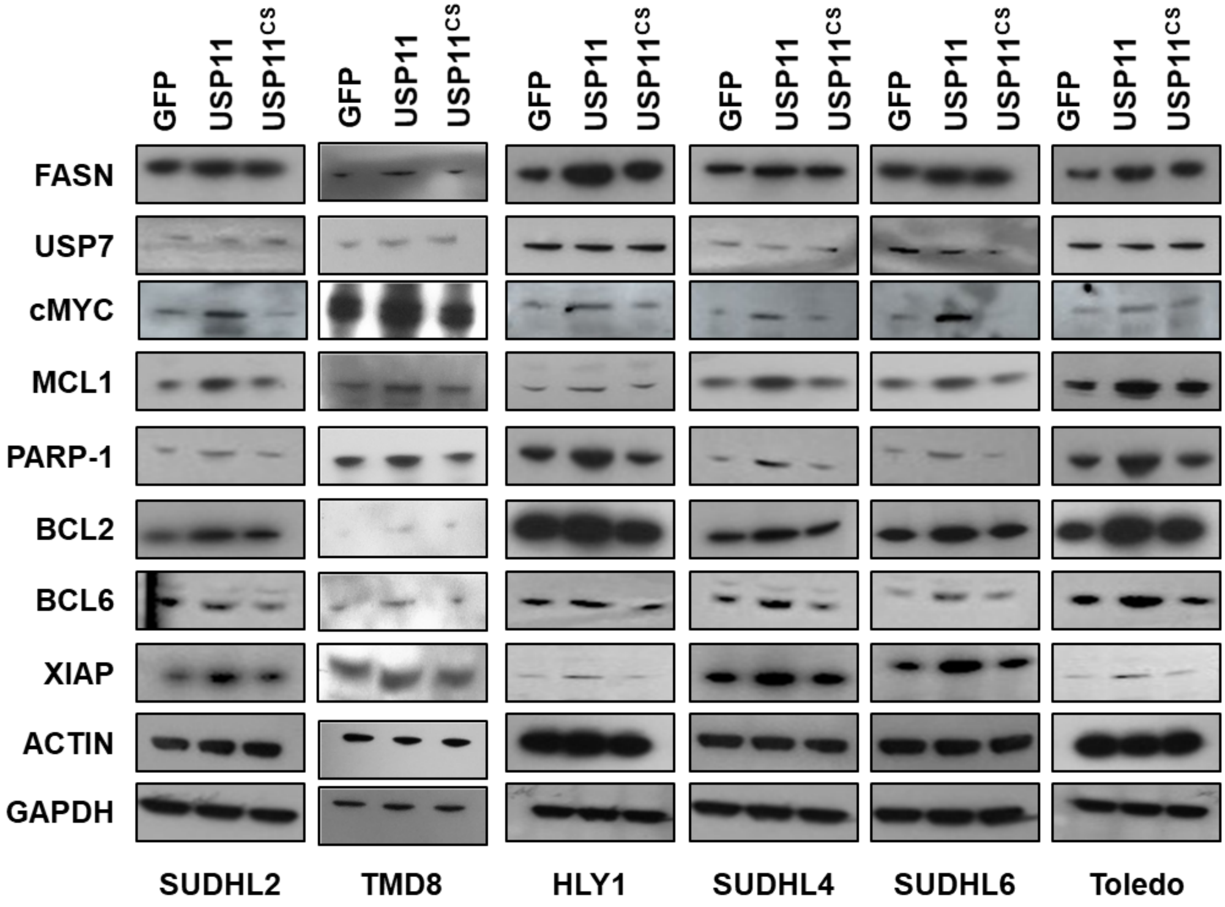

Supplementary Figure 23: Active USP11 promotes eIF4B-sensitive translation.

Indicated stable cells overexpressing GFP or USP11 (wt or catalytically inactive) were lysed and probed for indicated antibodies.

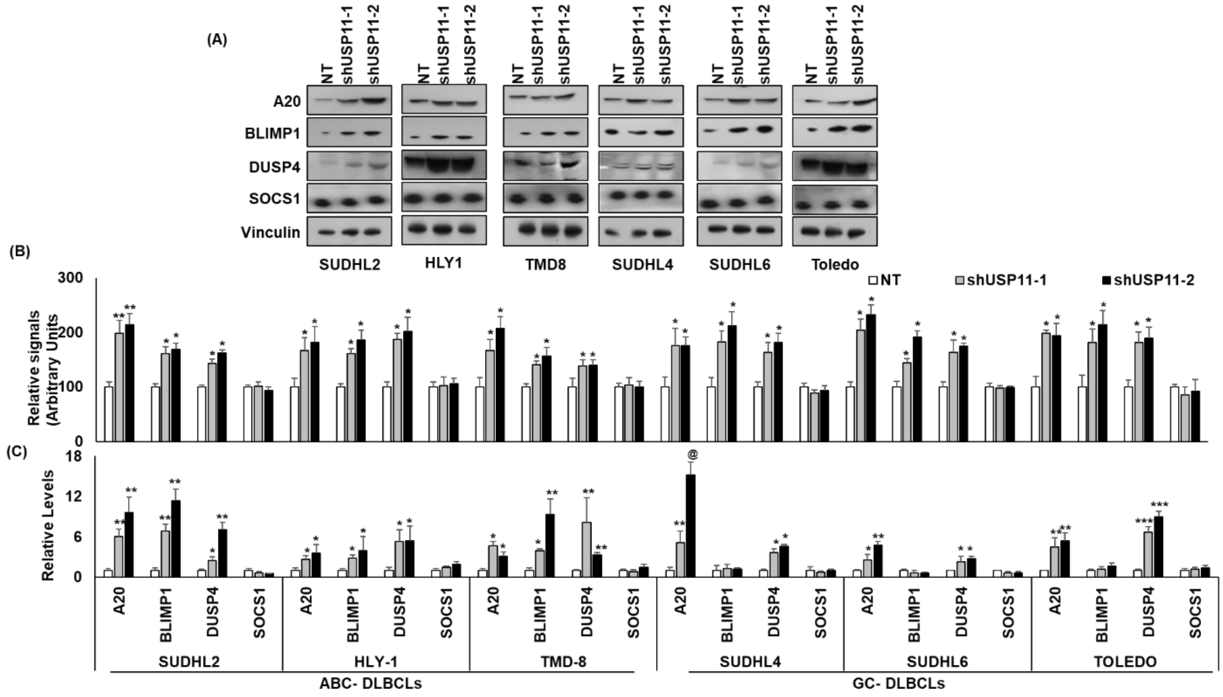

Supplementary Figure 24: USP11 depletion enhances tumor suppressor genes expression

(A) Indicated USP11 depleted stable cells were lysed and lysates were probed for the defined antibodies. (B) Densitometric quantification of the immunoblots in Supplementary Figure 24A. Values were normalized with corresponding NT infected corresponding cells and were represented as mean±SD for n=3. Statistical analysis was performed using Student's *t*-test (Unpaired two tailed) $*p<0.05$, $**p$,0.01, vs NT infected corresponding cells. (C) qRT-PCR analysis for expression of defined genes in indicated stable cells infected with shRNA against USP11 or NT (scrambled). Results are expressed as mean±SD (n=3). Statistical analysis was performed using Student's *t*-test (Unpaired two tailed) $*p<0.05$, $**p<0.01$, $^{\alpha}p<0.001$ vs NT infected corresponding cells.

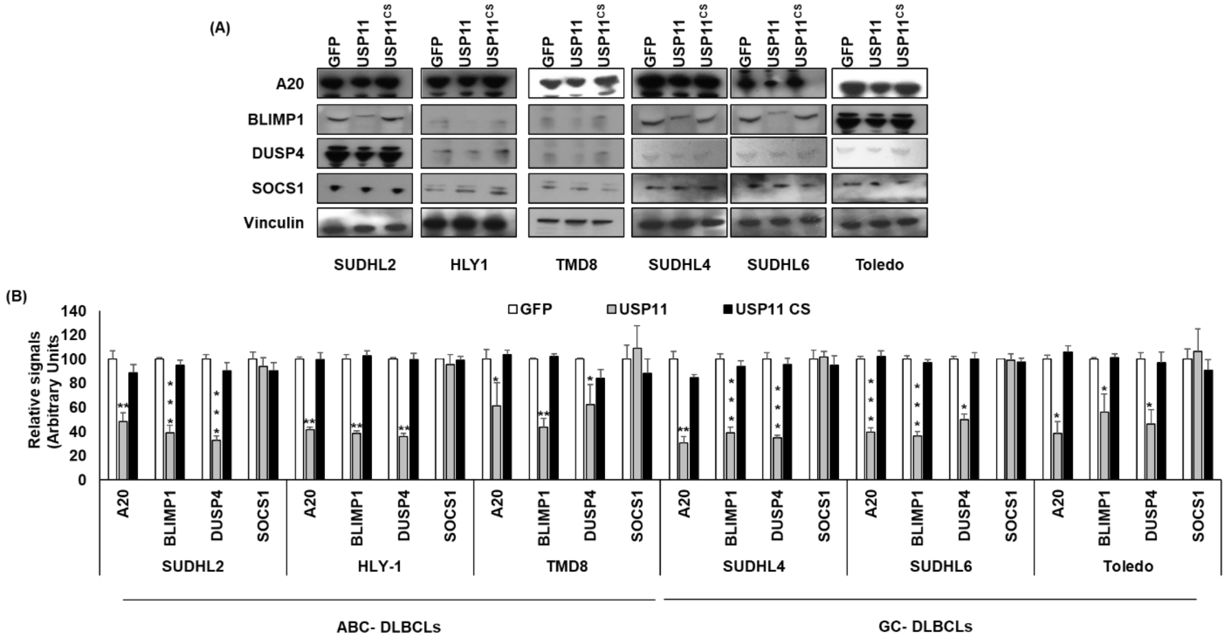

Supplementary Figure 25: Active USP11 depletes tumor suppressor genes expression

(A) Indicated USP11 overexpressing stable cells were lysed and lysates were probed for the defined antibodies. (B) Densitometric quantification of the immunoblots in Supplementary Figure 25A. Values were normalized with corresponding GFP infected cells and were represented as mean±SD for n=3. Statistical analysis was performed using Student's *t*-test (Unpaired two tailed) *p<0.05, **p,0.01, ***p<0.005 vs GFP infected corresponding cells.

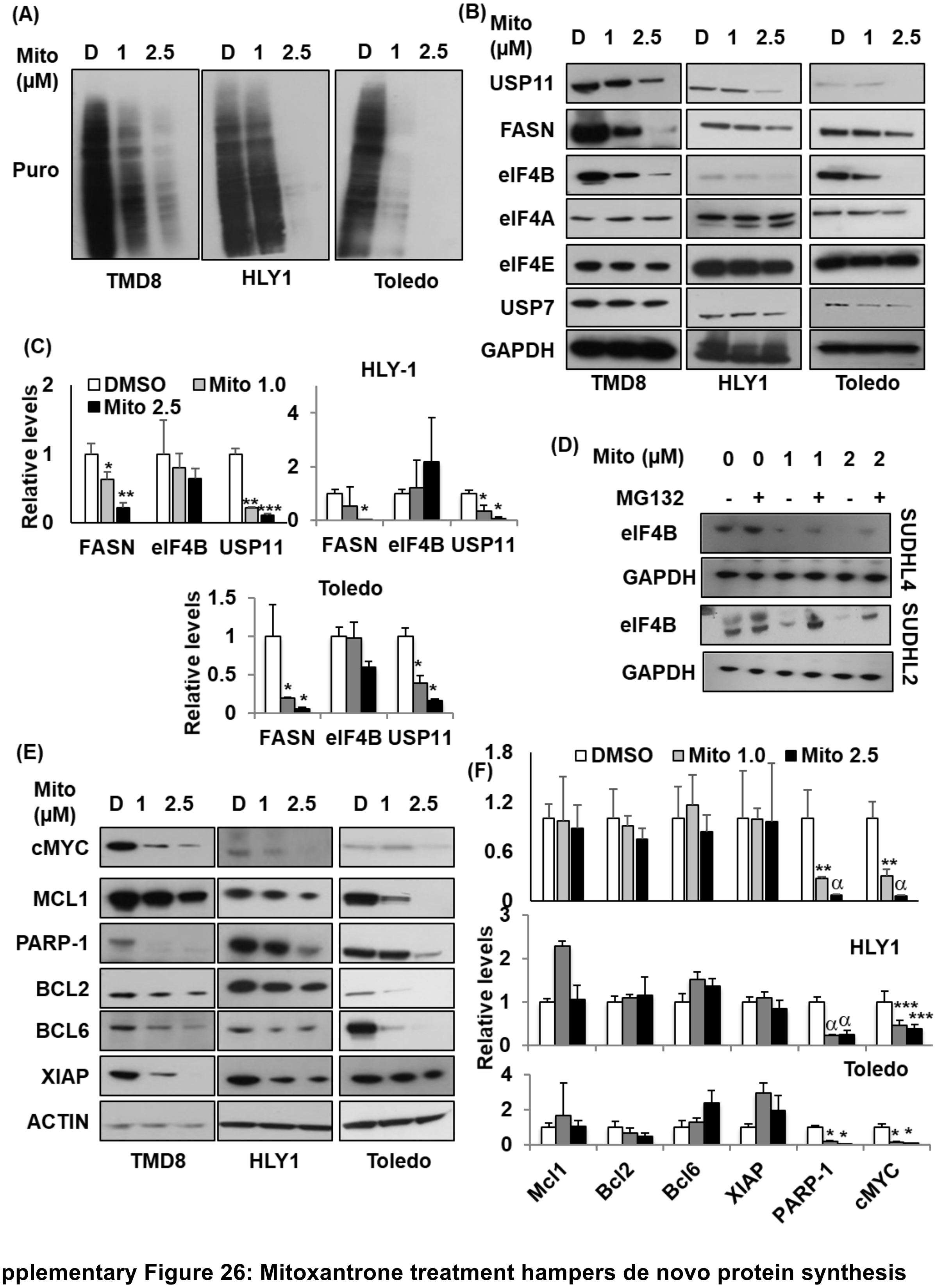

Supplementary Figure 26: Mitoxantrone treatment hampers de novo protein synthesis

(A & B) Indicated cells were cultured with Mito (indicated concentration) for 14h. Post treatment, cells were incubated with puromycin (1µg/ml) and lysates were probed with anti-puromycin for SUnSET assay (A). Same lysates were also probed for indicated antibodies (B). GAPDH was used as loading control. (C) qRT-PCR analysis of FASN, eIF4B and USP11 expression in indicated cells treated with defined concentration of Mito. Results were normalized with DMSO treated corresponding cells and expressed as mean±SD (n=3). Statistical analysis was performed using Student's *t*-test (Unpaired two tailed) *$p<0.05$, **$p<0.01$, ***$p<0.005$ vs DMSO treated corresponding cells. (D) SUDHL4 and SUDHL2 were treated with Mito (indicated concentration) for 14 h followed by treatment with MG132 (10µM) for 2 hours. Lysates were probed for the indicated antibodies. (E) Lysates (from A & B) were resolved and probed for indicated antibodies. Actin was loading control. (F) qRT-PCR analysis of studied genes in indicated cells treated with defined concentration of Mito. Results were normalized with DMSO treated corresponding cells and expressed as mean±SD (n=3). Statistical analysis was performed using Student's *t*-test (Unpaired two tailed) *$p<0.05$, **$p<0.01$, ***$p<0.005$, $^{\alpha}p<0.001$ vs. DMSO treated corresponding cells.

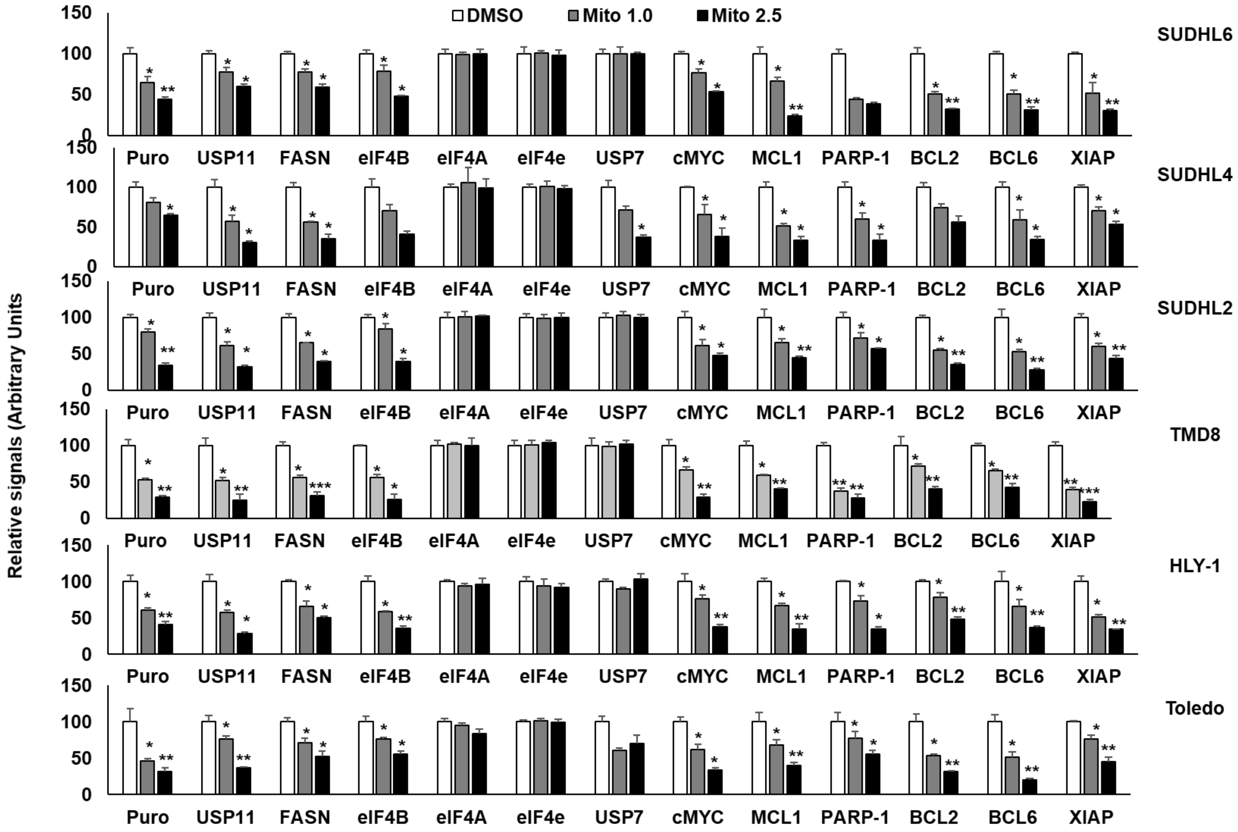

Supplementary Figure 27:

Densitometric quantification of the immunoblots in Figure 7B, 7C, 7E, Supplementary Figure 26A, 26B & 26E. Values were normalized with vehicle control, DMSO (0.1%) and expressed as mean±SD (n=3). Statistical analysis was performed using Student's *t*-test (Unpaired two tailed) *$p<0.05$, **p,0.01 vs DMSO treated corresponding cells.

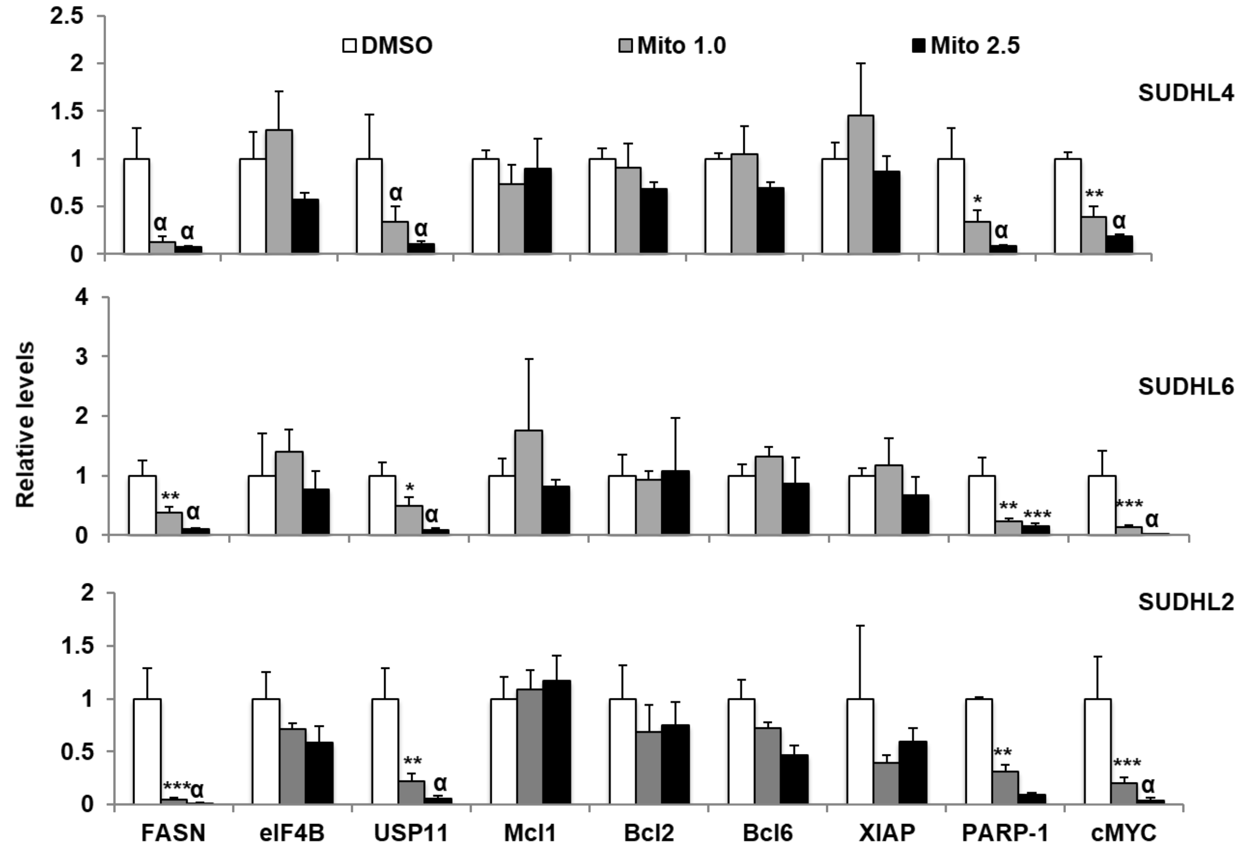

Supplementary Figure 28:

qRT-PCR analysis of studied genes in indicated cells treated with defined concentration of Mito. Results were normalized with DMSO treated corresponding cells and expressed as mean±SD (n=3). Statistical analysis was performed using Student's *t*-test (Unpaired two tailed) *$p<0.05$, **$p<0.01$ ***$p<0.005$, $^{\alpha}p<0.001$ vs DMSO treated corresponding cells.

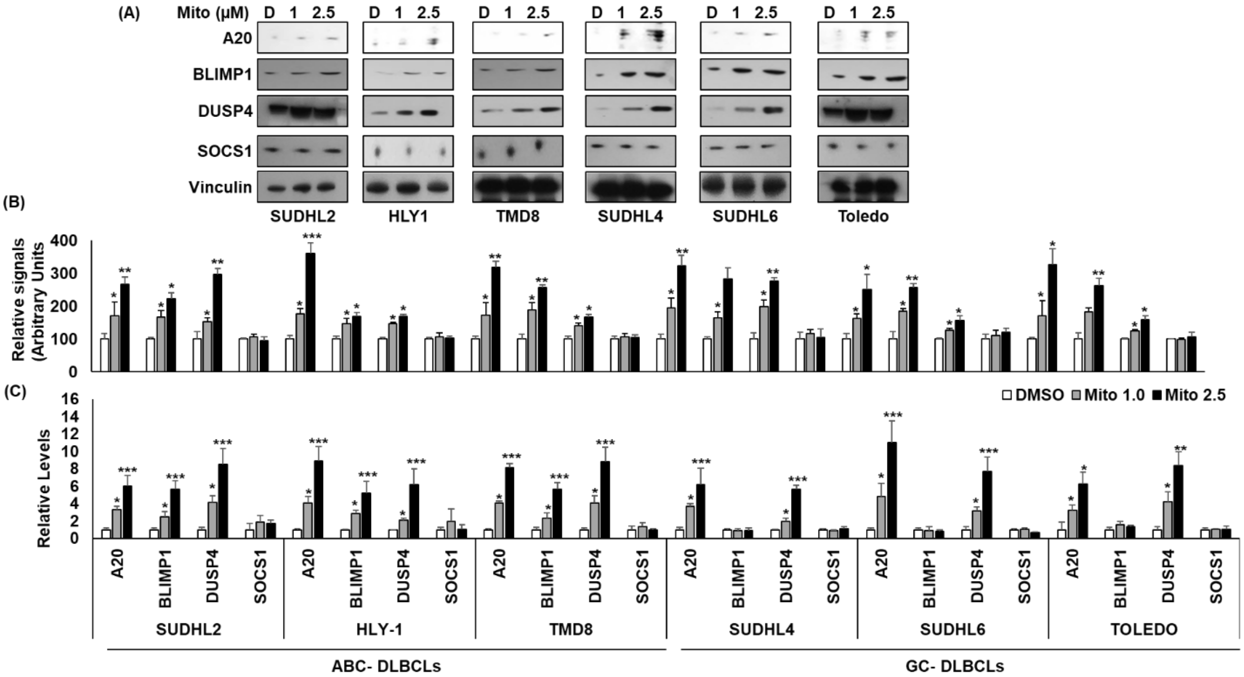

Supplementary Figure 29: Mitoxantrone treatment enhances tumor suppressor genes expression

(A) Indicated cells were treated with Mito in increasing concentration for 16h followed by lysis and lysates were probed for the defined antibodies. (B) Densitometric quantification of the immunoblots in Supplementary Figure 29A. Values were normalized with corresponding DMSO treated cells and were represented as mean±SD for n=3. Statistical analysis was performed using Student's *t*-test (Unpaired two tailed) **p*<0.05, ***p*,0.01, vs DMSO treated corresponding cells. (C) qRT-PCR analysis for expression of defined genes in indicated cells treated with Mito. Results are expressed as mean±SD (n=3). Statistical analysis was performed using Student's *t*-test (Unpaired two tailed) **p*<0.05, ***p*,0.01, ****p*<0.005 vs DMSO treated corresponding cells.

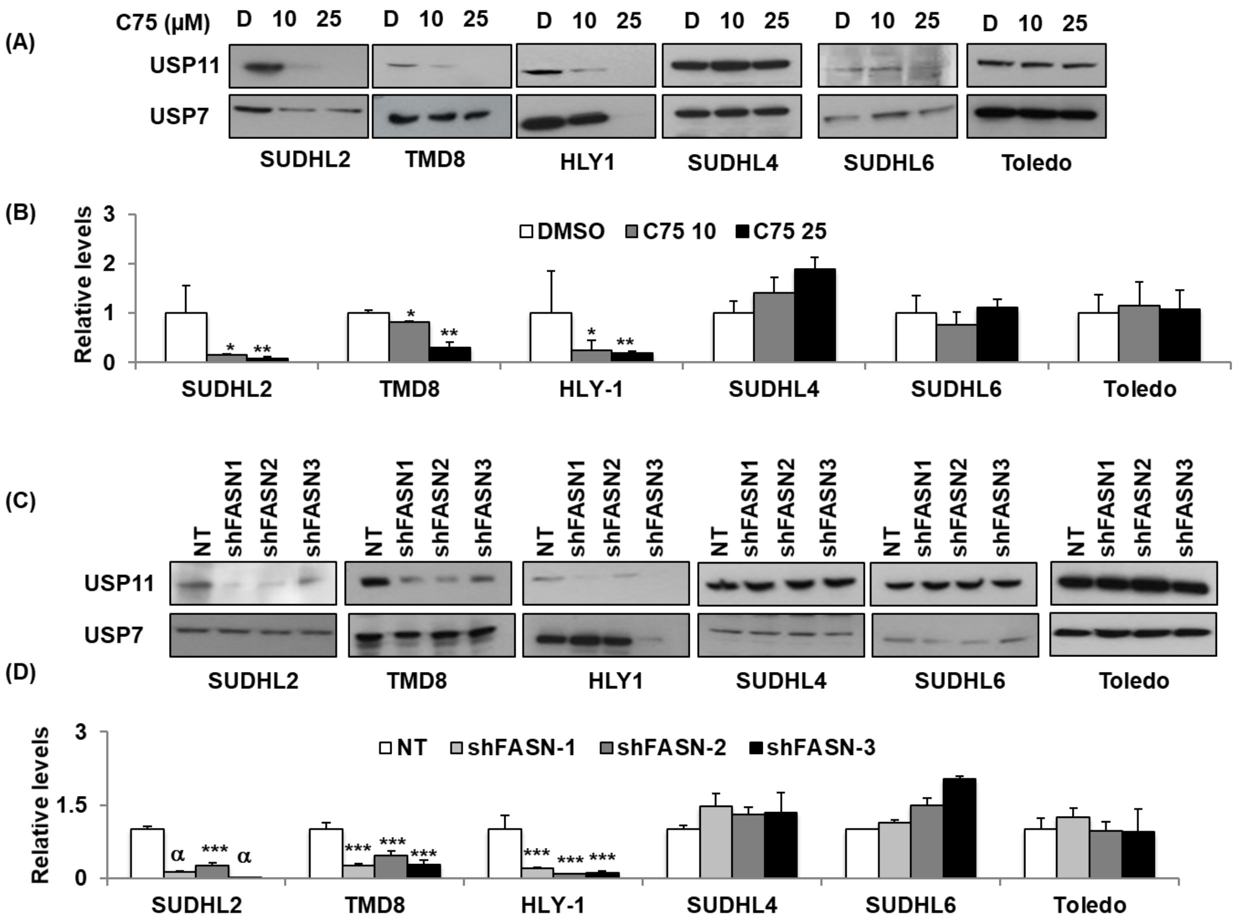

Supplementary Figure 30: FASN inhibition depletes USP11 in ABC-DLBCLs.

(A) Lysates of Supplementary Figure 8A were probed for USP11 and USP7. (B) qRT-PCR analysis of USP11 expression in indicated cells treated with defined concentration of C75. Results are expressed as mean±SD (n=3). Statistical analysis was performed using Student's *t*-test (Unpaired two tailed) *p<0.05, **p,0.01, vs DMSO treated corresponding cells. (C) Lysates of Supplementary figure 8B were probed for USP11 and USP7. (D) qRT-PCR analysis of USP11 expression in indicated FASN depleted cells. Results are expressed as mean±SD (n=3). Statistical analysis was performed using Student's *t*-test (Unpaired two tailed) *p<0.05, **p,0.01 vs NT infected corresponding cells.

.5

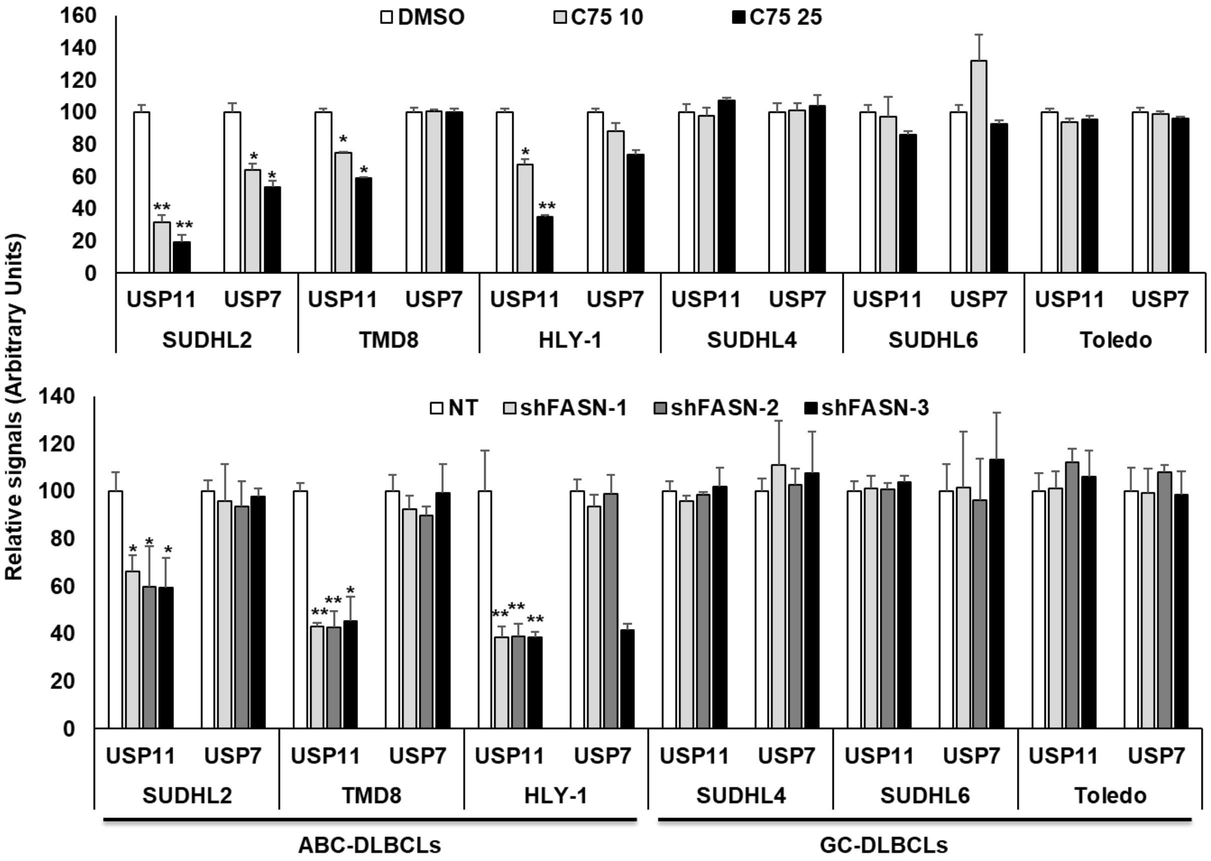

Supplementary Figure 31:

(A) Densitometric quantification of the immunoblots in Supplementary Figure 30A. Values were normalized with corresponding DMSO treated corresponding cells and were represented as mean±SD for n=3. Statistical analysis was performed using Student's *t*-test (Unpaired two tailed) *p<0.05, **p,0.01 vs DMSO treated corresponding cells. (B) Densitometric quantification of the immunoblots in Supplementary Figure 30B. Values were normalized with corresponding NT infected corresponding cells and were represented as mean±SD for n=3. Statistical analysis was performed using Student's *t*-test (Unpaired two tailed) *p<0.05, **p,0.01 vs NT infected corresponding cells.

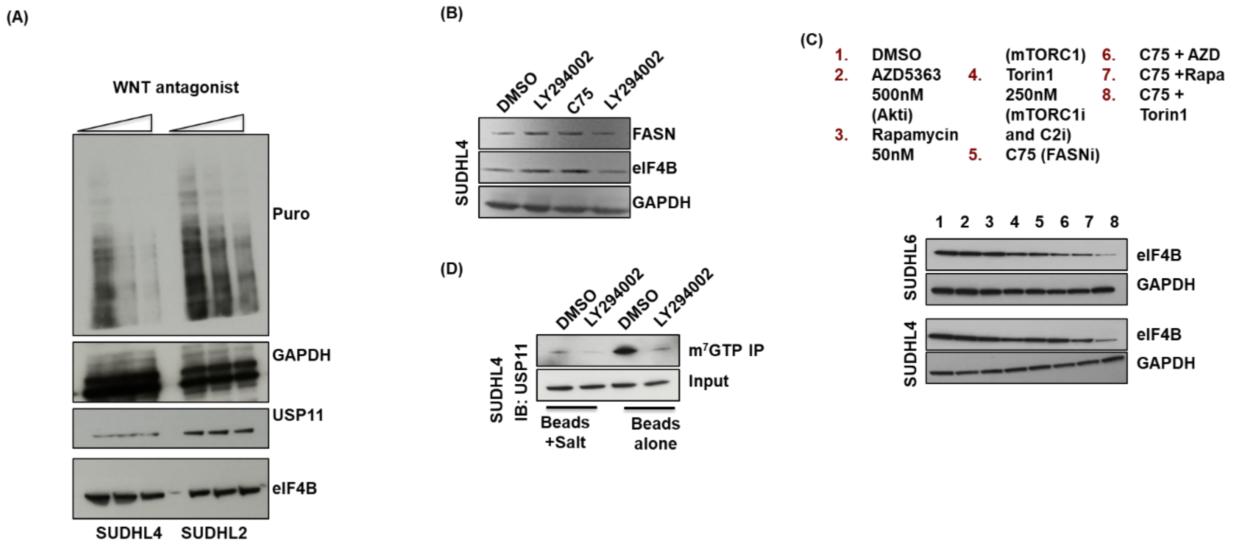

Supplementary Figure 32:

(A) Wnt signaling doesn't regulate eIF4B-USP11 axis. Indicated cells were treated with Wnt antagonist for 14 h. Post treatment cells were exposed to puromycin (1µg/ml) for 30 min and lysates were probed for indicated antibodies. GAPDH was used as loading control. (B) **PI3K regulates USP11-eIF4B interaction.** SUDHL4 cells were treated with C75 (FASN inhibitor, 25µM) and LY294002 (PI3K inhibitor, 1µM) either alone or in combination for 14h. Post treatment lysates were probed for indicated antibodies. GAPDH was used as loading control. (C). SUDHL4 and SUDHL6 were treated with AZD5363 (Akt inhibitor, 500nM), Rapamycin (mTOR inhibitor, 50nM), Torin 1(mTOR inhibitor, 250nM) or C75 (FASN inhibitor, 25µM) either alone or in combination as indicated. Post treatment cell lysates were probed for eIF4B. GAPDH was used as loading control. (D) SUDHL4 cells were treated with LY29002(1µM) for 4 h. Post treatment cell lysates were incubated with m^7GTP-agarose beads in presence or absence of m^7GTP salt. Co-eluted proteins were resolved and probed for USP11.

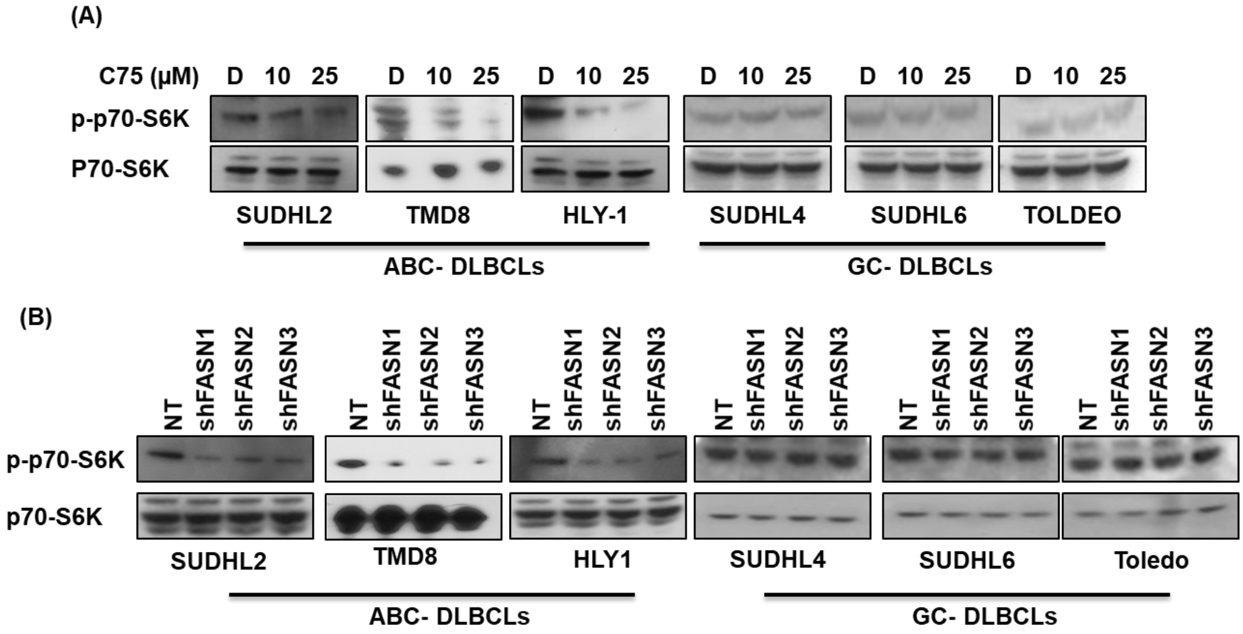

Supplementary Figure 33: FASN activity regulates p70-S6Kinase activity.

(A & B) Lysates of Supplementary figure 8A and 8B were probed for phospho-p70-S6Kinase and total p70-S6Kinase.

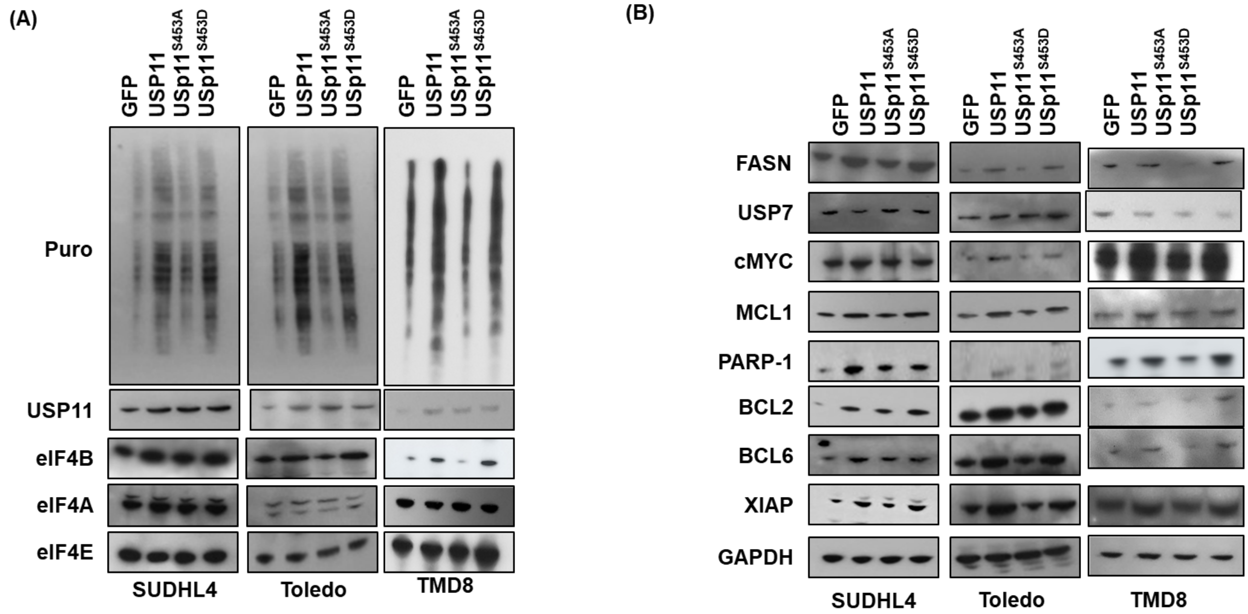

Supplementary Figure 34: S6Kinase-mediated USP11 phosphorylation promotes overall protein translation.

(A & B) DLBCL cells infected with USP11 (wt and indicated mutants) were cultured in presence of puromycin (3μg/mL) for 30 min and lysed. GFP infected cells were used as corresponding controls. Total cell lysates were resolved and probed for indicated antibodies. GAPDH was used as loading control.

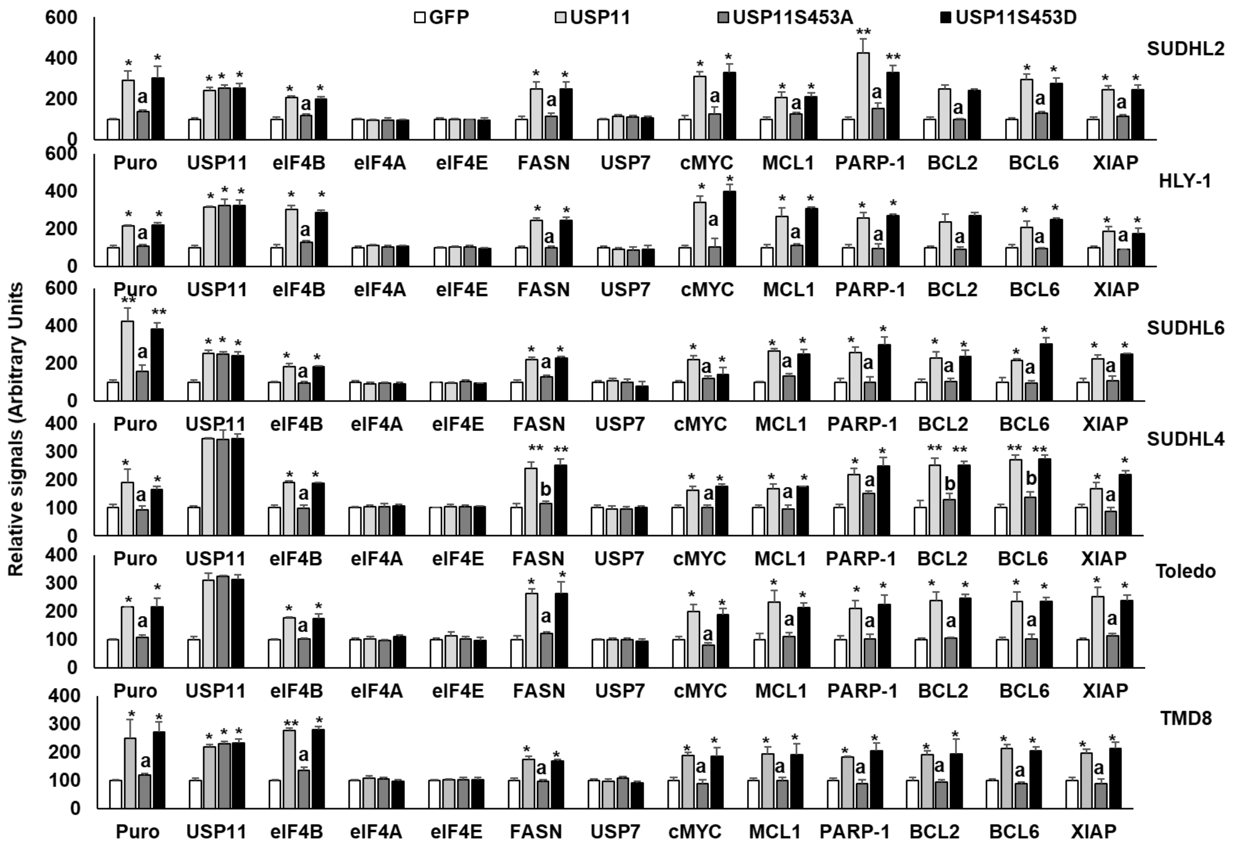

Supplementary Figure 35:

Densitometric quantification of the immunoblots in Figure 8K & 8L and Supplementary Figure 34A & 34B. Values were normalized with corresponding GFP infected corresponding cells and were represented as mean±SD for n=3. Statistical analysis was performed using one-way ANOVA followed by Bonferroeni's post hoc test. **p*<0.05, ***p*,0.01 vs GFP infected corresponding cells, [a]*p*<0.05, [b]*p*<0.01 vs USP11 (w) infected corresponding cells.

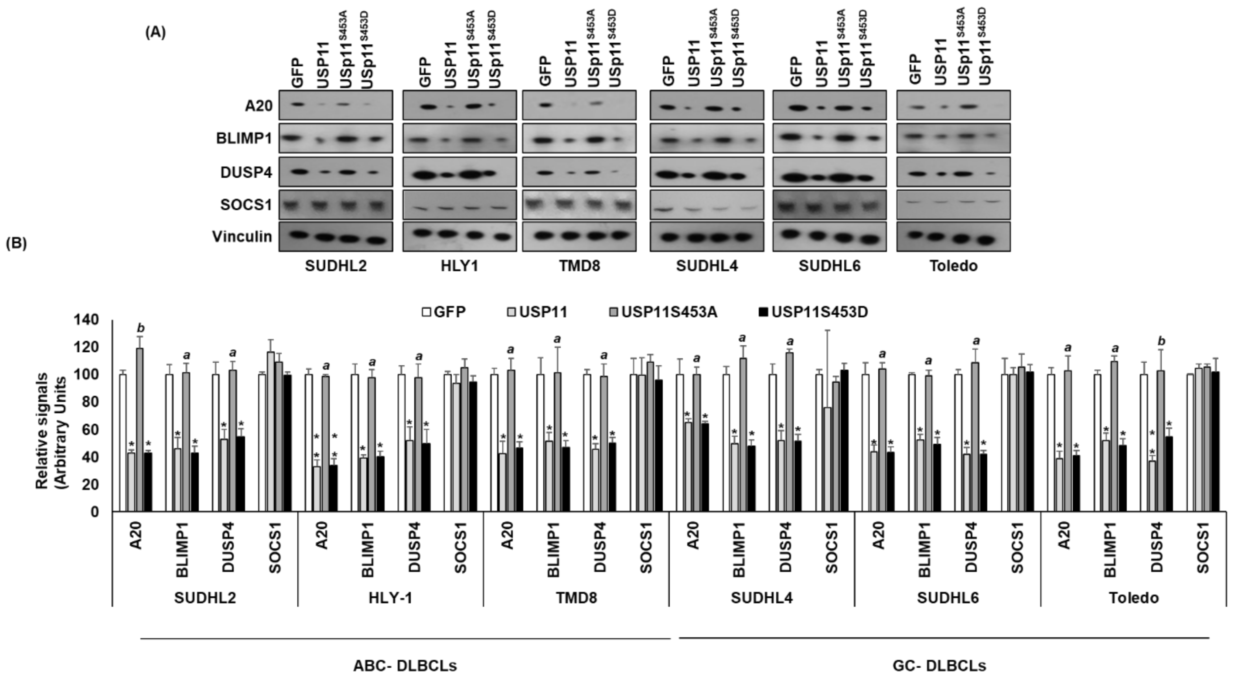

Supplementary Figure 36: S6Kinase-mediated USP11 phosphorylation depletes tumor gene expression

(A) Indicated infected cells were lysed and lysates were probed for the defined antibodies. (B) Densitometric quantification of the immunoblots in Supplementary Figure 36A. Values were normalized with corresponding GFP infected corresponding cells and were represented as mean±SD for n=3. Statistical analysis was performed using one-way ANOVA followed by Bonferroeni's post hoc test. **p*<0.05, ***p*,0.01 vs GFP infected corresponding cells, [a]*p*<0.05, [b]*p*<0.01 vs USP11 (w) infected corresponding cells.

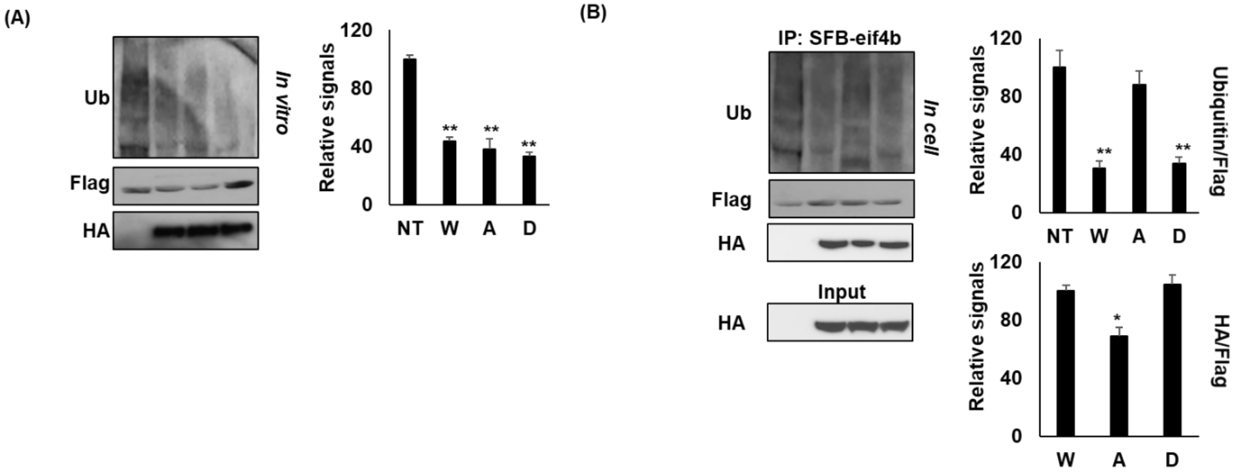

Supplementary Figure 37: **S6Kinase-mediated USP11 phosphorylation enhances eIF4B interaction**

(A) In vitro deubiquitination assay of eIF4B. Polyubiqitinated eIF4B, HA tagged USP11 and its mutant were enriched from 293T (see experimental procedure for details); incubated with buffered conditions for deubiquitination. Post reaction lysates were probed for the indicated antibodies. NT; control, W; USP11 (wt), A; $USP11^{S453A}$, D; $USP11^{S453D}$. Bar diagram represents the densitometric analysis of the blots. Values were normalized with flag signals and were represented as mean±SD. Statistical analysis was performed using Student's *t*-test (Unpaired two tailed), $**p<0.01$ vs NT (B) 293T cells were transfected with SFB-eIF4B alone or in combination with USP11 and its Mutants. Post transfection cells were treated with C75 (25μM) for 6 hours and eIF4B was enriched using streptavidin beads. Level of ubiquitin was detected using specific antibody. eIF4B enrichment was confirmed by probing with flag and USP11 was confirmed by probing with HA antibody. NT; GFP transfection control, W; USP11 (wt), A; $USP11^{S453A}$, D; $USP11^{S453D}$. Bar diagrams represents the densitometric analysis of the indicated blots. Values were normalized with flag signals and were represented as mean±SD. Statistical analysis was performed using Student's *t*-test (Unpaired two tailed), $*p<0.05$, vs W, $**p<0.01$ vs NT.

Figure 2E

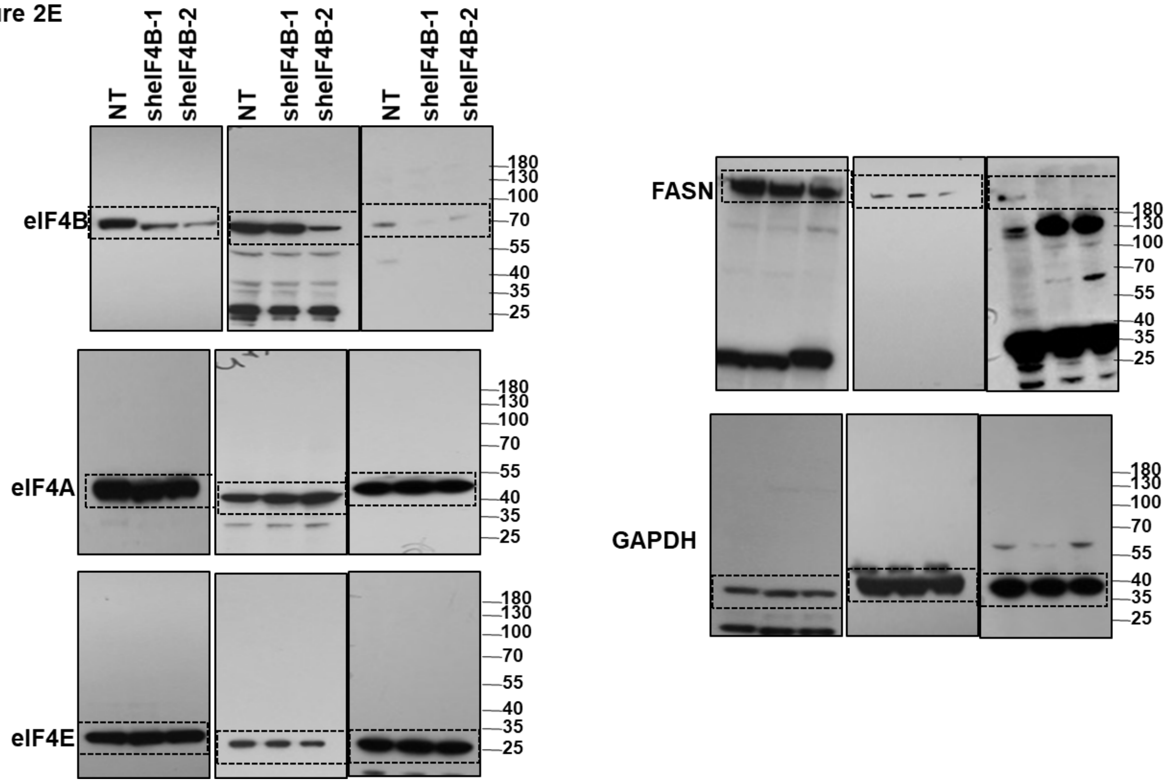
NT
sheIF4B-1
sheIF4B-2
eIF4B
eIF4A
eIF4E
FASN
GAPDH
180
130
100
70
55
40
35
25

Figure 3A

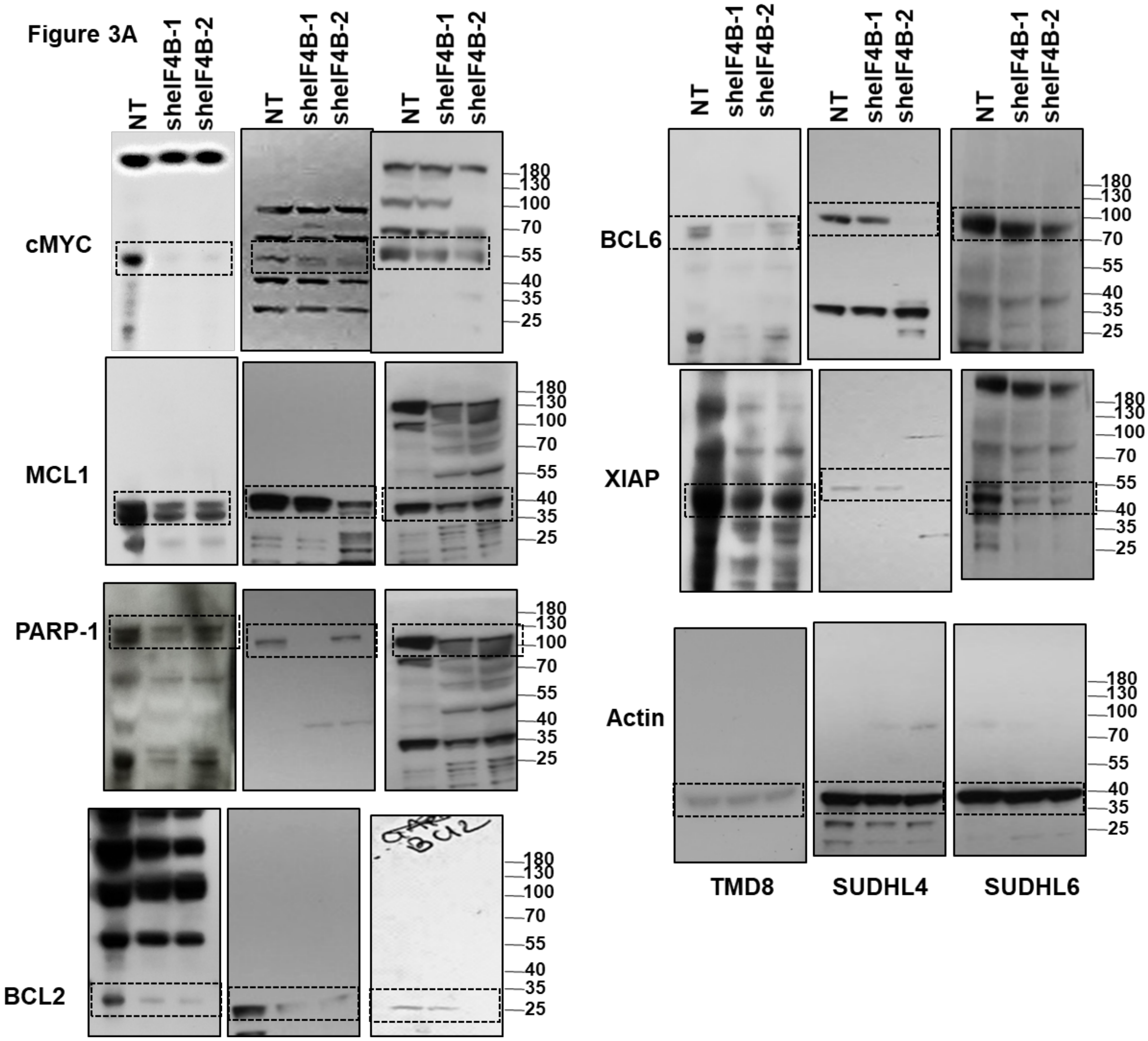

NT
sheIF4B-1
sheIF4B-2
cMYC
MCL1
PARP-1
BCL2
BCL6
XIAP
Actin
180
130
100
70
55
40
35
25
TMD8
SUDHL4
SUDHL6

Figure 3C

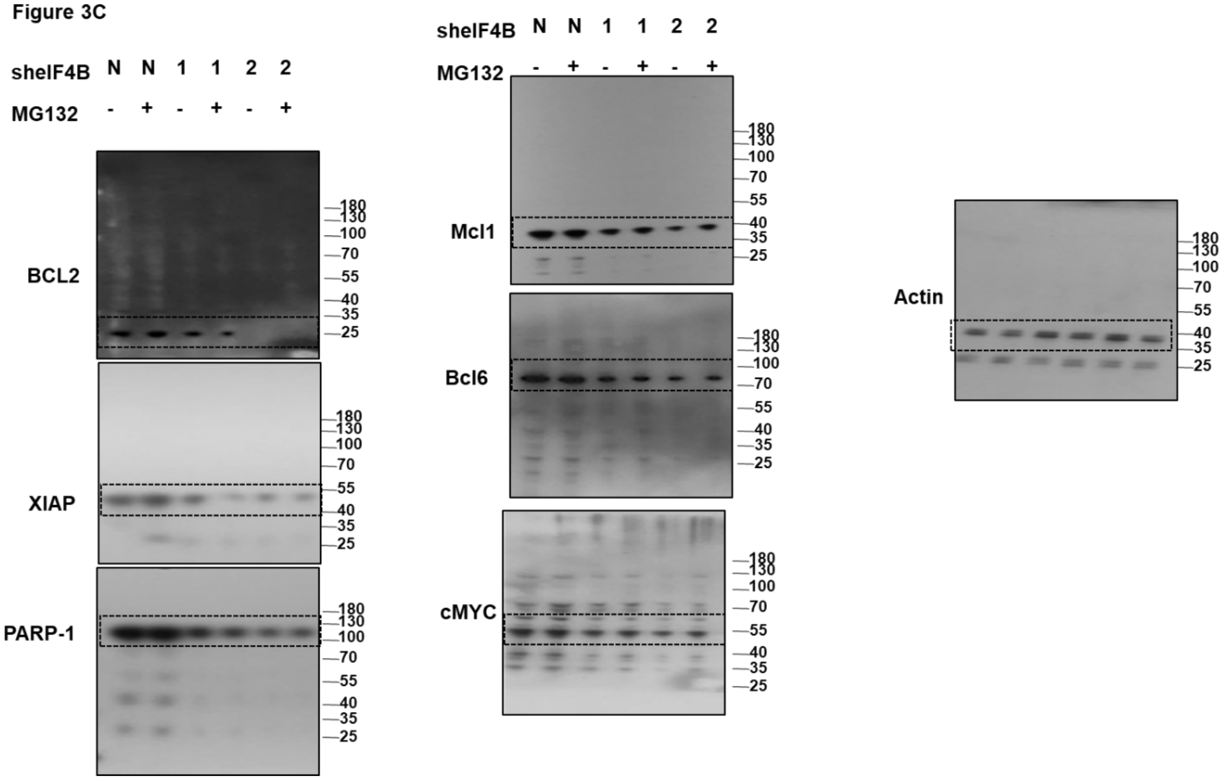
shelF4B N N 1 1 2 2
MG132 - + - + - +
BCL2
XIAP
PARP-1
Mcl1
Bcl6
cMYC
Actin
180
130
100
70
55
40
35
25

Figure 3E

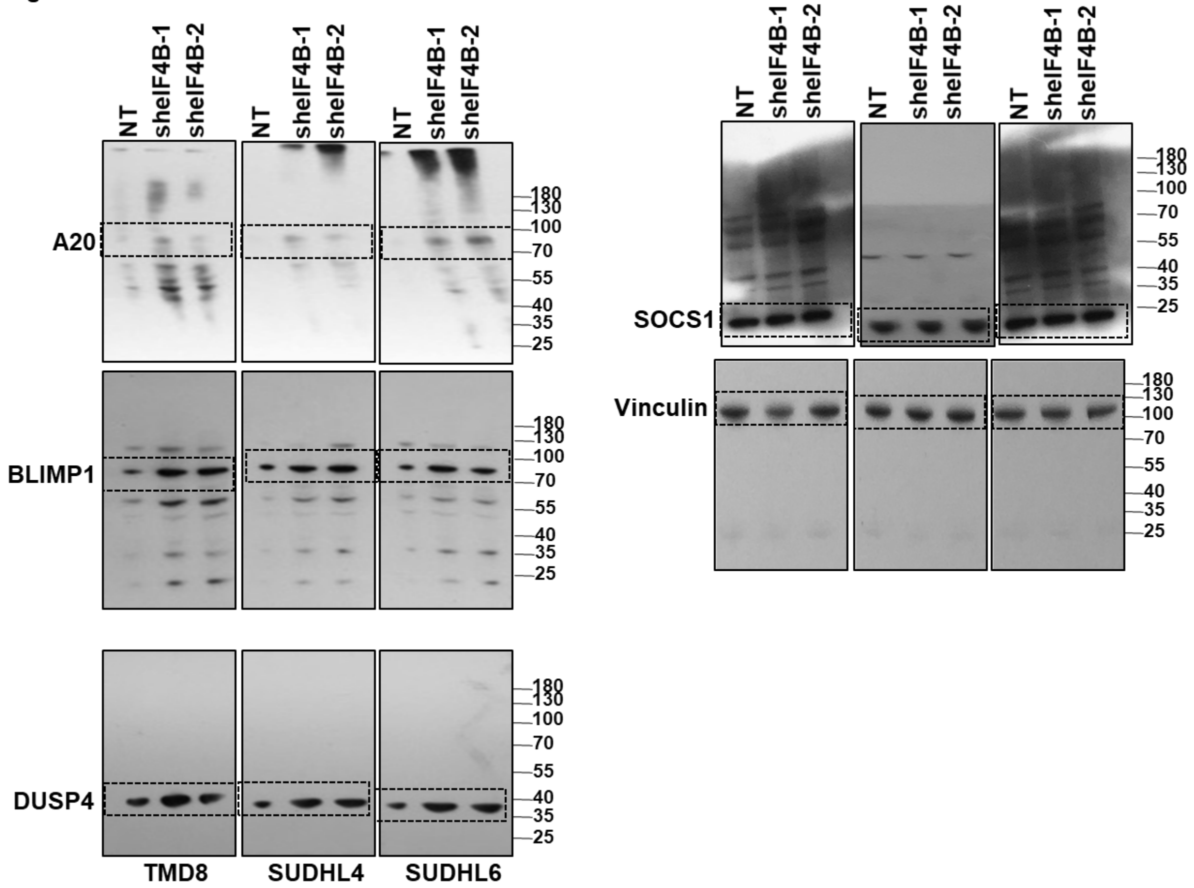
NT
shelF4B-1
shelF4B-2
A20
BLIMP1
DUSP4
TMD8
SUDHL4
SUDHL6
SOCS1
Vinculin
180
130
100
70
55
40
35
25

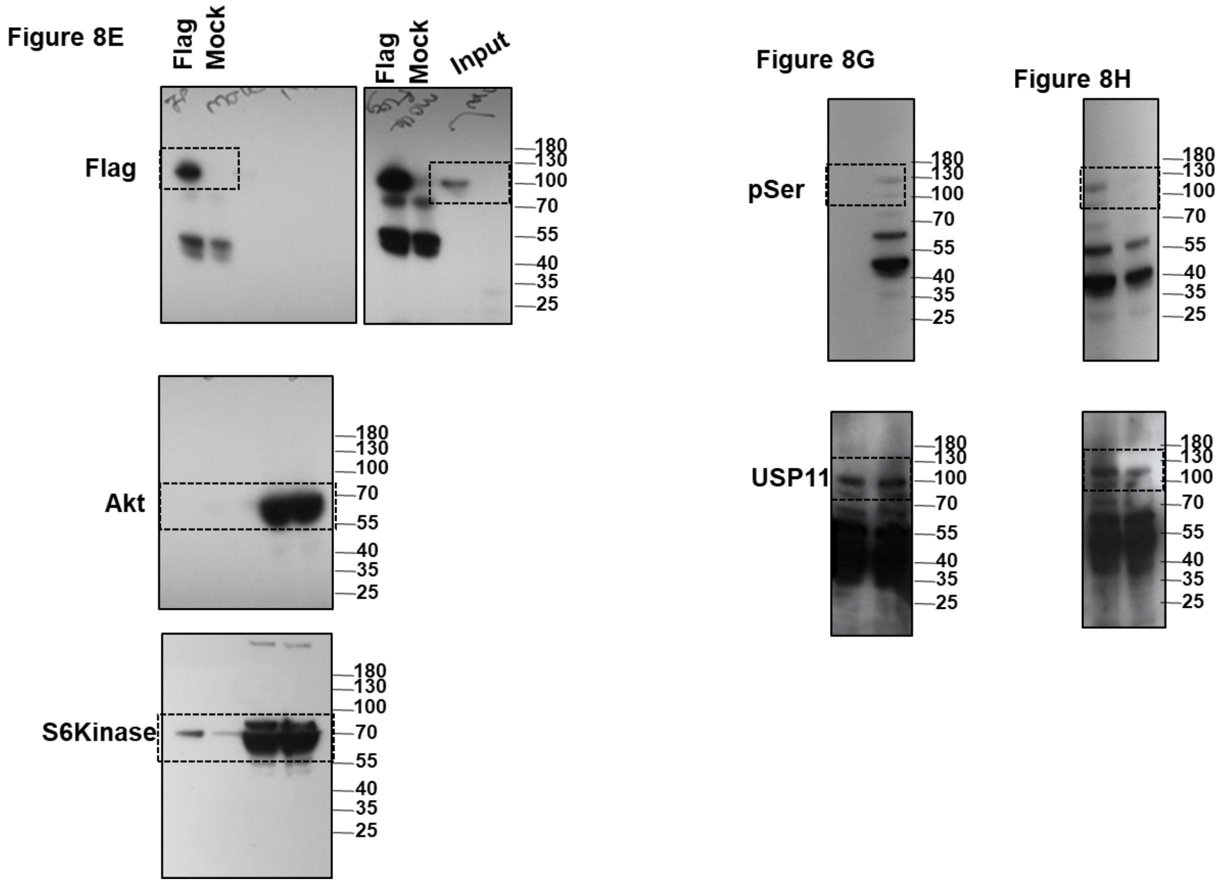

Supplementary Figure 38: Uncropped images of as shown in the respective figures. Highlighted dotted blackbox are shown in the figure in the main text.
